# Complex Deleterious Interactions Associated with Malic Enzyme May Contribute to Reproductive Isolation in the Copepod *Tigriopus californicus*

**DOI:** 10.1371/journal.pone.0021177

**Published:** 2011-06-22

**Authors:** Christopher S. Willett

**Affiliations:** Department of Biology, University of North Carolina, Chapel Hill, North Carolina, United States of America; University of Cambridge, United Kingdom

## Abstract

Dobzhansky-Muller incompatibilities can result from the interactions of more than a single pair of interacting genes and there are several different models of how such complex interactions can be structured. Previous empirical work has identified complex conspecific epistasis as a form of complex interaction that has contributed to postzygotic reproductive isolation between taxa, but other forms of complexity are also possible. Here, I probe the genetic basis of reproductive isolation in crosses of the intertidal copepod *Tigriopus californicus* by looking at the impact of markers in genes encoding metabolic enzymes in F_2_ hybrids. The region of the genome associated with the locus *ME2* is shown to have strong, repeatable impacts on the fitness of hybrids in crosses and epistatic interactions with another chromosomal region marked by the *GOT2* locus in one set of crosses. In a cross between one of these populations and a third population, these two regions do not appear to interact despite the continuation of a large effect of the *ME2* region itself in both crosses. The combined results suggest that the *ME2* chromosomal region is involved in incompatibilities with several unique partners. If these deleterious interactions all stem from the same factor in this region, that would suggest a different form of complexity from complex conspecific epistasis, namely, multiple independent deleterious interactions stemming from the same factor. Confirmation of this idea will require more fine-scale mapping of the interactions of the *ME2* region of the genome.

## Introduction

**Figure 1:**
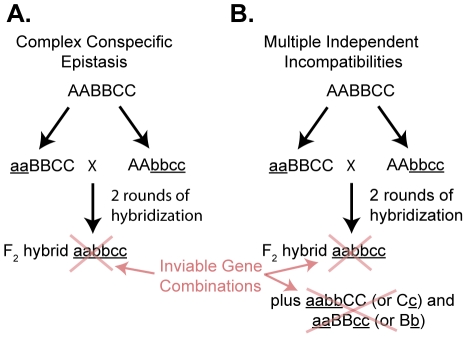
Two potential models for complex DM incompatibilities for F_2_ hybrids that involve more than two interacting loci. Both models assume that incompatibilities are recessive (i.e. lowered fitness requires derived homozygotes at each interacting locus) and that the ancestral genotype is AABBCC for the three loci. The models depict independent evolution of derived alleles (indicated by underlined lowercase letters) in two separate populations. A. Complex conspecific epistasis that in this case requires that all three loci are homozygous for the expression of a DM incompatibility. B. Multiple independent incompatibilities could stem from the same locus; here the A locus can have deleterious interactions with either the B or the C locus.

Dobzhansky-Muller (DM) incompatibilities are thought to underlie the evolution of much of the postzygotic reproductive isolation in hybrids, but the types of interactions involved in these incompatibilities are not well understood [1]. Although conceptually these DM incompatibilities are often thought of as involving pairs of interacting loci, a set of closely related theoretical models have suggested that more complex incompatibilities involving more than two partners can be easier to evolve than interactions involving only two genes [2], [3], [4]. The main argument of these models is that there are more potential pathways for evolution to follow that have no fitness valleys along them when there are more loci involved in a deleterious interaction. Empirical results have found evidence for DM incompatibilities that require three or more interacting chromosomal regions for the expression of an incompatibility [4], [5], [6], [7], [8], [9], implying that complex conspecific epistasis is underlying these incompatibilities that requires the interaction of alleles at all loci for expression of the incompatibility (Figure 1A). Interactions between three or more loci could also underlie DM incompatibilities between a pair of populations in a different fashion if the same allele at one locus is involved in multiple independent incompatibilities with different partners at two or more other loci (Figure 1B). A model developed by Kondrashov [10] suggests that with gene flow among populations, i.e. parapatry, that the initial DM incompatibility is likely to involve only a single pair of interacting loci; however, subsequent DM incompatibilities are more likely to involve interactions with the alleles in the first DM incompatibility and could involve multiple pairwise incompatibilities (as in Figure 1B) or more complex epistasis involving three or more alleles. At this point there are few clear examples from empirical studies of complexity stemming from the involvement of the same factor in multiple independent incompatibilities.

Although only a relatively small handful of specific genes involved in generating postzygotic reproductive isolation have been found from any taxa [11], [12], interestingly, several of these incompatibilities appear to be cases of complex conspecific epistasis. In the species pair *Drosophila melanogaster* and *D. simulans*, two different pairs of interacting genes have been identified that can cause hybrid lethality [13], [14]. In both cases these pairs of genes themselves are not sufficient to cause hybrid lethality but require other essential partners. A recently identified gene causing hybrid sterility from two much more closely related allopatric subspecies of *D. pseudoobscura* is also involved in complex conspecific epistatic interactions [8], [15]. Although these allopatric subspecies of *D. pseudoobscura* are unlikely to be connected by current gene flow, this type of incompatibility would be important for reproductive isolation if these recently diverged subspecies were to come into secondary contact as speciation is not complete. In cases where species have been diverged for longer periods of time many incompatibilities could have accumulated after the taxa were completely reproductively isolated via the combination of existing prezygotic and postzygotic isolation. Studies of long-divergent species pairs may not therefore reflect the likelihood for complex interactions to accumulate prior to the completion of speciation.

Genetically divergent populations of the intertidal copepod *T. californicus* provide a useful model system in which to study the evolution of DM incompatibilities before reproductive isolation is complete. This species occurs along the Pacific coast of North America and lives in upper intertidal splash pools in rocky outcrops. The populations from different rocky outcrops or different regions are often genetically differentiated from one another, reflecting limited gene flow over long periods of time [16], [17]. Divergences in mtDNA can be particularly large, with sequence divergences of over 20 percent in comparisons of populations within California [18], [19], [20]. Despite these high levels of genetic divergence among populations, there is no evidence for premating reproductive isolation among these populations [21], [22] and postmating isolation is incomplete. For crosses between populations from California, there is evidence for hybrid breakdown for both viability and fertility in F_2_ hybrids but little or no decline in fitness for F_1_ hybrids in comparison with the parental populations [23], [24], [25].

Although reproductive isolation between populations of *T. californicus* is incomplete, the genetic basis of hybrid breakdown appears to be relatively complex and might involve a number of different DM incompatibilities. Genetic markers covering each of the 12 chromosomes of this species show widespread deviations from Mendelian expectations in F_2_ hybrids from crosses between genetically divergent populations in the laboratory, suggesting a number of different genome regions are involved in deleterious interactions [26], [27]. Burton [23] found dramatic departures for two nuclear-encoded allozyme markers, with one of the allozyme loci, encoding malic enzyme (ME; EC 1.1.1.39), having near lethality for one homozygous genotypic class in a hybrid genetic background. The second allozyme, a nuclear-encoded but mitochondrial-targeted glutamate-oxaloacetate transamine (*GOT2*; note this enzyme is now called aspartate transaminase, EC 2.6.1.1), showed a non-significant trend for epistatic interactions with *ME*. Willett and Berkowitz [28] described two loci encoding homologs of ME from *T. californicus* and showed that one of these, *ME2*, showed a similar pattern of genotypic viability to that found by Burton [23] with dramatically reduced viability of one homozygous genotypic class in a hybrid background. Further implicating this genome region in an involvement in a DM incompatibility, the reduction in genotypic viability was found for F_2_ adults but not F_2_ nauplii implying that the copepods were dying during development to adults [28].

In this paper, I examine the nature of DM incompatibilities at the *ME2* and *GOT2* loci in population crosses of *T. californicus* to help determine if they might be involved in interactions with each other and other partners. First, I characterized the gene encoding the GOT2 enzyme and determined that although amino acid divergence is relatively high for this protein between copepod populations, there is no evidence for strong selection acting upon this gene. Genes that have been shown to be involved in DM incompatibilities in other systems are often subject to strong positive selection [11], [12], but sometimes have shown elevated rates of amino acid evolution without a strong signature of positive selection [15]. Second, I looked at patterns of segregation in F_2_ hybrids in a set of crosses of *T. californicus* populations for *GOT2* and two *ME* loci and I found that both *ME2* and *GOT2* have large deviations from Mendelian inheritance with *ME2* showing the near inviability of one genotypic class in a hybrid background. Finally, I tested for interactions between these loci and found that the *GOT2* gene displays strong epistatic interactions with *ME2* in one population cross but not in a cross involving a different population (despite the large effect of the *ME2* locus alone in both crosses). These results suggest that the *ME2* region of the genome is involved in DM incompatibilities in both crosses and may be involved with different partner(s) in each cross. The results of this study suggest that complexity in the genetic basis of hybrid breakdown in this system may stem from the involvement of multiple independent interactions arising from the same genomic region.

## Materials and Methods

The *T. californicus* individuals used for sequence analysis of *GOT2* and crosses were collected from intertidal rock pools at three sites in southern California, San Diego (SD, 32.7457°N, 117.2550°W, San Diego County, CA), La Jolla (LJS, 32.8434°N, 117.2808°W, San Diego County, CA), and Abalone Cove (AB, 33.7377°N, 118.3753°W, Los Angeles County, CA), three sites in central/northern California, San Simeon (SS, 35.5816°N, 121.1212°W, San Luis Obispo County, CA), Santa Cruz (SCN, 36.9495°N, 122.0470°W, Santa Cruz County, CA), and Bodega Head (BHB, 38.3051°N, 123.0654°W, Sonoma County, CA), and one site near Vancouver, Canada (BC, 49.3381°N, 123.2502°W, West Vancouver, BC). For the populations involved in the crosses, the SD and LJS populations are 10 percent divergent from one another in cytochrome B (*CYTB*), while each are 20 percent divergent from the AB population [17]. Note that Burton [23] used the LJP population for population crosses instead of the LJS population used in this study, but these are similar genetically and geographically proximate [17]. The copepods were maintained in mass culture in artificial seawater (Instant Ocean, Aquarium Systems Inc.) in 400-ml beakers at 20° with a 12∶12 light:dark (L:D) photoperiod. Cultures were maintained at a concentration of 35 parts per thousand seawater and fed with commercial flake fish food although copepods were also able to consume natural algal growth in these cultures. New cultures were established by sampling approximately 100 copepods from across a set of previous cultures to help maintain large overall population sizes.

### Identification and characterization of *GOT2* from *T. californicus*

RNA from SD copepods was isolated using the TRI reagent RNA isolation procedure in accordance with the supplier's protocols (Sigma Chemical, Saint Louis, MO). The kit Generacer (Invitrogen, Carlsbad, CA) was used for the 3′ RACE procedure with degenerate primers designed to match conserved regions of GOT-type proteins from a diverse set of animal taxa. For the identification of the *GOT2* gene a single primer (GOTall_deg.f, 5′-TGYGCNCAYAAYCCNACNGGNGT-3′) was used in conjunction with two kit supplied 3′ Generacer primers to generate PCR products in sequential nested PCR reactions. A set of PCR products were amplified by this procedure and these were cloned using the TOPO TA cloning kit (Invitrogen) and the resultant plasmids were sequenced. This procedure identified the 3′ end of one gene that appears to encode a GOT2 homolog and several others encoding GOT1 homologs (work is on-going to further characterize these apparent GOT1 genes). The 5′-end of the mRNA was obtained for the *GOT2* gene using the Generacer kit with newly designed *Tigriopus*-specific primers.

To obtain genomic sequences from individual copepods, DNA was first extracted from single copepods using a proteinase-K cell-lysis method [29]. DNA was prepared and sequences obtained from four individuals from each of the LJS, SS, SCN, BHB, and BC populations and five individuals each for the SD and AB populations. PCR products for *GOT2* were generated by using the two *T. californicus-*specific primers, GOT2ex1.f, 5′-TCTTGGTGGTCTGGCGTGGAAATG-3′ and GOT2stop.r, 5′GTCTTCTTATTTAGTGACAGCGTG-3′ that amplified a product of about 1400 bp. PCR products were sequenced directly (to avoid cloning artifacts) and included sequences from both strands for most individuals. Heterozygous sites were identified by visual inspection of sequences but the phase of multiple polymorphisms in the same individual was not unambiguously determined. All sequence editing was performed using Sequencer v4.8 software (Genecodes, Ann Arbor, Michigan). Prediction of mitochondrial targeting peptide sequence and cleavage site was done using Mitoprot v1.101 [30]. For analyses of sequence divergence and polymorphism in *GOT2* for *T. californicus*, nucleotide sequences were aligned and a nexus file was constructed with an alignment of sequences from individual copepods. Polymorphism and divergence analyses were done using the program DnaSP v5.1 [31]. Sequences are available in File S1 and have been submitted to Genbank with the accession numbers JF274264-JF274282.

Tests for positive selection on *GOT2* were conducted using the program PAML to identify if any lineages had significantly elevated d_N_/d_S_ ratios. A single sequence was selected from each population to use for phylogenetic analyses and PAML selection analyses. The program package PAML 4.3 [32] was used to conduct d_N_/d_S_ ratio (ω)-type selection analyses on this set of sequences. For these analyses the following relationships among populations were used: [((SD, LJS)AB), SS, (SC(BH, BC))]. These relationships were those obtained from a broader analysis of *CYTB* [17] and were also consistent with the phylogenetic results for *GOT2* obtained in this study. For the analysis of variation across sites for ω, the codeml program was used to compare the neutral model (M1a model with ω = 0 and ω = 1 categories of sites) with the selection model (M2a model with the same two classes of sites as the M1a model with a third class of sites with ω≥0).

### Crosses and genotyping

To test for the existence of epistatic interactions between genome regions containing our targeted proteins, crosses were set up using copepods from the AB, LJS, and SD populations that had been maintained in the culturing conditions described previously for at least one year (multiple generations are likely to have occurred). Virgin females were obtained by separating clasped pairs [33] and these females were placed in dishes with males from the other population. Crosses were done in petri dishes with 15 males and 15 females, and two plates were set up for each of the crosses performed (ABf x SDm, ABf x LJSm, ABm x LJSf, SDm x LJSf, and SDf x LJSm). Parental female copepods were moved to new dishes when copepodids of the next generation were observed. Males were removed and discarded after mating because females mate only once in their lifetime and store sufficient sperm to produce multiple clutches. F_1_ male and female copepods were allowed to mate after copepods were mixed across the two replicate petri dishes for each cross type (to minimize the chance of inbreeding). This crossing design will have the effect of averaging over the genetic variation found within each population.

To obtain F_2_ progeny, sets of 20 mated F_1_ females were placed into a new petri dish and allowed to reproduce. For the ABf x SDm cross the mated F_1_ females were split into two sets, one set was reared at a constant 20° with a 12 h :12 h L:D daily cycle (20° constant) and another in a 20–28°, 12 h: 12 h daily cycling environment with a 12 h: 12 h L:D cycle (henceforth 28° cycling). These temperatures matched the moderate and high conditions used for competition assays in Willett [34] that revealed differences in fitness among populations. F_1_ females were transferred to new petri dishes when F_2_ copepodids were observed. F_2_ copepods were collected as both nauplii and adults as described in Willett [35]. Only F_2_ adults were collected from the 28° cycling cross, while both F_2_ adults and nauplii were collected from all 20° constant crosses. For the ABf x LJSm a second set of crosses was set up for the purpose of collecting F_2_ nauplii to check the results from the first set. The same procedures outlined above were used except that mated F_1_ females were placed into individual wells in a 24-well microtiter plate in 2 ml of seawater to keep better track of individual females.

Genotyping was performed on F_2_ individuals for three different genes, *ME1*, *ME2*, and *GOT2*, but only *ME2* was scored in the SD x LJS crosses. For the ABf x SDm crosses the three markers were scored from both the 28° cycling and 20° constant crosses with 241 males, 411 females, and 174 nauplii genotyped in the 20-constant cross and 355 males and 305 females genotyped in the 28° cycling cross. The genotyping for these genes was done using population-diagnostic nucleotide differences or insertion-deletion differences to design primers that amplified different length PCR products for different populations. Details on PCR-based genotyping can be found in Table S1 (see also Willett and Berkowitz [28]). Three electron transport system associated genes *CYC*, *CYC1*, and *RISP* were also genotyped in these hybrids of the ABf x SDm crosses and results are given in Table S2 but not discussed in detail in this paper as results do not add significantly to those obtained in previous studies [35], [36]. These three markers were, however, used in tests of two-locus epistatic interactions as explained later. For the ABf x LJSm cross, *ME1*, *ME2*, and *GOT2* were genotyped for 181 males, 295 females, and 93 nauplii. For the SD x LJS crosses only the *ME2* gene was genotyped with 175 males, 367 females, and 183 nauplii genotyped in the SDf x LJSm cross and 73 male, 209 female, and 93 nauplii genotyped in the SDm x LJSf cross. For *ME2* in the SD x LJS crosses a dCAPS marker was developed using dCAPS finder 2.0 [37] that relied on a Tru1I digestion of PCR amplified products to generate population-diagnostic length products that could be scored on agarose gels.

The genotypes of the markers were tested for homogeneity across the sexes in F_2_ hybrids and for deviations from Mendelian inheritance as described previously [35], [38]. Briefly, the genotypic ratios of F_2_ hybrids were tested for departures from homogeneity between sexes using contingency tests at each of the markers. Deviations from Mendelian 1∶2∶1 ratios were examined for the sexes separately and for the combined sample with a χ^2^ analysis. Deviations from Mendelian inheritance were also quantified using Haldane's [39] formula for computing relative viabilities which examines the deviation of each homozygous genotypic class from an expected 1∶2 ratio assuming that the viability of the heterozygous genotypic class is 1. To test for linkage or epistatic interactions between loci, two-locus comparisons of deviations from independence were performed. These tests examine the deviations from the expected numbers of each of the two-locus genotypic classes after adjusting for the deviations in genotypic ratios that are observed for each single locus [35].

## Results

### *GOT2* characterization and evolution

Phylogenetic analyses of the predicted amino acid sequence encoded by the *GOT2* gene from *T. californicus* places this protein with other mitochondrial-targeted aspartate transaminases (Figure S1). The *T. californicus* GOT2 protein is most closely related to a mitochondrial-targeted aspartate transaminase (ACO10233) found in another copepod species (they share 75 percent amino acid identity), and these fall within a group of arthropod mitochondrial-targeted aspartate transaminases proteins. For the *T. californicus* GOT2 protein the N-terminus is predicted to be a mitochondrial-targeting peptide that is cleaved after position 44 with a probability of 97.7 percent (position in GOT homolog amino acid sequence file, see File S2). This *GOT2* locus is likely to be the same used previously in allozyme analyses in *T. californicus* [23], an enzyme that was shown to have aspartate transaminase enzymatic activity in the mitochondrial fraction of copepod homogenates [40].

There was a significant amount of inter-population divergence in *GOT2* sequences in comparisons among seven different *T. californicus* populations and this divergence includes a large number of amino-acid changing mutations (Sequence alignment available in File S1). There are eleven different nonsynonymous mutations that are fixed among populations with nine of these occurring in the southern California population group including the SD, LJS, and AB populations (Table 1). There is no evidence from PAML analyses that nonsynonymous changes have been driven by positive selection with no difference between a nearly neutral sites model including two rates (ω = 0.019 and ω = 1, with a corresponding 92% and 8% of sites falling into these two classes) and a model allowing a third rate (this third class of sites has an estimated ω = 1 as well). Exploration of branch/site models that allow evolutionary rates to vary among both sites and branches suggest that there is a class of sites evolving more rapidly in the group of SD, LJS, and AB; however, the estimated ω rate on these branches does not exceed one (results not shown). In contrast to the sizable number of nonsynonymous (11 fixed differences) and synonymous (30 fixed differences) changes among *T. californicus* populations for the *GOT2* gene, there are no nonsynonymous polymorphisms segregating within any of these populations (there are 11 synonymous polymorphisms); however, McDonald/Kreitman tests [41] provide no strong support for selection driving the fixation of nonsynonymous changes within populations with no single population showing a significant deviation from neutrality (data not shown). Examinations of the polymorphism frequency spectra within each of these populations also do not uncover any deviations consistent with a recent selective sweep (Table 1). Therefore, although the GOT2 protein appears to be diverging relatively rapidly between populations, there is no evidence from these sequence-based tests for significant deviations from neutrality at this locus in these populations.

**Table 1:** Polymorphism and divergence in *GOT2* in *T. californicus* populations.

Population	alleles	Fix NS	Fix Syn	Fix Intron	Poly NS	Poly Syn	π_SYN_	Poly Intron	π_NC_	Taj D	F-L D	F-L F	F-W H
SD	10	2	3	1	0	1	0.002	0	0.0012	1.30	0.74	1.00	0.18
LJS	8	1	0	1	0	4	0.0045	1 (+1 del)	0.0038	−0.50	−0.80	−0.86	1.14
AB	10	3	9	3 (+1 del)	0	1	0.0008	0	0.00045	−1.11	−1.35	−1.50	0.18
SS	8	1	3	7	0	0	0	1 (+1 ins)	0.0013	1.44	0.79	1.07	0
SCN	8	0	2	3 (+1 ins)	0	4	0.008	6	0.0119	1.83	1.69*	1.75*	1.43
BHB	8	1	0	0	0	1	0.0017	3	0.0027	−1.03	−0.40	−0.65	−0.79
BC	8	0	1	1	0	0	0	0	0	0	0	0	0
SD/LJS		3	5	5 (+1 del)									
SD/LJS/AB		0	3	2									
BHB/BC		0	0	1									
SCN/BHB/BC		0	4	0									

The number of changes inferred via parsimony for nonsynonymous (Fix NS), synonymous (Fix Syn), and intron (Fix Intron) nucleotide changes are given for the branch leading to each population. Changes on branches leading to groups of related populations are also given (e.g. SD/LJS changes would be changes shared by these two populations). Insertion (ins) and deletion (del) events are also given for the intron sequences. Numbers of polymorphisms (Poly) within populations are given for the same categories as divergence, while average pairwise genetic differences within populations are given for synonymous sites alone (^1^_SYN_) or synonymous plus intron sites (^1^_NC_). Tajima's D (Taj D [52]), Fu and Li's D (F-L D [53]) and F (F-L-F) with outgroups, and Fay and Wu's H (F-W H [54]) are shown. Outgroups for Fu and Li D and F and Fay and Wu's H were the AB (for LJS and SD), SD (for AB), and BC (for SCN and BHB) populations. Finally, significance of Fu and Li's D and F are denoted with an * = P<0.05 (no significant measures were found for Tajima's D or Fay and Wu's H).

### Departures from Mendelian inheritance in F_2_ hybrids

**Figure 2:**
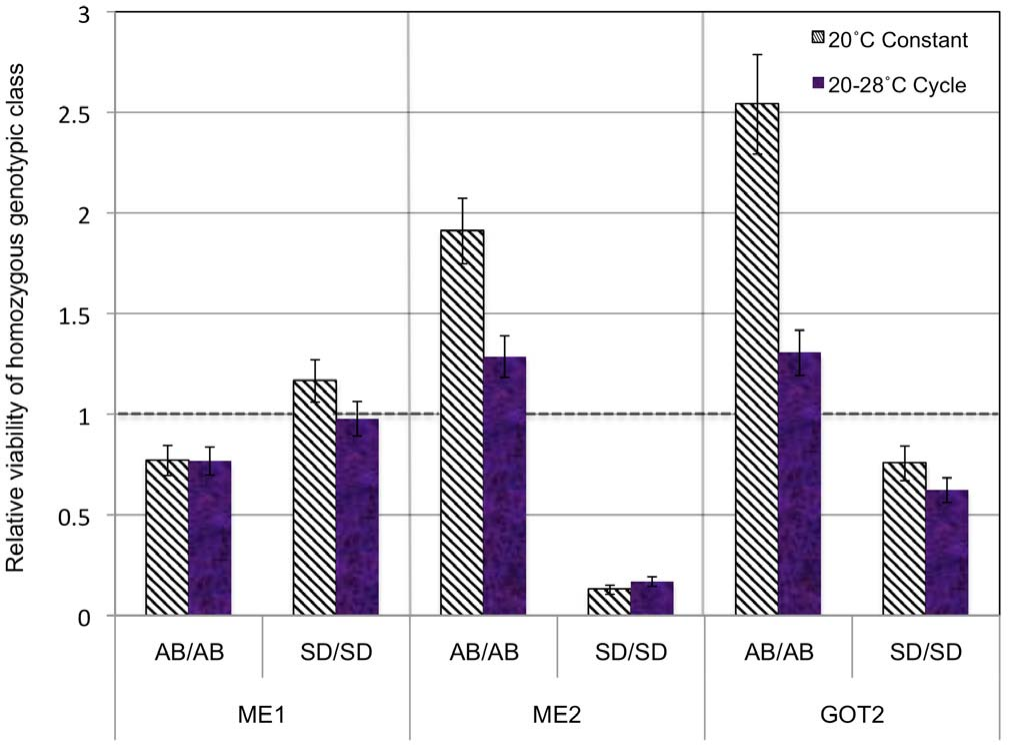
Relative viability of the *ME1*, *ME2*, and *GOT2* markers in ABf x SDm cross. The results are given for F_2_ adults hybrids for each of the three genes under two different sets of environmental conditions from crosses of the AB and SD populations of the copepod *T. californicus*. The 20° constant and 28° cycling crosses are indicated with hatched and solid bars respectively. The relative viability of the two homozygous genotypic classes in comparison to the heterozygous genotypic class is given with standard deviations indicated by error bars. A value of one would indicate that the homozygous class had the expected number relative to the heterozygous class, i.e. a 1∶2 ratio, while higher or lower values would represent more or fewer than expected respectively. The homozygous genotypic class is indicated by AB/AB or SD/SD. Although there were significant differences in genotypic ratios between males and females, these stemmed from differences in the magnitude and not direction of deviations (with females having larger deviations). Full numerical results are available in Table S2.

**Figure 3:**
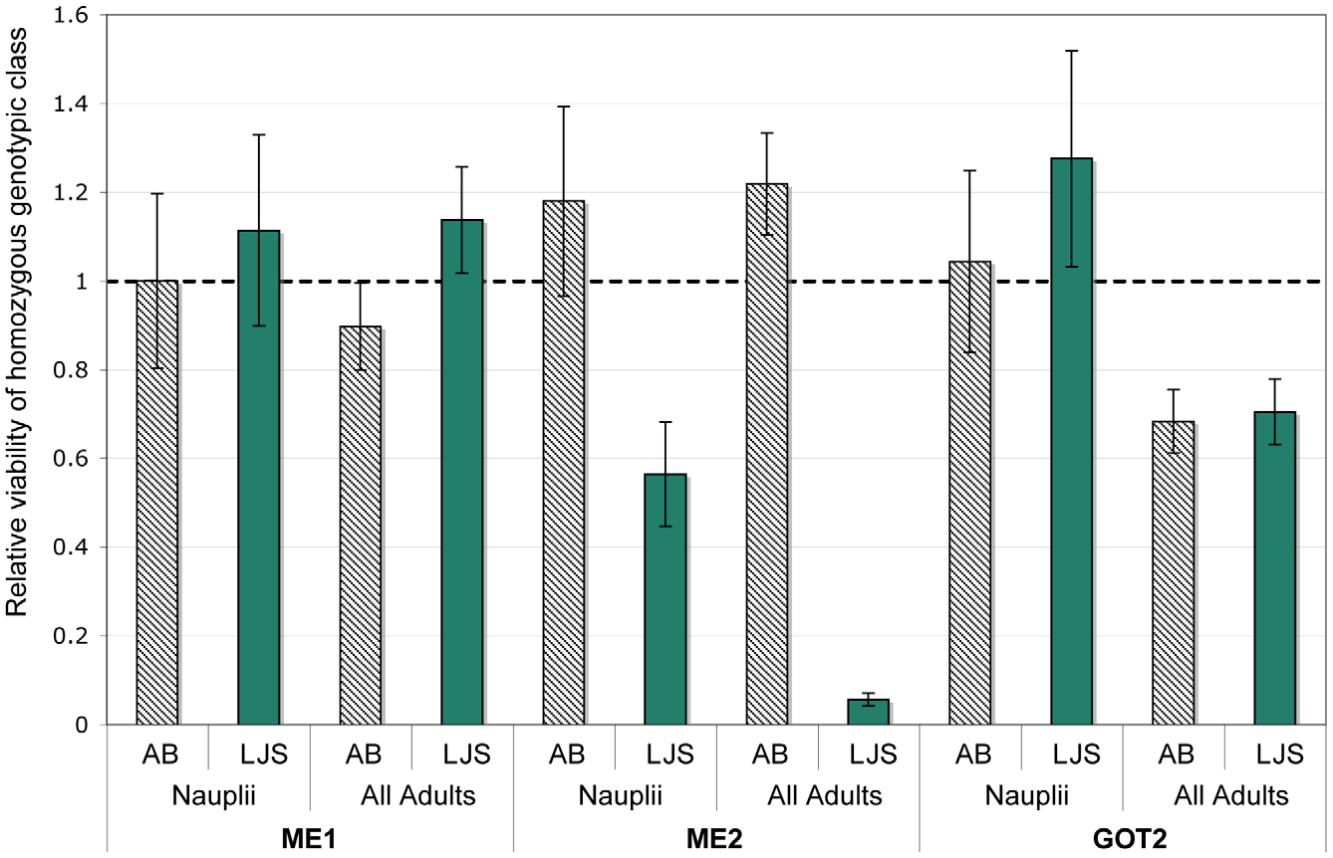
Relative viability of the *ME1*, *ME2*, and *GOT2* markers in the ABf x LJSm cross. Relative viability of each of the three markers in the *ME1*, *ME2*, and *GOT2* genes are shown from F_2_ adult hybrids from the cross of the AB and LJS populations of *T. californicus*. The relative viability of the two homozygous genotypic classes in comparison to the heterozygous genotypic class is given with standard deviations indicated by error bars as described for Figure 2. AB indicates the AB/AB homozygous genotypic class while LJS indicates the LJS/LJS genotypic class. Full numerical results including results from the reciprocal cross are available in Table S2.

**Figure 4:**
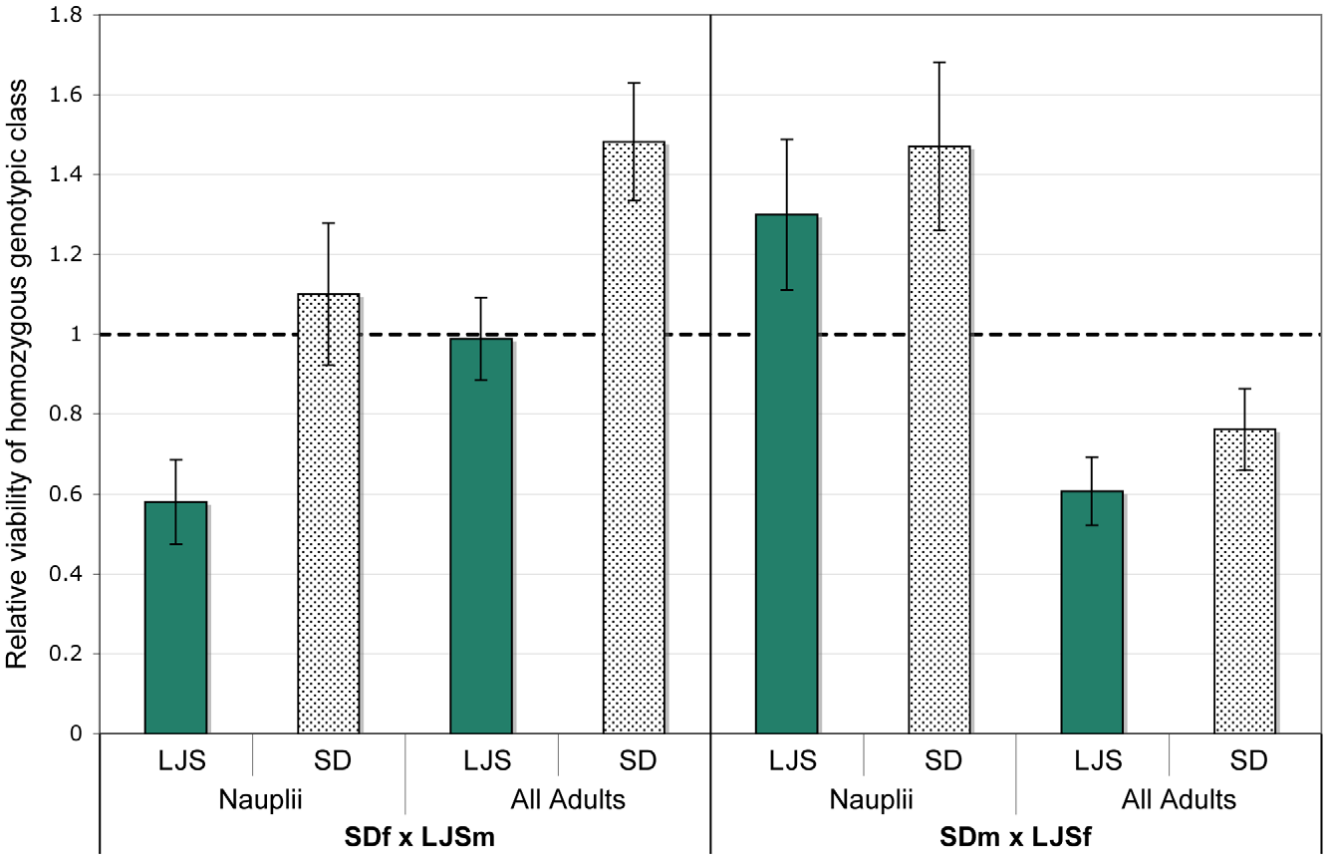
Relative viability at *ME2* for SD x LJS population crosses of *T. californicus*. The relative viability of the two homozygous genotypic classes in comparison to the heterozygous genotypic class is given for *ME2* in both adult and nauplii F_2_ hybrids from crosses of the SD and LJS populations. Relative viabilities and standard deviations are depicted as described for Figure 2. SD indicates the SD/SD homozygous genotypic class while LJS indicates the LJS/LJS genotypic class. Full numerical results are available in Table S2.

In F_2_ hybrids of population crosses of *T. californicus* there were significant departures from Mendelian expectations for each of the *ME1*, *ME2*, and *GOT2* marker's genotypic ratios in at least one cross (Table 2). In the ABf x SDm cross at 20° constant F_2_ adults but not nauplii depart substantially from Mendelian ratios for each of the three markers. The *ME2* and *GOT2* genes show significant deviations after corrections for multiple tests under both the 20° constant and 28° cycling rearing conditions for F_2_ adults. In these hybrids for the *ME2* locus, the SD/SD homozygous class has dramatically lowered viability for both the 20° constant and the 28°cycling conditions (Figure 2; Table S2). For the ABf x LJSm cross the lowest viabilities are seen for the LJS/LJS homozygous genotypic class at the *ME2* gene in F_2_ adults (Figure 3). It is then the closely related LJS and SD *ME2* homozygotes that have lowered viability in these crosses with the AB population (SD and LJS have only 4 silent fixed changes between them in *ME2* while SD and AB have 36 silent and 2 replacement fixed changes between them in *ME2*). The AB/AB genotypic class at *GOT2* showed a strikingly high relative viability in the ABf x SDm cross (particularly at 20° constant) but not in the ABf x LJSm cross where both genotypic classes have lowered viability. Only the *ME2* gene was genotyped for the SD x LJS crosses and there were significant deviations from Mendelian inheritance for F_2_ adults in the SDf x LJSm (Table 1). In this cross, the relative viability of the SD/SD homozygous genotypic class for *ME2* is higher than expected (Figure 4). The two reciprocal crosses differ significantly from one another for the SD and LJS crosses at *ME2* (χ^2^ value of 16.5, P = 0.0002 with two degrees of freedom). For each of the ABf x LJSm and LJS x SD crosses there are departures from Mendelian inheritance among F_2_ nauplii for *ME2* but these are not significant when corrected for multiple comparisons. Deviations in F_2_ nauplii for *ME2* were not consistent in a repeated cross of the ABf x LJSm population (Table S2). Nonetheless, the results in nauplii at least hint that there could be deviations from expected ratios in crosses involving the LJS population that are not seen for crosses involving the SD population.

**Table 2:** Single-locus tests for deviations from Mendelian ratios and between sexes.

Gene	χ^2^ naup	P-value	χ^2^ M-F	P-value	χ^2^ Female	P-value	χ^2^ Male	P-value	χ^2^ All adults	P-value
ABf x SDm 20° constant										
*ME1*	3.08	0.21	1.00	0.61					12.4	0.002*
*ME2*	1.26	0.53	8.97	**0.012**	76.2	<0.00001*	43.6	<0.00001*	246	<0.00001*
*GOT2*	1.59	0.45	13.0	0.0014*	161	<0.00001*	28.7	<0.00001*	175	<0.00001*
ABf x SDm 28°-cycle										
*ME1*			0.003	1.0					7.10	**0.029**
*ME2*			0.41	0.82					154	<0.00001*
*GOT2*			5.67	0.058					39.5	<0.00001*
ABf x LJSm										
*ME1*	0.40	0.82	1.4	0.49					3.38	0.18
*ME2*	8.50	**0.014**	1.3	0.51					140	<0.00001*
*GOT2*	1.84	0.40	3.46	0.18					15.3	0.0005*
SDf x LJSm										
*ME2*	8.62	**0.013**	0.15	0.93					19.5	0.00006*
LJSf x SDm										
*ME2*	8.33	**0.016**	1.96	0.36					10.8	**0.0045**

The χ^2^ values are given for test of departures of observed genotypic ratios at each locus from Mendelian inheritance or from contingency table analyses of differences in genotypic ratios between sexes (M-F). These tests are done for F_2_ nauplii (naup), adult males and females (when there was not a significant departure between the sexes only the combined results are shown), and all adults. P-values are given for each test with 2 degrees of freedom and values lower than P = 0.05 are shown in bold. An * indicates when a P-value exceeds the threshold for a sequential Bonferroni correction for this table (P = 0.0023). Full tabulations of results are given in Supplemental Table S3.

**Figure 5:**
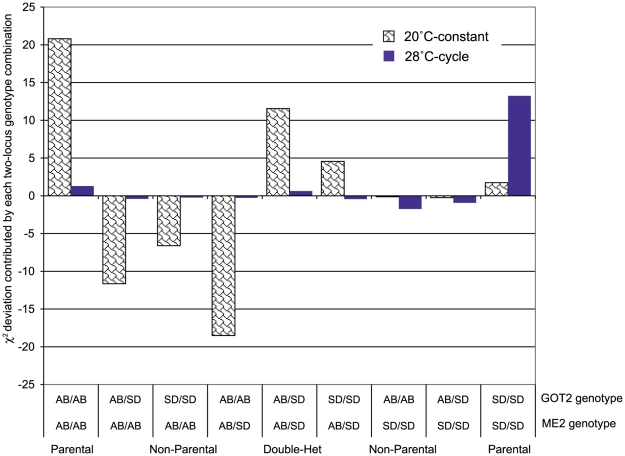
Two-locus comparison between *ME2* and *GOT2* from the cross of ABf x SDm. The relative contributions for the two-locus genotypic class combinations at *ME2* and *GOT2* to deviations from independence for F_2_ adult hybrids from the AB and SD populations of *T. californicus* are shown. The bars indicate the χ^2^ deviation contributed by each two-locus genotypic class for each of two temperature environments. A positive value indicates that more individuals in that class were observed than expected while a negative value indicates that less were observed than expected. The expected distributions under two different models of three-locus DM incompatibilities can be seen in Figure 6 with other two-locus models explored in Willett [42].

There are significant deviations from independence in two-locus tests between the *GOT2* and *ME2* loci in the ABf x SDm cross of *T. californicus* populations, potentially indicating that DM incompatibilities exist between these two regions of the genome in these hybrids (Table 3). These loci show significant departures from expectations in F_2_ hybrids after correcting for the effects of each locus alone in the adults from the ABf x SDm crosses but F_2_ nauplii from this cross do not show departures from independence between these two genes. The two-locus genotypic classes that contribute to this deviation differ substantially between the 20° constant and the 28° cycling environments in the ABf x SDm crosses implying that the temperature environment can alter the nature of interactions between these genome regions (Figure 5). No significant interactions between *ME2* and *GOT2* are seen in the ABf x LJSm cross in F_2_ adults nor in the ABm x LJSf cross; however, some questions arose in this ABm x LJSf dataset about possible contamination and these have been included here only for comparison purposes (Table S2 and S3). In the ABf x SDm crosses one other set of significant interactions between the loci examined in this study involved the *GOT2/RISP* combination where both F_2_ adults and nauplii show similar levels of skew, which combined with the pattern of missing two-locus genotypes suggests that *GOT2* and *RISP* are physically linked (Table S3).

**Table 3:** Test for two-locus deviations from independence in population crosses of *T. californicus*.

	*ME1/ME2*	*ME1/GOT2*	*ME2/GOT2*
ABf x SDm 20°			
Total adult χ^2^	6.63	4.74	75.8
Total P-value	0.15	0.32	<0.00001*
Nauplii χ^2^	1.52	4.44	1.85
Nauplii P-value	>0.5	0.35	>0.5
ABf x SDm 28°			
Total adult χ^2^	6.66	9.70	19.2
Total P-value	0.15	**0.046**	0.0007*
ABf x LJSm			
Total adult χ^2^	1.90	4.16	1.86
Total P-value	>0.5	0.38	>0.5
Nauplii χ^2^	6.71	3.48	1.43
Nauplii P-value	0.15	0.48	>0.5
ABm x LJSf			
Total adult χ^2^	2.52	1.26	1.80
Total P-value	>0.5	>0.5	>0.5
Nauplii χ^2^	7.07	5.39	7.07
Nauplii P-value	0.13	0.25	0.13

This table gives the χ^ 2^ deviations from expected two-locus numbers for pairs of loci (calculated by adjusting expected numbers by deviations seen for each single locus). For the ABf x SDm cross 20° is the 20° constant, while 28° is the 28°-cycle. A P-value has been calculated using 4 d.f. (expected two-locus numbers can be calculated from knowing the frequencies of two genotypic classes for each of the two loci). P-values in bold are lower than 0.05 while those exceeding the sequential Bonferroni correction value of P = 0.001 are denoted with an * (calculated for the entire dataset including comparisons to RISP, CYC, and CYC1; see Supplemental Table S3).

## Discussion

In this paper I have shown that a set of markers in genes that encode metabolic enzymes have dramatically altered patterns of genotypic viability in *T. californicus* F_2_ hybrids suggesting that these regions of the genome may be involved in DM incompatibilities. First, I will discuss the large departures from Mendelian inheritance found for the *ME2* and *GOT2* markers in a set of crosses of *T. californicus* populations. Second, I will focus on the departures from independence for two-locus interactions between *ME2* and *GOT2* which suggest that the regions of the genome marked by these loci are involved in DM incompatibilities and the identity of the deleteriously interacting genes may differ among populations. It is important to note that although in the discussion below I will sometimes refer to interactions involving these specific genes, this is for convenience and the interaction could stem instead from a factor or factors linked to either of these loci. In F_2_ hybrids the markers will be linked to fairly large chromosomal regions given the limited number of generations that recombination has had to break up associations.

### Single locus effects of *GOT2*, *ME2*, and *ME1*

The *GOT2* locus had large departures from Mendelian expectations in each of the crosses, but the nature of these deviations differed between the crosses (Table 2, Figure 2 and 3). At the 20° constant temperature in the ABf x SDm cross there is a striking excess of the AB-type homozygote at *GOT2* with a deficit of the SD-type homozygote. One potential explanation for the elevated relative viability of the AB homozygous genotypic could be that heterozygotes at the focal locus in a DM incompatibility are selected against due to interactions with other derived homozygous loci, which has the effect of increasing the measure of relative viability at that locus [42]. Another potential explanation could be that the AB homozygote is favored in hybrids perhaps due to a release from deleterious mutations that have previously been fixed at the *GOT2* locus in the SD population. In the ABf x LJSm cross a different pattern is apparent for *GOT2* with both of the homozygous classes having reduced fitness in comparison to the heterozygous class (Figure 3). F_2_ hybrid adults and not nauplii for *GOT2* in these crosses show significant departures from Mendelian inheritance suggesting that the potential DM incompatibilities are lowering the fitness of copepods during development after the first free-swimming life stage. The lower sample sizes of the nauplii versus adult F_2_ hybrids could hamper the power to detect deviations in the nauplii but are unlikely to explain the different patterns seen in the adults and nauplii in this case. The different patterns of deviation from Mendelian expectations for *GOT2* between the ABf x SDm crosses and the ABf x LJSm cross suggest that this region of the genome in these two populations may differ in the nature of its interactions with other loci, a topic I will discuss further later.

I also found striking departures from Mendelian inheritance for particular genotypic classes at the *ME2* locus in a hybrid genetic background. For crosses involving either population from the San Diego area (SD or LJS) with the AB population, the homozygous genotypic class from the San Diego area population is dramatically reduced in viability in F_2_ adults (Figures 2 and 3). In the ABf x SDm cross, altering the rearing temperature did not alter this pattern of selection. This pattern of strong selection against the San Diego-type homozygous genotypic class was also found by Burton [23] for AB x LJP reciprocal crosses performed under varying salinity rearing regimes and by Willett and Berkowitz [28] for AB x SD reciprocal crosses. The repeatability of this effect stands in contrast to the variability seen for a number of other markers examined in hybrids in *T. californicus* when reared under different environmental conditions (e.g. temperature [43]) or even when the same cross was repeated using the same environmental conditions [38].

For *ME2* there were deviations from Mendelian inheritance for crosses between the two more closely related San Diego area populations as well in F_2_ adults (SD and LJS; Figure 4). This could suggest that this region of the genome has further diverged between these two populations as well as from the AB population in ways that can impact hybrid fitness. There is a significant difference between the reciprocal crosses for the SD x LJS cross for LJS but the pattern of deviations is not consistent with a simple model of cyto-nuclear coadaptation. Cyto-nuclear coadaptation (or even maladaptation in some cases [26]) has been suggested as a possible explanation of differences between reciprocal crosses for other markers in crosses of *T. californicus* populations [36]. Hybrid breakdown is lowered in crosses of *T. californicus* populations with lowered genetic divergence [24] suggesting that loci that cause incompatibilities may be less numerous in these crosses, which makes this finding of deviation in the closely related SD x LJS crosses interesting. In contrast to the results seen for nauplii at other markers, *ME2* in crosses involving the LJS population showed some evidence (although not significant when corrected for multiple comparisons) for departures from Mendelian expectations. It is possible then that the deleterious interaction involving *ME2* alleles from the LJS population could act both early and later in development.

**Figure 6:**
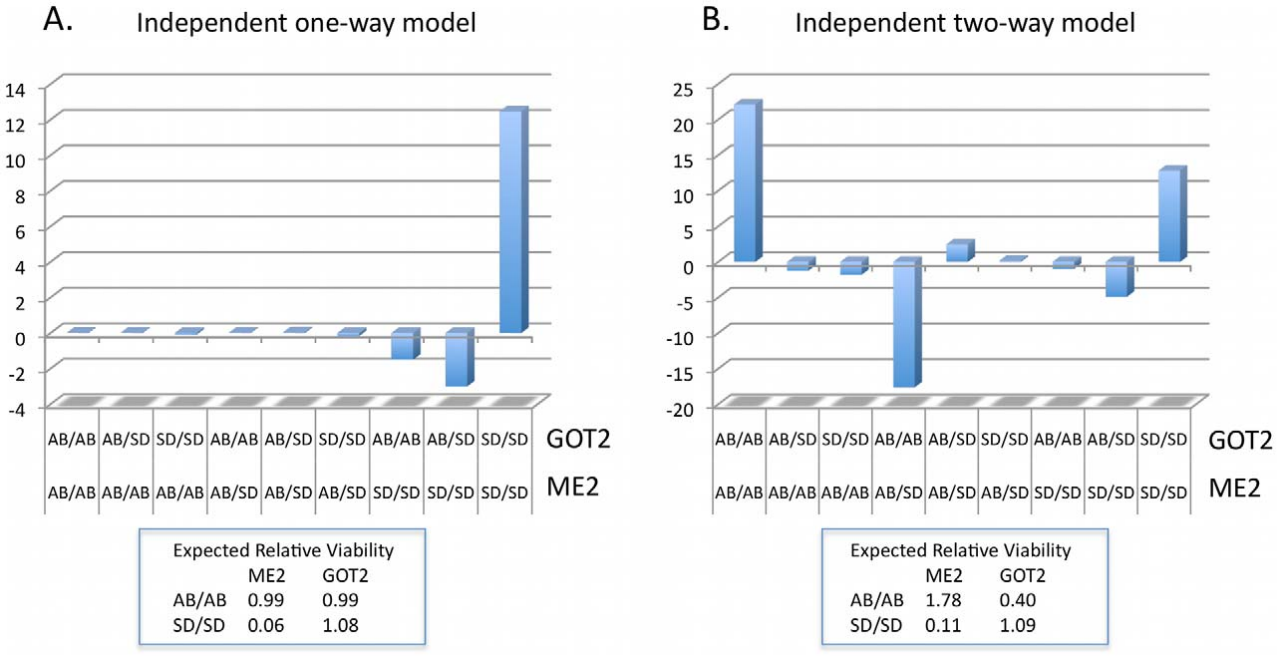
Expected two-locus deviations from independence for loci involved in three-way interactions. Models are constructed as in Willett [42] with the fitness of the three-locus combinations as given in Table 1 of that paper. Independent models involve two independent DM incompatibilities stemming from the separate interactions of two unlinked loci with the *ME2* locus. The one-way model (A.) has derived homozygotes at the *ME2* locus incompatible with heterozygotes at the other two loci (in addition to derived homozygotes at these two loci). The two-way model (B.) includes the addition of incompatibilities of heterozygotes at the *ME2* locus with derived homozygotes at the other two loci in addition to the interactions also found in the one-way case. The expected relative viabilities for the homozygous genotypic classes for each of the two focal loci are given the box below each plot (the third locus has the same pattern as *GOT2*).

The magnitude of the impacts on F_2_ hybrid fitness of the *ME2* locus, with relative viabilities of around ten percent in crosses of the SD and LJS populations with the AB population, suggest that this locus is likely to be involved in multiple independent DM incompatibilities. Simple models of F_2_ nuclear/nuclear incompatibilities will not lead to relative viabilities this low given that deleterious interactions have to involve at least one derived homozygous genotype at one locus interacting deleteriously with at least one derived allele at a second locus, otherwise there would be fitness problems in the F_1_ generation as well [42]. An interaction with mtDNA could produce a skew this extreme in relative viabilities but such an interaction is not supported for *ME2* by the reciprocal cross data where the SD or LJS homozygote class is still selected against despite matching the cytotype [23], [28]. The minimum relative viability that can be obtained in a two-locus interaction with derived homozygous genotypes at one locus and at least one derived allele at a second locus would be 25 percent. This could occur if complete inviability occurred when *ME2* was homozygous for the SD allele and the interacting locus was either heterozygous or homozygous for the AB allele (75 percent of SD/SD *ME2* homozygotes would be inviable). In contrast a homozygous/homozygous interaction could only reduce the relative viability by 25 percent to a relative viability of 75 percent. Involvement of loci in complex conspecific epistasis (i.e. derived alleles are needed at three or more loci) would only have the effect of decreasing the impact of any single locus in F_2_ hybrids. Therefore the magnitude of deviations seen from Mendelian inheritance at *ME2* across repeated crosses leads to the suggestion that this region of the genome may be involved in multiple independent DM incompatibilities in these population crosses (stemming from either the same locus or multiple loci linked to *ME2*), which could produce this magnitude of departure in relative viability. Models of multiple independent DM incompatibilities show that relative viability can be reduced to as low as 6 percent for the focal locus (Figure 6, [42]).

Unlike *ME2* and *GOT2* in the current dataset, the *ME1* marker shows roughly concordant patterns across crosses among these three populations showing that not all markers will behave differently in crosses involving SD or LJS. The *ME1* gene is predicted to encode another malic enzyme paralog in *T. californicus* that is substantially divergent from other characterized arthropod malic enzymes [28]. In contrast to the results discussed below for *ME2/GOT2*, there were no significant departures from two-locus independence involving *ME1*.

### Potential epistatic interactions between the *ME2* and *GOT2* genome regions

For *ME2* and *GOT2* in the ABf x SDm crosses at 20° constant and 28° cycling, there is a strong signal of non-independence between the two loci in F_2_ adults but not F_2_ nauplii (Table 3). In contrast to the *GOT2/RISP* combination where both nauplii and adults have large deviations consistent with linkage (Table S3), the pattern for *GOT2/ME2* suggests that in this cross there are epistatic interactions between these two regions of the genome that impact the fitness of hybrids, potentially a DM incompatibility. The two-locus combinations that show deviations between these two genes are different under the two temperature environments (Figure 5) implying some influence of extrinsic factors on this interaction. For either *ME2* or *GOT2* alone, the temperature environment impacted the magnitude of divergence from Mendelian ratios but not the direction of deviation (Figure 2). Environmental interactions with DM incompatibilities have been recognized as potentially important for the evolution of postzygotic reproductive isolation [44] and factors such as temperature have been found to both impact specific incompatibilities [43], [45], [46] and the expression of postzygotic reproductive isolation in general [44], [47], [48], [49].

The impacted two-locus classes in the *ME2/GOT2* interaction for the 28° cross are similar to those expected with an independent one-way model involving three loci, while for the 20° they more closely resemble the independent two-way model (Figure 6, [42]). Although these models can predict the large decreases in relative viability for the SD homozygotes at the *ME2* locus they cannot explain the increase in viability seen for the AB homozygotes at the *GOT2* locus in the 20°C cross. In fact, for the two-way model there would be a predicted decrease in the relative viability of the AB homozygote at *GOT2* (with little change in the one-way model). Overall these results suggest that there may be interactions between the *GOT2* and *ME2* genome regions causing DM incompatibilities but some discrepancies exist between the observed results and the predictions of two or three locus incompatibility models. The results from just these two models of three-locus DM incompatibilities from among a number of potential variants show that there can be a good deal of variation among the interacting loci in the patterns of deviations expected for both the individual loci and the two-locus interactions.

Strikingly, in the ABf x LJSm cross there is no evidence for epistatic interactions between *ME2* and *GOT2* in either the F_2_ adults or nauplii despite the strong deviations from expected Mendelian ratios seen at both *ME2* and *GOT2* loci in this cross. Burton [23] also found no evidence for epistatic interactions between the allozyme loci *ME* and *GOT2* for crosses between this pair of populations. He did find a non-significant trend for an interaction of these two genes in a cross between the LJ and SCN populations (a cross that was not done in the present study). The different pattern of interactions seen in the AB crossed with SD versus LJS populations suggests that the *ME2* locus (or a linked locus or loci) is involved with deleterious interactions with at least one different locus in the cross with SD (where one interacting locus appears to be *GOT2* or a nearby gene) then it is in the cross with LJS. If there are multiple independent incompatibilities stemming from the *ME2* region of the genome as suggested by the extremely low relative viabilities seen for the *ME2* locus, then this would suggest that the identity of the interacting partners may differ to some degree in crosses involving the SD versus the LJS population. These multiple independent incompatibilities need not involve only one factor in the ME2 region of the genome. In fact, in crosses of *Drosophila* species, finer resolution studies have sometimes decomposed one apparent locus into a number of closely linked loci that cause incompatibilities [9], [50], [51]. However, if the deleterious interactions do stem from a single factor in this region, then the pattern would fit the Kondrashov [10] model for complex interactions, with the same locus involved in multiple independent DM incompatibilities. Confirmation of whether there is a single factor in the *ME2* region of the genome involved in multiple independent incompatibilities will require further dissection of this region of the genome in these hybrid lineages.

### Conclusions

This exploration of the genetic basis of the early stages of postzygotic reproductive isolation has further confirmed the large impact of the *ME2* region of the genome. *ME2* displays repeatable, strong deviations from Mendelian inheritance that suggest this region of the genome is likely to be involved in multiple independent DM incompatibilities stemming from either one locus or multiple loci in this region of the genome. Crosses of the more closely related SD and LJS populations suggest that there may have been some divergence in the *ME2* genome region between these populations as well that could contribute to reproductive isolation. Also consistent with this idea is the observation that incompatibilities from this region may involve a factor from the *GOT2* region of the genome in crosses of AB with SD but not crosses of AB and LJS. These results would imply that at least one different partner (and likely also a further set of shared partners) may be involved in generating incompatibilities in these two different crosses with the same genomic region and illustrate that these interactions are likely to be complex in nature.

## Supporting Information

Figure S1 **Phylogenetic relationships between the predicted protein from the** ***GOT2*** **locus from** ***T. californicus*** **and aspartate transaminase homologs for other species.** Phylogeny was constructed for GOT2 amino acid sequences from homologs from a diverse set of taxa using MrBayes v3.1.2 (Ronquist and Huelsenbeck 2003) with 600 000 generations and a burn-in time of 150 000 generations. The analysis was conducted on the GOT amino acid alignment positions 47–498 (see nexus file in File S1). The best model was found to be one that used the WAG amino acid substitution model and adgamma rate variation between sites (gamma-distributed rates with autocorrelated rates across sites). A number of other models with different substitution modes (fixed, poisson, and equalin) and site rate variation (equal and gamma) were attempted but were found to fit the data more poorly by comparison of Bayes Factors. Values on branches are credibility values. The GOT2 sequence from *T. californicus* is circled while aspartate transaminase homologs from two other copepod species are underlined. Accession numbers are given for the aspartate transaminase homologs from other species with mitochondrial-targeted versus cytoplasmic proteins indicated as well. Ronquist F, Huelsenbeck JP (2003) MRBAYES 3: Bayesian phylogenetic inference under mixed models. Bioinformatics 19: 1572–1574.
(TIF)

Table S1 **Primer combinations used for genotyping F2 progeny of T. californicus population crosses.**
(DOCX)

Table S2 **Single locus genotypic ratios and tests for departures from Mendelian ratios.**
(DOCX)

Table S3 **Test for two-locus deviations from independence in population crosses of T. californicus.**
(DOCX)

File S1 **DNA sequences of GOT2 from T. californicus population samples.**
(DOC)

File S2 **Alignment of aspartate aminotransferase homologs with T. californicus GOT2 predicted protein.**
(DOC)
