# Supplementary figures and images for: Complex Deleterious Interactions Associated with Malic Enzyme May Contribute to Reproductive Isolation in the Copepod Tigriopus californicus
Source: PLoS One. 2011 Jun 22;6(6):e21177. doi: 10.1371/journal.pone.0021177 (PMC3120845; doi:10.1371/journal.pone.0021177)

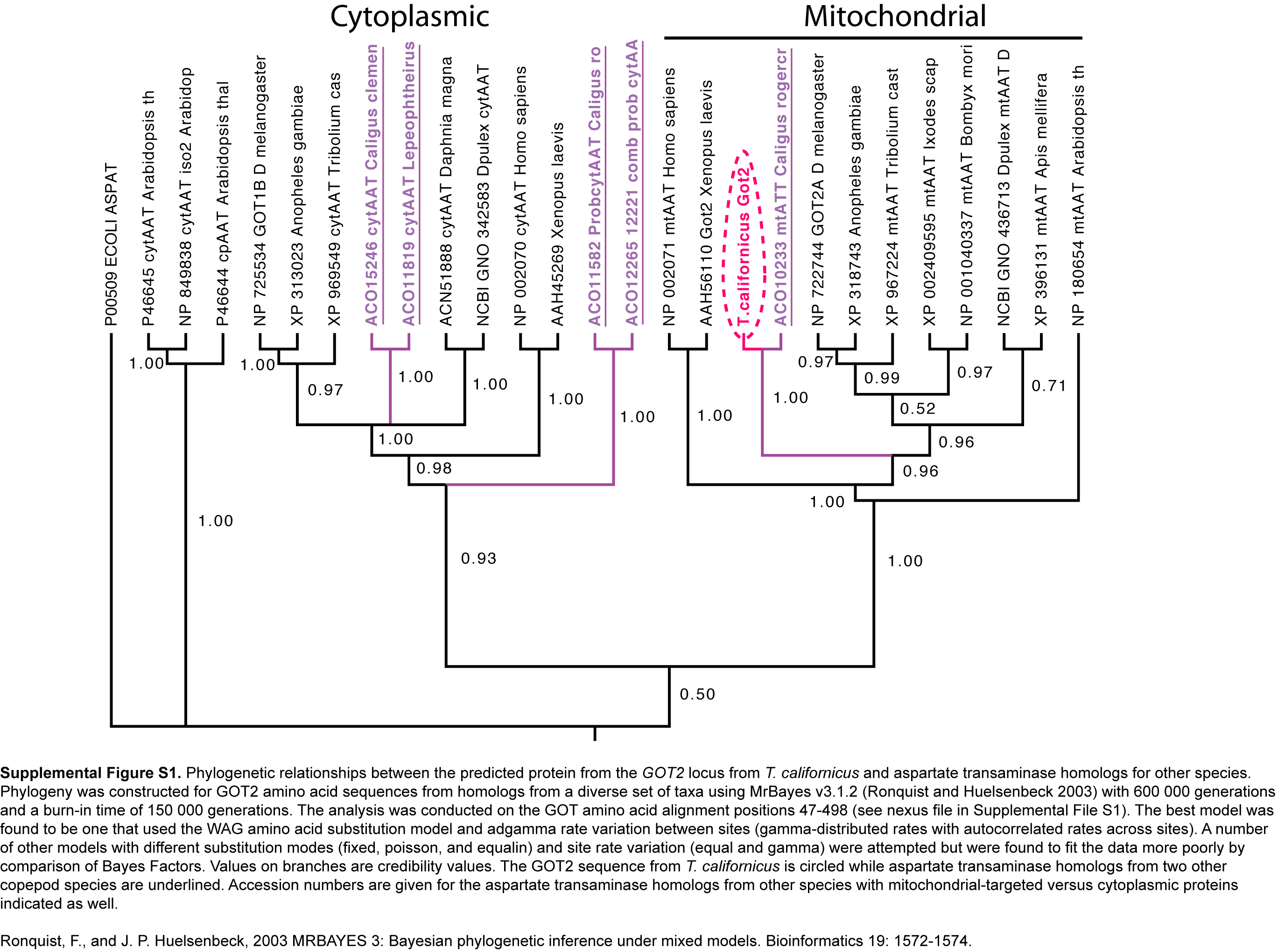

Supplement: Figure S1 — Phylogenetic relationships between the predicted protein from the GOT2 locus from T. californicus and aspartate transaminase homologs for other species. Phylogeny was constructed for GOT2 amino acid sequences from homologs from a diverse set of taxa using MrBayes v3.1.2 (Ronquist and Huelsenbeck 2003) with 600 000 generations and a burn-in time of 150 000 generations. The analysis was conducted on the GOT amino acid alignment positions 47–498 (see nexus file in File S1). The best model was found to be one that used the WAG amino acid substitution model and adgamma rate variation between sites (gamma-distributed rates with autocorrelated rates across sites). A number of other models with different substitution modes (fixed, poisson, and equalin) and site rate variation (equal and gamma) were attempted but were found to fit the data more poorly by comparison of Bayes Factors. Values on branches are credibility values. The GOT2 sequence from T. californicus is circled while aspartate transaminase homologs from two other copepod species are underlined. Accession numbers are given for the aspartate transaminase homologs from other species with mitochondrial-targeted versus cytoplasmic proteins indicated as well. Ronquist F, Huelsenbeck JP (2003) MRBAYES 3: Bayesian phylogenetic inference under mixed models. Bioinformatics 19: 1572–1574. (TIF) [file pone.0021177.s001.tif]
