# Supplementary material for: Complex Deleterious Interactions Associated with Malic Enzyme May Contribute to Reproductive Isolation in the Copepod Tigriopus californicus
Source: PLoS One. 2011 Jun 22;6(6):e21177. doi: 10.1371/journal.pone.0021177 (PMC3120845; doi:10.1371/journal.pone.0021177)
Supplement: Table S1 — Primer combinations used for genotyping F2 progeny of T. californicus population crosses. (DOCX) [file pone.0021177.s002.docx]

**Table S1.**

**Primer combinations used for genotyping F_2_ progeny of *T. californicus* population crosses**

| Gene | Cross- primer specificity | Primer Name | Sequence (5’ to 3’) | Annealing Temp. |
| --- | --- | --- | --- | --- |
| *GOT2* | AB x SD or LJS |  |  |  |
|  | Conserved AB/SD/LJS | GOT2_cons.fwd | GAGGCCTTCAAAAAGGATACTAATCC | 50˚C |
|  | Specific AB | GOT2_ab.rev | GAAACAATCTCATTTCCTGAAGATGA |  |
|  | Specific SD/LJS | GOT2_sd.rev | TTTTCAGGGATCCGGCTTATG |  |
| *ME1* | AB x SD or LJS |  |  |  |
|  | Conserved AB/SD/LJS | MEpro1con.f | GGAATGTACTTTTCTCACGGAGATCG | 54˚C |
|  | Specific AB | ME1_ab.r | CTAGAGCATCCTACGCATTCC |  |
|  | Specific SD/LJS | ME1_SD.r | ATATCAAGAACCTGTGCCACAC |  |
| *ME2* | AB x SD or LJS |  |  |  |
|  | Conserved AB/SD/LJS | MEprot2in_5.f | GTTCAACCCATTCCAACATGAGTTACA | 54˚C |
|  | Specific AB | ME2ab_3.r | TCTTTTTACCAACAGTCTTCATATCCTC |  |
|  | Specific SD/LJS | ME_SDnew.r | GCCCTAGGAAAGGAAACA |  |
|  | SD x LJS |  |  |  |
|  | Conserved SD/LJS | MEprot2.5.f | AATCACACCAACAATCGGGTCCAGA | 54˚C |
|  | Conserved SD/LJS | ME2_MseI_dcap.r | CAGTCTTCATATCGTTCCAAGTCAATAA |  |
| *RISP* | ABf x SDm |  |  |  |
|  | Conserved AB/SD | RISPcon.f2 | AAAGGCTGTGCCAGGTAAGTGAC | 58˚C |
|  | Conserved AB/SD | RISPcon.r2 | CATTGATTGTTCCGGACCAAAAACTC |  |
| *CYC1* | ABf x SDm |  |  |  |
|  | Conserved AB/SD | CYC1 ex3.con.f | GACTCGTTTGACCATGCTTCCATTCG | 58˚C |
|  | Conserved AB/SD | CYC1 ex4 con.r | CGGCATGGGTAGATAGTCGGTCAAT |  |
| *CYC* | ABf x SDm |  |  |  |
|  | Specific AB | ccAB.f | CCGACAGACGGGCAAGGCCTCT | 58˚C |
|  | Specific SD | ccSD.f | CCACAAAATGCCCCTCGCTCG |  |
|  | Conserved AB/SD | Cyt 14.r | GGAATGTACTTTTTGGGGTTCG |  |

Primers listed above for a pair of populations were combined into a single PCR reaction to generate population-specific sized fragments. PCR reactions for the crosses above were done by combining the primers listed above using the specified annealing temperature and otherwise standard PCR conditions. For all crosses/genes other than the ME2 gene in the SD x LJS crosses these PCR reactions generate a population-specific PCR product length that can be scored on an agarose gel. For the SD x LJS cross ME2 gene, the PCR product was subsequently digested with the Tru1I (or MseI) restriction enzyme to generate a population-specific PCR product. Data from RISP, CYC1, and CYC are presented in Supplemental Table S3 but not discussed in paper.
