# Supplementary material for: Complex Deleterious Interactions Associated with Malic Enzyme May Contribute to Reproductive Isolation in the Copepod Tigriopus californicus
Source: PLoS One. 2011 Jun 22;6(6):e21177. doi: 10.1371/journal.pone.0021177 (PMC3120845; doi:10.1371/journal.pone.0021177)
Supplement: Table S2 — Single locus genotypic ratios and tests for departures from Mendelian ratios. (DOCX) [file pone.0021177.s003.docx]

**Table S2**

**Single locus genotypic ratios and tests for departures from Mendelian ratios**

| Cross/  Progeny type | Gene/  Genotype | observed # | χ^2^ | P-value | M-F test^1^ | Cross/  Progeny type | Gene/Genotype | observed # | χ^2^ | P-value | M-F test^1^ | Recip. Cross^2^ |
| --- | --- | --- | --- | --- | --- | --- | --- | --- | --- | --- | --- | --- |
| **ABfxSDm 20˚constant** |  |  |  |  |  | **ABfxSDm 28˚cycling** |  |  |  |  |  |  |
|  | **ME1** |  |  |  |  |  | **ME1** |  |  |  |  |  |
| all female | AB/AB | 72 |  |  |  | all female | AB/AB | 62 |  |  |  |  |
|  | AB/SD | 181 |  |  |  |  | AB/SD | 162 |  |  |  |  |
|  | SD/SD | 114 |  |  |  |  | SD/SD | 79 |  |  |  |  |
|  | sum | 367 | 9.68 | 0.0079 |  |  | sum | 303 | 3.36 | 0.19 |  |  |
| all male | AB/AB | 47 |  |  |  | all male | AB/AB | 73 |  |  |  |  |
|  | AB/SD | 127 |  |  |  |  | AB/SD | 189 |  |  |  |  |
|  | SD/SD | 66 |  |  |  |  | SD/SD | 93 |  |  |  |  |
|  | sum | 240 | 3.83 | 0.15 |  |  | sum | 355 | 3.74 | 0.15 |  |  |
| nauplii^3^ | AB/AB | 36 |  |  |  |  |  |  |  |  |  |  |
|  | AB/SD | 84 |  |  |  |  |  |  |  |  |  |  |
|  | SD/SD | 29 |  |  |  |  |  |  |  |  |  |  |
|  | sum | 149 | 3.08 | 0.21 |  |  |  |  |  |  |  |  |
| all adults | AB/AB | 119 |  |  |  | all adults | AB/AB | 135 |  |  |  |  |
|  | AB/SD | 308 |  |  |  |  | AB/SD | 351 |  |  |  |  |
|  | SD/SD | 180 |  |  |  |  | SD/SD | 172 |  |  |  |  |
|  | sum | 607 | 12.39 | 0.002 |  |  | sum | 658 | 7.10 | 0.029 |  |  |
|  |  |  |  |  |  |  |  |  |  |  |  |  |
|  | **ME2** |  |  |  |  |  | **ME2** |  |  |  |  |  |
| all female | AB/AB | 202 |  |  |  | all female | AB/AB | 112 |  |  |  |  |
|  | AB/SD | 175 |  |  |  |  | AB/SD | 177 |  |  |  |  |
|  | SD/SD | 11 |  |  |  |  | SD/SD | 13 |  |  |  |  |
|  | sum | 388 | 191.77 | <0.00001 | |  | sum | 302 | 73.86 | <0.00001 | |  |
| all male | AB/AB | 96 |  |  |  | all male | AB/AB | 133 |  |  |  |  |
|  | AB/SD | 136 |  |  |  |  | AB/SD | 203 |  |  |  |  |
|  | SD/SD | 9 |  |  |  |  | SD/SD | 19 |  |  |  |  |
|  | sum | 241 | 66.80 | <0.00001 |  |  | sum | 355 | 80.54 | <0.00001 | |  |
| nauplii | AB/AB | 34 |  |  |  |  |  |  |  |  |  |  |
|  | AB/SD | 76 |  |  |  |  |  |  |  |  |  |  |
|  | SD/SD | 30 |  |  |  |  |  |  |  |  |  |  |
|  | sum | 140 | 1.26 | 0.53 |  |  |  |  |  |  |  |  |
| all adults | AB/AB | 298 |  |  |  | all adults | AB/AB | 245 |  |  |  |  |
|  | AB/SD | 311 |  |  |  |  | AB/SD | 380 |  |  |  |  |
|  | SD/SD | 20 |  |  |  |  | SD/SD | 32 |  |  |  |  |
|  | sum | 629 | 245.81 | <0.00001 | |  | sum | 657 | 154.25 | <0.00001 | |  |

**Table S2 (cont.)**

| Cross/  Progeny type | Gene/  Genotype | observed # | χ^2^ | P-value | M-F test^1^ | Cross/  Progeny type | Gene/Genotype | observed # | χ^2^ | P-value | M-F test | Recip. Cross |
| --- | --- | --- | --- | --- | --- | --- | --- | --- | --- | --- | --- | --- |
| ABfxSDm 20˚constant (cont.) | **GOT2** |  |  |  |  | ABfxSDm 28˚cycling (cont.) | **GOT2** |  |  |  |  |  |
| all female | AB/AB | 198 |  |  |  | all female | AB/AB | 110 |  |  |  |  |
|  | AB/SD | 135 |  |  |  |  | AB/SD | 157 |  |  |  |  |
|  | SD/SD | 41 |  |  |  |  | SD/SD | 38 |  |  |  |  |
|  | sum | 374 | 160.73 | <0.00001 | |  | sum | 305 | 34.25 | <0.00001 | |  |
| all male | AB/AB | 94 |  |  |  | all male | AB/AB | 106 |  |  |  |  |
|  | AB/SD | 94 |  |  |  |  | AB/SD | 173 |  |  |  |  |
|  | SD/SD | 46 |  |  |  |  | SD/SD | 65 |  |  |  |  |
|  | sum | 234 | 28.74 | <0.00001 | |  | sum | 344 | 9.784 | 0.0075 |  |  |
| nauplii | AB/AB | 33 |  |  |  |  |  |  |  |  |  |  |
|  | AB/SD | 76 |  |  |  |  |  |  |  |  |  |  |
|  | SD/SD | 44 |  |  |  |  |  |  |  |  |  |  |
|  | sum | 153 | 1.59 | 0.45 |  |  |  |  |  |  |  |  |
| all adults | AB/AB | 292 |  |  |  | all adults | AB/AB | 216 |  |  |  |  |
|  | AB/SD | 229 |  |  |  |  | AB/SD | 330 |  |  |  |  |
|  | SD/SD | 87 |  |  |  |  | SD/SD | 103 |  |  |  |  |
|  | sum | 608 | 175.25 | <0.00001 | |  | sum | 649 | 39.53 | <0.00001 | |  |
|  |  |  |  |  |  |  |  |  |  |  |  |  |
|  | **RISP** |  |  |  |  |  | **RISP** |  |  |  |  |  |
| all female | AB/AB | 102 |  |  |  | all female | AB/AB | 84 |  |  |  |  |
|  | AB/SD | 204 |  |  |  |  | AB/SD | 157 |  |  |  |  |
|  | SD/SD | 81 |  |  |  |  | SD/SD | 46 |  |  |  |  |
|  | sum | 387 | 3.42 | 0.18 | χ^2^ =2.93 |  | sum | 287 | 12.60 | 0.0024 |  | χ^2^ =11.8 |
| all male | AB/AB | 49 |  |  | P=0.23 | all male | AB/AB | 80 |  |  |  | P=0.003 |
|  | AB/SD | 122 |  |  |  |  | AB/SD | 178 |  |  |  |  |
|  | SD/SD | 59 |  |  |  |  | SD/SD | 95 |  |  |  |  |
|  | sum | 230 | 1.72 | 0.42 |  |  | sum | 353 | 1.30 | 0.52 |  |  |
| nauplii | AB/AB | 34 |  |  |  |  |  |  |  |  |  |  |
|  | AB/SD | 91 |  |  |  |  |  |  |  |  |  |  |
|  | SD/SD | 39 |  |  |  |  |  |  |  |  |  |  |
|  | sum | 164 | 2.28 | 0.32 |  |  |  |  |  |  |  |  |
| all adults | AB/AB | 151 |  |  |  | all adults | AB/AB | 164 |  |  |  |  |
|  | AB/SD | 326 |  |  |  |  | AB/SD | 335 |  |  |  |  |
|  | SD/SD | 140 |  |  |  |  | SD/SD | 141 |  |  |  |  |
|  | sum | 617 | 2.38 | 0.3 |  |  | sum | 640 | 3.06 | 0.22 |  |  |

**Table S2 (cont.)**

| Cross/  Progeny type | Gene/  Genotype | observed # | χ^2^ | P-value | M-F test^1^ | Cross/  Progeny type | Gene/Genotype | observed # | χ^2^ | P-value | M-F test | Recip. Cross |
| --- | --- | --- | --- | --- | --- | --- | --- | --- | --- | --- | --- | --- |
| ABfxSDm 20˚constant (cont.) | **CYC1** |  |  |  |  | ABfxSDm 28˚cycling (cont.) | **CYC1** |  |  |  |  |  |
| all female | AB/AB | 143 |  |  |  | all female | AB/AB | 73 |  |  |  |  |
|  | AB/SD | 209 |  |  |  |  | AB/SD | 167 |  |  |  |  |
|  | SD/SD | 59 |  |  |  |  | SD/SD | 65 |  |  |  |  |
|  | sum | 411 | 34.45 | <0.00001 | |  | sum | 305 | 3.18 | 0.21 |  |  |
| all male | AB/AB | 47 |  |  | χ^2^ =22.1 | all male | AB/AB | 78 |  |  | χ^2^ =0.36 |  |
|  | AB/SD | 134 |  |  | P=0.00002 |  | AB/SD | 197 |  |  | P=0.84 |  |
|  | SD/SD | 60 |  |  |  |  | SD/SD | 79 |  |  |  |  |
|  | sum | 241 | 4.43 | 0.11 |  |  | sum | 354 | 4.53 | 0.104 |  |  |
| nauplii | AB/AB | 46 |  |  |  |  |  |  |  |  |  |  |
|  | AB/SD | 81 |  |  |  |  |  |  |  |  |  |  |
|  | SD/SD | 40 |  |  |  |  |  |  |  |  |  |  |
|  | sum | 167 | 0.58 | 0.75 |  |  |  |  |  |  |  |  |
| all adults | AB/AB | 190 |  |  |  | all adults | AB/AB | 151 |  |  |  |  |
|  | AB/SD | 343 |  |  |  |  | AB/SD | 364 |  |  |  |  |
|  | SD/SD | 119 |  |  |  |  | SD/SD | 144 |  |  |  |  |
|  | sum | 652 | 17.24 | 0.0002 |  |  | sum | 659 | 7.37 | 0.025 |  |  |
|  |  |  |  |  |  |  |  |  |  |  |  |  |
|  | **CYC** |  |  |  |  |  | **CYC** |  |  |  |  |  |
| all female | AB/AB | 110 |  |  |  | all female | AB/AB | 73 |  |  |  |  |
|  | AB/SD | 233 |  |  |  |  | AB/SD | 148 |  |  |  |  |
|  | SD/SD | 64 |  |  |  |  | SD/SD | 78 |  |  |  |  |
|  | sum | 407 | 18.95 | 0.00008 | |  | sum | 299 | 0.20 | >0.95 |  |  |
| all male | AB/AB | 44 |  |  | χ^2^ =11.6 | all male | AB/AB | 44 |  |  | χ^2^ =14.9 |  |
|  | AB/SD | 136 |  |  | P=0.003 |  | AB/SD | 203 |  |  | P=0.0005 |  |
|  | SD/SD | 60 |  |  |  |  | SD/SD | 98 |  |  |  |  |
|  | sum | 240 | 6.40 | 0.041 |  |  | sum | 345 | 27.69 | <0.00001 | |  |
| nauplii | AB/AB | 48 |  |  |  |  |  |  |  |  |  |  |
|  | AB/SD | 88 |  |  |  |  |  |  |  |  |  |  |
|  | SD/SD | 38 |  |  |  |  |  |  |  |  |  |  |
|  | sum | 174 | 1.17 | 0.56 |  |  |  |  |  |  |  |  |
| all adults | AB/AB | 154 |  |  |  | all adults | AB/AB | 117 |  |  |  |  |
|  | AB/SD | 369 |  |  |  |  | AB/SD | 351 |  |  |  |  |
|  | SD/SD | 124 |  |  |  |  | SD/SD | 176 |  |  |  |  |
|  | sum | 647 | 15.58 | 0.0004 |  |  | sum | 644 | 16.03 | 0.0003 |  |  |

**Table S2 (cont.)**

| Cross/  Progeny type | Gene/  Genotype | observed # | χ^2^ | P-value | M-F test^1^ | Cross/  Progeny type | Gene/Genotype | observed # | χ^2^ | P-value | M-F test | Recip. Cross |
| --- | --- | --- | --- | --- | --- | --- | --- | --- | --- | --- | --- | --- |
| **ABf x LJSm** |  |  |  |  |  | **ABm x LJSf^4^** |  |  |  |  |  |  |
|  | **ME1** |  |  |  |  |  | **ME1** |  |  |  |  |  |
| all female | AB/AB | 61 |  |  |  | all female | AB/AB | 88 |  |  | p=0.68 |  |
|  | AB/LJS | 149 |  |  |  |  | AB/LJS | 212 |  |  | χ^2^ =0.78 |  |
|  | LJS/LJS | 85 |  |  |  |  | LJS/LJS | 120 |  |  |  |  |
|  | sum | 295 | 3.94 | 0.12 |  |  | sum | 420 | 4.91 | 0.086 | |  |
| all male | AB/AB | 45 |  |  |  | all male | AB/AB | 23 |  |  |  |  |
|  | AB/LJS | 84 |  |  |  |  | AB/LJS | 69 |  |  |  |  |
|  | LJS/LJS | 48 |  |  |  |  | LJS/LJS | 34 |  |  |  |  |
|  | sum | 177 | 0.56 | 0.76 |  |  | sum | 126 | 3.06 | 0.22 |  |  |
| 1st set nauplii | AB/AB | 22 |  |  |  | 1st set nauplii | AB/AB | 30 |  |  |  |  |
|  | AB/LJS | 47 |  |  |  |  | AB/LJS | 67 |  |  |  |  |
|  | LJS/LJS | 20 |  |  |  |  | LJS/LJS | 28 |  |  |  |  |
|  | sum | 89 | 0.37 | 0.83 |  |  | sum | 125 | 0.71 | 0.7 |  |  |
| 2nd set nauplii^5^ | AB/AB | 13 |  |  |  | 2nd set nauplii | AB/AB | 29 |  |  |  |  |
|  | AB/LJS | 22 |  |  |  |  | AB/LJS | 53 |  |  |  |  |
|  | LJS/LJS | 19 |  |  |  |  | LJS/LJS | 39 |  |  |  |  |
|  | sum | 54 | 3.19 | 0.2 |  |  | sum | 121 | 3.51 | 0.17 |  |  |
| total nauplii | AB/AB | 35 |  |  |  | total nauplii | AB/AB | 59 |  |  |  |  |
|  | AB/LJS | 69 |  |  |  |  | AB/LJS | 120 |  |  |  |  |
|  | LJS/LJS | 39 |  |  |  |  | LJS/LJS | 67 |  |  |  |  |
|  | sum | 143 | 0.40 | 0.82 |  |  | sum | 246 | 0.67 | 0.72 |  |  |
| all adults | AB/AB | 105 |  |  |  | all adults | AB/AB | 111 |  |  |  |  |
|  | AB/LJS | 233 |  |  |  |  | AB/LJS | 281 |  |  |  |  |
|  | LJS/LJS | 133 |  |  |  |  | LJS/LJS | 156 |  |  |  | χ^2^ =0.64 |
|  | sum | 471 | 3.38 | 0.18 |  |  | sum | 548 | 7.75 | 0.021 | | p=0.75 |
|  |  |  |  |  |  |  |  |  |  |  |  |  |
|  | **ME2** |  |  |  |  |  | **ME2** |  |  |  |  |  |
| all female | AB/AB | 101 |  |  |  | all female | AB/AB | 138 |  |  | p=0.43 |  |
|  | AB/LJS | 180 |  |  |  |  | AB/LJS | 271 |  |  | χ^2^ =1.71 |  |
|  | LJS/LJS | 6 |  |  |  |  | LJS/LJS | 15 |  |  |  |  |
|  | sum | 287 | 81.46 | <0.00001 | |  | sum | 424 | 104.20 | <0.00001 | |  |
| all male | AB/AB | 72 |  |  |  | all male | AB/AB | 49 |  |  |  |  |
|  | AB/LJS | 103 |  |  |  |  | AB/LJS | 76 |  |  |  |  |
|  | LJS/LJS | 3 |  |  |  |  | LJS/LJS | 3 |  |  |  |  |
|  | sum | 178 | 57.90 | <0.00001 | |  | sum | 128 | 37.56 | <0.00001 | |  |

**Table S2 (cont.)**

| Cross/  Progeny type | Gene/  Genotype | observed # | χ^2^ | P-value | M-F test^1^ | Cross/  Progeny type | Gene/Genotype | observed # | χ^2^ | P-value | M-F test | Recip. Cross |
| --- | --- | --- | --- | --- | --- | --- | --- | --- | --- | --- | --- | --- |
| ABf x LJSm (cont.) | ME2 (cont.) |  |  |  |  | ABmx LJSf (cont.) | ME2 (cont.) |  |  |  |  |  |
| 1st set nauplii | AB/AB | 28 |  |  |  | 1st set nauplii | AB/AB | 25 |  |  |  |  |
|  | AB/LJS | 56 |  |  |  |  | AB/LJS | 69 |  |  |  |  |
|  | LJS/LJS | 8 |  |  |  |  | LJS/LJS | 31 |  |  |  |  |
|  | sum | 92 | 13.04 | 0.0015 |  |  | sum | 125 | 1.93 | 1.93 |  |  |
| 2nd set nauplii | AB/AB | 18 |  |  |  | 2nd set nauplii | AB/AB | 42 |  |  |  |  |
|  | AB/LJS | 21 |  |  |  |  | AB/LJS | 56 |  |  |  |  |
|  | LJS/LJS | 14 |  |  |  |  | LJS/LJS | 20 |  |  |  |  |
|  | sum | 53 | 2.89 | 0.23 |  |  | sum | 118 | 8.51 | 0.014 |  |  |
| total nauplii | AB/AB | 46 |  |  |  | total nauplii | AB/AB | 67 |  |  |  |  |
|  | AB/LJS | 77 |  |  |  |  | AB/LJS | 125 |  |  |  |  |
|  | LJS/LJS | 22 |  |  |  |  | LJS/LJS | 51 |  |  |  |  |
|  | sum | 145 | 8.50 | 0.0142 |  |  | sum | 243 | 2.31 | 0.32 |  |  |
| all adults | AB/AB | 173 |  |  |  | all adults | AB/AB | 187 |  |  |  |  |
|  | AB/LJS | 283 |  |  |  |  | AB/LJS | 347 |  |  |  |  |
|  | LJS/LJS | 8 |  |  |  |  | LJS/LJS | 18 |  |  |  | χ^2^ =2.56 |
|  | sum | 464 | 139.77 | <0.00001 | |  | sum | 552 | 140.01 | <0.00001 | | p=0.28 |
|  |  |  |  |  |  |  |  |  |  |  |  |  |
| ABf x LJSm (cont.) | **GOT2** |  |  |  |  | ABmx LJSf (cont.) | **GOT2** |  |  |  |  |  |
| all female | AB/AB | 52 |  |  |  | all female | AB/AB | 102 |  |  | p=0.025 |  |
|  | AB/LJS | 176 |  |  |  |  | AB/LJS | 226 |  |  | χ^2^ =7.37 |  |
|  | LJS/LJS | 66 |  |  |  |  | LJS/LJS | 104 |  |  |  |  |
|  | sum | 294 | 12.78 | 0.0017 |  |  | sum | 432 | 0.94 | 0.62 |  |  |
| all male | AB/AB | 44 |  |  |  | all male | AB/AB | 31 |  |  |  |  |
|  | AB/LJS | 104 |  |  |  |  | AB/LJS | 82 |  |  |  |  |
|  | LJS/LJS | 33 |  |  |  |  | LJS/LJS | 18 |  |  |  |  |
|  | sum | 181 | 5.36 | 0.068 |  |  | sum | 131 | 10.89 | 0.0043 |  |  |
| 1st set nauplii | AB/AB | 22 |  |  |  | 1st set nauplii | AB/AB | 17 |  |  |  |  |
|  | AB/LJS | 44 |  |  |  |  | AB/LJS | 80 |  |  |  |  |
|  | LJS/LJS | 27 |  |  |  |  | LJS/LJS | 38 |  |  |  |  |
|  | sum | 93 | 0.81 | >0.9 |  |  | sum | 135 | 11.16 | 0.0038 |  |  |
| 2nd set nauplii | AB/AB | 14 |  |  |  | 2nd set nauplii | AB/AB | 23 |  |  |  |  |
|  | AB/LJS | 24 |  |  |  |  | AB/LJS | 64 |  |  |  |  |
|  | LJS/LJS | 17 |  |  |  |  | LJS/LJS | 32 |  |  |  |  |
|  | sum | 55 | 1.22 | 0.55 |  |  | sum | 119 | 2.04 | 0.36 |  |  |

**Table S2 (cont.)**

| Cross/  Progeny type | Gene/  Genotype | observed # | χ^2^ | P-value | M-F test^1^ | Cross/  Progeny type | Gene/  Genotype | observed # | χ^2^ | P-value | M-F test | Recip. Cross |
| --- | --- | --- | --- | --- | --- | --- | --- | --- | --- | --- | --- | --- |
| ABf x LJSm (cont.) | GOT2 (cont.) |  |  |  |  | ABmx LJSf (cont.) | GOT2 (cont.) |  |  |  |  |  |
| total nauplii | AB/AB | 36 |  |  |  | total nauplii | AB/AB | 40 |  |  |  |  |
|  | AB/LJS | 68 |  |  |  |  | AB/LJS | 144 |  |  |  |  |
|  | LJS/LJS | 44 |  |  |  |  | LJS/LJS | 70 |  |  |  |  |
|  | sum | 148 | 1.84 | 0.4 |  |  | sum | 254 | 11.64 | 0.003 |  |  |
| all adults | AB/AB | 96 |  |  |  | all adults | AB/AB | 133 |  |  |  |  |
|  | AB/LJS | 280 |  |  |  |  | AB/LJS | 308 |  |  |  |  |
|  | LJS/LJS | 99 |  |  |  |  | LJS/LJS | 122 |  |  |  | χ^2^ =2.27 |
|  | sum | 475 | 15.25 | 0.00048 |  |  | sum | 563 | 5.42 | 0.067 |  | p=0.32 |
|  |  |  |  |  |  |  |  |  |  |  |  |  |
| **SDf x LJSm** |  |  |  |  |  | **SDm x LJSf** |  |  |  |  |  |  |
|  | **ME2** |  |  |  |  |  | **ME2** |  |  |  |  | |
| all female | LJS/LJS | 83 |  |  |  | all female | LJS/LJS | 41 |  |  |  |  |
|  | LJS/SD | 163 |  |  |  |  | LJS/SD | 119 |  |  |  |  |
|  | SD/SD | 121 |  |  |  |  | SD/SD | 49 |  |  |  |  |
|  | sum | 367 | 12.45 | 0.00198 |  |  | sum | 209 | 4.64 | 0.098 |  |  |
| all male | LJS/LJS | 37 |  |  |  | all male | LJS/LJS | 10 |  |  |  |  |
|  | LJS/SD | 79 |  |  |  |  | LJS/SD | 48 |  |  |  |  |
|  | SD/SD | 59 |  |  |  |  | SD/SD | 15 |  |  |  |  |
|  | sum | 175 | 7.18 | 0.0276 |  |  | sum | 73 | 7.93 | 0.019 |  |  |
| nauplii | LJS/LJS | 29 |  |  |  | nauplii | LJS/LJS | 76 |  |  |  |  |
|  | LJS/SD | 99 |  |  |  |  | LJS/SD | 116 |  |  |  |  |
|  | SD/SD | 55 |  |  |  |  | SD/SD | 86 |  |  |  |  |
|  | sum | 183 | 8.62 | 0.013 |  |  | sum | 278 | 8.33 | 0.016 |  |  |
| all adults | LJS/LJS | 120 |  |  |  | all adults | LJS/LJS | 51 |  |  |  |  |
|  | LJS/SD | 242 |  |  |  |  | LJS/SD | 167 |  |  |  |  |
|  | SD/SD | 180 |  |  |  |  | SD/SD | 64 |  |  |  | χ^2^ =16.5 |
|  | sum | 542 | 19.49 | 0.00006 |  |  | sum | 282 | 10.79 | 0.0045 |  | p=0.0002 |

^1^M-F refers to tests for significant differences between males and females; these were presented in Table 2 for all crosses except the ABm x LJSf cross

^2^Recip. Cross is a comparison between the F_2_ adults for reciprocal crosses of the AB x LJS populations and SD x LJS populations.

^3^ F_2_ nauplii were only collected and genotyped for the 20˚C cross and not the 20-28˚C cross

^4^ ABm x LJSf cross is included here for comparison only. An examination of F_2_ nauplii results suggested that there could be some contamination

of this cross by pure LJS genotype individuals in F_1_ crosses; however, the concordance between results in adults (and little evidence for excess

LJS alleles) suggests that this contamination may not have extended to the F_2_ adults for this cross. Suspect nauplii data have been excluded

from the 1st set of nauplii, and no contamination is suspected for 2nd naupii set from this cross.

^5^ A second cross was done to bolster the numbers of nauplii genotyped for these crosses. These two sets are presented both separate and totaled

here and are shown totaled in paper.
