# Supplementary material for: Complex Deleterious Interactions Associated with Malic Enzyme May Contribute to Reproductive Isolation in the Copepod Tigriopus californicus
Source: PLoS One. 2011 Jun 22;6(6):e21177. doi: 10.1371/journal.pone.0021177 (PMC3120845; doi:10.1371/journal.pone.0021177)
Supplement: Table S3 — Test for two-locus deviations from independence in population crosses of T. californicus. (DOCX) [file pone.0021177.s004.docx]

**Table S3**

**Test for two-locus deviations from independence in population crosses of *T. californicus*.**

|  | *ME1/*  *ME2* | *ME1/*  *GOT2* | *ME1/*  *RISP* | *ME1/*  *CYC1* | *ME1/*  *CYC* | *ME2/*  *GOT2* | *ME2/*  *RISP* | *ME2/*  *CYC1* | *ME2/*  *CYC* | *GOT2/*  *RISP* | *GOT2/*  *CYC1* | *GOT2/*  *CYC* | *RISP/*  *CYC1* | *RISP/*  *CYC* | *CYC1/*  *CYC* |
| --- | --- | --- | --- | --- | --- | --- | --- | --- | --- | --- | --- | --- | --- | --- | --- |
| ABf x SDm 20˚ | | |  |  |  |  |  |  |  |  |  |  |  |  |  |
| Total adult χ^2^ | 6.63 | 4.74 | 12.2 | 2.87 | 6.11 | 75.8 | 13.7 | 4.70 | 3.65 | 122 | 9.98 | 4.43 | 0.85 | 15.4 | 9.10 |
| Total P-value | 0.15 | 0.32 | **0.016** | >0.5 | 0.19 | **<0.00001*** | **0.008** | 0.32 | 0.46 | **<0.00001*** | **0.041** | 0.35 | >0.5 | **0.004** | 0.06 |
| Nauplii χ^2^ | 1.52 | 4.44 | 1.54 | 1.94 | 2.85 | 1.85 | 2.06 | 6.62 | 4.33 | 44.3 | 3.96 | 3.72 | 2.81 | 6.12 | 1.50 |
| Nauplii P-value | >0.5 | 0.35 | >0.5 | >0.5 | >0.5 | >0.5 | >0.5 | 0.16 | 0.36 | **<0.00001*** | 0.41 | 0.46 | >0.5 | 0.19 | >0.5 |
| ABf x SDm 28˚ | | |  |  |  |  |  |  |  |  |  |  |  |  |  |
| Total adult χ^2^ | 6.66 | 9.70 | 4.15 | 5.16 | 4.39 | 19.2 | 5.6 | 0.26 | 10.4 | 114 | 2.82 | 3.37 | 4.88 | 2.94 | 3.71 |
| Total P-value | 0.15 | **0.046** | 0.39 | 0.27 | 0.36 | **0.0007*** | >0.5 | >0.5 | **0.035** | **<0.00001*** | >0.5 | 0.50 | 0.30 | >0.5 | >0.5 |
| ABf x LJSm |  |  |  |  |  |  |  |  |  |  |  |  |  |  |  |
| Total adult χ^2^ | 1.90 | 4.16 |  |  |  | 1.86 |  |  |  |  |  |  |  |  |  |
| Total P-value | >0.5 | 0.38 |  |  |  | >0.5 |  |  |  |  |  |  |  |  |  |
| Nauplii χ^2^ | 6.71 | 3.48 |  |  |  | 1.43 |  |  |  |  |  |  |  |  |  |
| Nauplii P-value | 0.15 | 0.48 |  |  |  | >0.5 |  |  |  |  |  |  |  |  |  |
| ABm x LJSf |  |  |  |  |  |  |  |  |  |  |  |  |  |  |  |
| Total adult χ^2^ | 2.52 | 1.26 |  |  |  | 1.80 |  |  |  |  |  |  |  |  |  |
| Total P-value | >0.5 | >0.5 |  |  |  | >0.5 |  |  |  |  |  |  |  |  |  |
| Nauplii χ^2^ | 7.07 | 5.39 |  |  |  | 7.07 |  |  |  |  |  |  |  |  |  |
| Nauplii P-value | 0.13 | 0.25 |  |  |  | 0.13 |  |  |  |  |  |  |  |  |  |

This table gives the χ^2^ deviations from expected two-locus numbers for pairs of loci (calculated by adjusting expected numbers by deviations seen for each single locus). For the ABf x SDm cross 20˚ is the 20˚ constant, while 28˚ is the 28˚-cycle. A P-value has been calculated using 4 d.f. (expected two-locus numbers can be calculated from knowing the frequencies of two genotypic classes for each of the two loci). P-values in bold are lower than 0.05 while those exceeding the sequential Bonferroni correction value of P=0.0011 are denoted with an *.
