# Supplementary material for: Complex Deleterious Interactions Associated with Malic Enzyme May Contribute to Reproductive Isolation in the Copepod Tigriopus californicus
Source: PLoS One. 2011 Jun 22;6(6):e21177. doi: 10.1371/journal.pone.0021177 (PMC3120845; doi:10.1371/journal.pone.0021177)
Supplement: File S1 — DNA sequences of GOT2 from T. californicus population samples. (DOC) [file pone.0021177.s005.doc]

**Supplemental sequence File S1**

**DNA sequences of GOT2 from *T. californicus* population samples**

#NEXUS

[NOTE1: Although two alleles are given for each individual, the phase of the multiple polymorphisms within a single individual has not been determined]

[NOTE2: Although the sequences are non-interleaved, the gaps in the sequences will give the correct alignment]

begin data;

dimensions ntax=61 nchar=1351;

format datatype=dna gap=- interleave=no;

matrix

GOT_2SDmRNA GGCCCCCCTGATCCTATTTTGGGCGTGAGTGAGGCCTTCAAAAAGGATACTAATCCCCTGAAAATGAATTTAGGGGTTGGTGCTTACCGAGATGATCAAGGCAAACCCTTTGTCTTGCCGTCTGTTCGTGAGGCCGAACGGATCATTGCTCAAAAAGGATTGAACAAAGAATACGCTCCAATTGGAGGTGAACCCGAGTTTGGCAGGCTTTCCGCTAACTTAGCATTTGGCCAAG---------------------------------------------------------------------------GAAATGAGATTGTTTCAAGTGGACGGAACGTGTCGGTTCAGACTATCTCGGGCACAGGTGCCTTGCGAGTAGGTGCCACTTATTTAGCCAAATGGTTCCCTGGGAACAAAACCGTGTATTTGCCCAGGCCTTCATGGGGCAATCATACCCCCATCTTTAAGCAAAGTGGGATGAATGTCGATGGTTATCGTTACTACGACCCTACAACTTGTGGATTCGATTTTAAAGGCGCCATGGAGGACATAANCCGGATCCCTGAAAAAAGCGTTATCATGCTTCACGCTTGCGCTCATAACCCAACCGGAGTCGATCCCAAGGATGAACAATGGAAAGAAATGTCACAATTGGTCAAGAAAAGGAATCTGTTTGCTTTCTTTGACATGGCTTATCAAGGCTTTGCTTCCGGTGACGTTGACAGAGATGCCTTTGCCGTCCGCCAATTTTTGGAAGATGGGCACAATATTTGTTTGTCCCAATCGTACGCCAAAAACATGGGTCTTTATGGCGAGCGAGTAGGAGCGTTTACTGTTGTTTGCAAA---------------------------------------------------------GATAAGGAAGAAGCAGCCCGCGTGAACTCTCAAATCAAGATCCTCATTAGACCCATGTATTCCAATCCTCCTGTTAATGGATCACGGATTGTGAGCGAGATTCTTACCAACACTGCTTTAAACAAGCAGTGGCTGGAAGATGTGAAGGGTATGGCTGACCGAATCATTACAATGAGACAGGAATTAAAGGATGGGTTGGCCAATGAAGGATCGAGCAAAAATTGGGAGCACATTGTGGATCAAATTGGAATGTTTTGTTTCACTGGAATGACCCCTGAACAG----------------------------------------------------------GTTGAAAAAATAACCACGGAATTTAGTGTTTACATGACCAAAGATGGCCGTATTTCAGTGGCGGGGATTTCCTCATCTAATGTTGGATATTTGGCTAAAGCCATG

SCN_5a_GOT2 NNNNNNNNNNNNNNNATTTTGGGCGTGAGCGAGGCCTTCAAAAAGGATACTAATCCCCTGAAAATGAATTTAGGGGTCGGTGCTTACCGAGATGATCAAGGTAAACCCTTTGTCTTGCCGTCTGTTCGTGAGGCCGAACGTATCATTGCTCAAAAAGGATTGAACAAAGAATACGCTCCAATTGGAGGTGAACCCGAGTTTGGCAGGCTATCCGCTAACTTAGCATTTGGCCAAGGTACGTAATCTCTGTCAAGGACGAATTGAGGAGCGTTCAGCTTAAATCAAAG-AAAAAAATGGTATCATCTTCAGGAAATGAGATTGTTTCAAGTGGACGGAACGTGTCGGTTCAGACTATCTCGGGCACAGGTGCCTTGCGAGTAGGTGCCACTTATTTAGCCAAATGGTTCCCTGGGAACAAAACCGTGTATTTGCCCAGGCCTTCATGGGGCAATCATACCCCCATCTTTAAGCAAAGTGGGATGAATGTCGATGGTTATCGTTACTACGACCCTACAACTTGTGGATTCGATTTTAATGGCGCCATGGAGGACATAAGCCGGATCCCTGAAAAAAGTGTTATCATGCTTCACGCTTGCGCTCACAACCCAACCGGAGTCGATCCCAAGGATGAACAATGGAAGGAAATGTCACAATTGGTCAAGAAAAGGAATTTGTTTGCTTTCTTTGACATGGCTTATCAAGGCTTTGCTTCTGGTGACGTTGACGGAGATGCCTTTGCCGTCCGCCAATTTTTGGAAGATGGGCACAATATTTGTTTGTCCCAATCGTACGCCAAAAACATGGGTCTTTATGGCGAGCGAGTAGGAGCGTTTACTGTTGTTTGCAAAGTAAGTATACGCGAAAAAAGGAAGAACCGAACTAGTAACATATTTGGTCAAATTTAGGACAAGGAAGAAGCAGCCCGCGTGAACTCTCAAATCAAGATCCTCATCAGACCCATGTATTCCAATCCTCCTGTTAATGGATCACGGATTGTGAGCGAGATTCTTACAAACACTACTTTAAACAAGCAGTGGCTGGAAGATGTGAAGGGTATGGCTGACCGAATCATTACAATGAGACAAGAATTAAAGGATGGATTGGCCAAGGAAGGATCGAGCAAAAATTGGGAGCACATTGTGGATCAAATTGGAATGTTTTGTTTCACAGGAATGACCCCTGAACAGG--TATTTGTTGTGATAACGGAAAAGGTCTCCATACTTAACTGTTTTTCTGTCTTCAGGTTGAAAAAATTACCAAGGACTTCAGTGTTTACATGACCAAAGATGGCCGTATTTCCGTGGCAGGGATTTCCTCTTCTAATGTTGGATATTTGGCTAAAGCCATG

SCN_5b_GOT2 NNNNNNNNNNNNNNNATTTTGGGCGTGAGCGAGGCCTTCAAAAAGGATACTAATCCCCTGAAAATGAATTTAGGGGTCGGTGCTTACCGAGATGATCAAGGTAAACCCTTTGTCTTGCCGTCTGTTCGTGAGGCCGAACGTATCATTGCTCAAAAAGGATTGAACAAAGAATACGCTCCAATTGGAGGTGAACCCGAGTTTGGCAGGCTATCCGCTAACTTAGCATTTGGCCAAGGTACGTAATCTCTGTCAAGGACGAATTGAGGAGCGTTCAGCTTAAATCAAAGAAAAAAAATGGTATCATCTTCAGGAAATGAGATTGTTTCAAGTGGACGGAACGTGTCGGTTCAGACTATCTCGGGCACAGGTGCCTTGCGAGTAGGTGCCACTTATTTAGCCAAATGGTTCCCTGGGAACAAAACCGTGTATTTGCCCAGGCCTTCATGGGGCAATCATACCCCCATCTTTAAGCAAAGTGGGATGAATGTCGATGGTTATCGTTACTACGACCCTACAACTTGTGGATTCGATTTTAATGGCGCCATGGAGGACATAAGCCGGATCCCTGAAAAAAGTGTTATCATGCTTCACGCTTGCGCTCACAACCCAACCGGAGTCGATCCCAAGGATGAACAATGGAAAGAAATGTCACAATTGGTCAAGAAAAGGAATTTGTTTGCTTTCTTTGACATGGCTTATCAAGGCTTTGCTTCTGGTGACGTTGACGGAGATGCCTTTGCCGTCCGCCAATTTTTGGAAGATGGGCACAATATTTGTTTGTCCCAATCGTACGCCAAAAACATGGGTCTTTATGGCGAGCGAGTAGGAGCGTTTACTGTGGTTTGCAAAGTAAGTGTACGCGAAAAAAGGAAGAACCGAACTAGTAACATATTTGGTTAAATTTAGGACAAGGAAGAAGCAGCCCGCGTGAACTCTCAAATCAAGATCCTCATCAGACCCATGTATTCCAATCCTCCTGTTAATGGATCACGGATTGTGAGCGAGATTCTTACAAACACTACTTTAAACAAGCAGTGGCTGGAAGATGTGAAGGGTATGGCTGACCGAATCATTACAATGAGACAAGAATTAAAGGATGGATTGGCCAAGGAAGGATCGAGCAAAAATTGGGAGCACATTGTGGATCAAATTGGAATGTTTTGTTTCACAGGAATGACCCCTGAACAGG--TATTTATTGTGATAACAGAAAAGGTCTCGATACTTAAATGTTTTTCTGTCTTCAGGTTGAAAAAATTACCAAGGACTTCAGTGTTTACATGACCAAAGATGGCCGTATTTCCGTGGCAGGGATTTCCTCTTCTAATGTTGGATATTTGGCTAAAGCCATG

SCN_m5_GOT2a GGCCCCCCTGATCCTATTTTGGGCGTGAGCGAGGCCTTCAAAAAGGATACTAATCCCCTGAAAATGAATTTAGGGGTCGGTGCTTACCGAGATGATCAAGGTAAACCCTTTGTCTTGCCGTCTGTTCGTGAGGCCGAACGTATCATTGCTCAAAAAGGATTGAACAAAGAATACGCTCCAATTGGAGGTGAACCCGAGTTTGGCAGGCTATCCGCTAACTTAGCATTTGGCCAAGGTACGTAATCTCTGTCAAGGACGAATTGAGGAGCGTTCAGCTTAAATCAAAG-AAAAAAATGGTATCATCTTCAGGAAATGAGATTGTTTCAAGTGGACGGAACGTGTCGGTTCAGACTATCTCGGGCACAGGTGCCTTGCGAGTAGGTGCCACTTATTTAGCCAAATGGTTCCCTGGGAACAAAACCGTGTATTTGCCCAGGCCTTCATGGGGCAATCATACCCCCATCTTTAAGCAAAGTGGGATGAATGTCGATGGTTATCGTTACTACGACCCTACAACTTGTGGATTCGATTTTAATGGCGCCATGGAGGACATAAGCCGGATCCCTGAAAAAAGTGTTATCATGCTTCACGCTTGCGCTCACAACCCAACCGGAGTCGATCCCAAGGATGAACAATGGAAAGAAATGTCACAATTGGTCAAGAAAAGGAATTTGTTTGCTTTCTTTGACATGGCTTATCAAGGCTTTGCTTCTGGTGACGTTGACGGAGATGCCTTTGCCGTCCGCCAATTTTTGGAAGATGGGCACAATATTTGTTTGTCCCAATCGTACGCCAAAAACATGGGTCTTTATGGCGAGCGAGTAGGAGCGTTTACTGTGGTTTGCAAAGTAAGTGTACGCGAAAAAAGGAAGAACCGAACTAGTAACATATTTGGTTAAATTTAGGACAAGGAAGAAGCAGCCCGCGTGAACTCTCAAATCAAGATCCTCATCAGACCCATGTATTCCAATCCTCCTGTTAATGGATCACGGATTGTGAGCGAGATTCTTACAAACACTACTTTAAACAAGCAGTGGCTGGAAGATGTGAAGGGTATGGCTGACCGAATCATTACAATGAGACAAGAATTAAAGGATGGATTGGCCAAGGAAGGATCGAGCAAAAATTGGGAGCACATTGTGGATCAAATTGGAATGTTTTGTTTCACAGGAATGACCCCTGAACAGG--TATTTATTGTGATAACAGAAAAGGTCTCGATACTTAAATGTTTTTCTGTCTTCAGGTTGAAAAAATTACCAAGGACTTCAGTGTTTACATGACCAAAGATGGCCGTATTTCCGTGGCAGGGATTTCCTCTTCTAATGTTGGATATTTGGCTAAAGCCATG

SCN_m5_GOT2b GGCCCCCCTGATCCTATTTTGGGCGTGAGCGAGGCCTTCAAAAAGGATACTAATCCCCTGAAAATGAATTTAGGGGTCGGTGCTTACCGAGATGATCAAGGTAAACCCTTTGTCTTGCCGTCTGTTCGTGAGGCCGAACGTATCATTGCTCAAAAAGGATTGAACAAAGAATACGCTCCAATTGGAGGTGAACCCGAGTTTGGCAGGCTATCCGCTAACTTAGCATTTGGCCAAGGTACGTAATCTCTGTCAAGGACGAATTGAGGAGCGTTCAGCTTAAATCAAAG-AAAAAAATGGTATCATCTTCAGGAAATGAGATTGTTTCAAGTGGACGGAACGTGTCGGTTCAGACTATCTCGGGCACAGGTGCCTTGCGAGTAGGTGCCACTTATTTAGCCAAATGGTTCCCTGGGAACAAAACCGTGTATTTGCCCAGGCCTTCATGGGGCAATCATACCCCCATCTTTAAGCAAAGTGGGATGAATGTCGATGGTTATCGTTACTACGACCCTACAACTTGTGGATTCGATTTTAATGGCGCCATGGAGGACATAAGCCGGATCCCTGAAAAAAGTGTTATCATGCTTCACGCTTGCGCTCACAACCCAACCGGAGTCGATCCCAAGGATGAACAATGGAAAGAAATGTCACAATTGGTCAAGAAAAGGAATTTGTTTGCTTTCTTTGACATGGCTTATCAAGGCTTTGCTTCTGGTGACGTTGACGGAGATGCCTTTGCCGTCCGCCAATTTTTGGAAGATGGGCACAATATTTGTTTGTCCCAATCGTACGCCAAAAACATGGGTCTTTATGGCGAGCGAGTAGGAGCGTTTACTGTGGTTTGCAAAGTAAGTGTACGCGAAAAAAGGAAGAACCGAACTAGTAACATATTTGGTTAAATTTAGGACAAGGAAGAAGCAGCCCGCGTGAACTCTCAAATCAAGATCCTCATCAGACCCATGTATTCCAATCCTCCTGTTAATGGATCACGGATTGTGAGCGAGATTCTTACAAACACTACTTTAAACAAGCAGTGGCTGGAAGATGTGAAGGGTATGGCTGACCGAATCATTACAATGAGACAAGAATTAAAGGATGGATTGGCCAAGGAAGGATCGAGCAAAAATTGGGAGCACATTGTGGATCAAATTGGAATGTTTTGTTTCACAGGAATGACCCCTGAACAGG--TATTTATTGTGATAACAGAAAAGGTCTCGATACTTAAATGTTTTTCTGTCTTCAGGTTGAAAAAATTACCAAGGACTTCAGTGTTTACATGACCAAAGATGGCCGTATTTCCGTGGCAGGGATTTCCTCTTCTAATGTTGGATATTTGGCTAAAGCCATG

SCN_m6_GOT2a GGCCCCCCTGATCCTATTTTGGGCGTGAGTGAGGCCTTCAAAAAGGATACTAATCCCCTGAAAATGAATTTAGGGGTCGGTGCTTACCGAGATGATCAAGGTAAACCCTTTGTCTTGCCGTCTGTTCGTGAGGCCGAACGTATCATTGCTCAAAAAGGATTGAACAAAGAATACGCTCCAATTGGAGGTGAACCCGAGTTTGGCAGGCTATCCGCTAACTTAGCATTTGGCCAAGGTACGTAATCTCTGTCAAGGACGAATTGAGGAGCGTTCAGCTTAAATCAAAG-AAAAAAATGGTATCATCTTCAGGAAATGAGATTGTTTCAAGTGGACGGAACGTGTCGGTTCAGACTATCTCGGGCACAGGTGCCTTGCGAGTAGGTGCCACTTATTTAGCCAAATGGTTCCCTGGGAACAAAACCGTGTATTTGCCCAGGCCTTCATGGGGCAATCATACCCCCATCTTTAAGCAAAGTGGGATGAATGTCGATGGTTATCGTTACTACGACCCTACAACTTGTGGATTCGATTTTAATGGCGCCATGGAGGACATAAGCCGGATCCCTGAAAAAAGTGTTATCATGCTTCACGCTTGCGCTCACAACCCAACCGGAGTCGATCCCAAGGATGAACAATGGAAGGAAATGTCACAATTGGTCAAGAAAAGGAATTTGTTTGCTTTCTTTGACATGGCTTATCAAGGCTTTGCTTCTGGTGACGTTGACGGAGATGCCTTTGCCGTCCGCCAATTTTTGGAAGATGGGCACAATATTTGTTTGTCCCAATCGTACGCTAAAAACATGGGTCTTTATGGCGAGCGAGTAGGAGCGTTTACTGTTGTTTGCAAAGTAAGTATACGCGAAAAAAGGAAGAACCGAACTAGTAACATATTTGGTCAAATTTAGGACAAGGAAGAAGCAGCCCGCGTGAACTCTCAAATCAAGATCCTCATCAGACCCATGTATTCCAATCCTCCTGTTAATGGATCACGGATTGTGAGCGAGATTCTTACAAACACTACTTTAAACAAGCAGTGGCTGGAAGATGTGAAGGGTATGGCTGACCGAATCATTACAATGAGACAAGAATTAAAGGATGGATTGGCCAAGGAAGGATCGAGCAAAAATTGGGAGCACATTGTGGATCAAATTGGAATGTTTTGTTTCACAGGAATGACCCCTGAACAGG--TATTTGTTGTGATAACGGAAAAGGTCTCCATACTTAACTGTTTTTCTGTCTTCAGGTTGAAAAAATTACCAAGGACTTCAGTGTTTACATGACCAAAGATGGCCGTATTTCCGTGGCAGGGATTTCCTCTTCTAATGTTGGATATTTGGCTAAAGCCATG

SCN_m6_GOT2b GGCCCCCCTGATCCTATTTTGGGCGTGAGTGAGGCCTTCAAAAAGGATACTAATCCCCTGAAAATGAATTTAGGGGTCGGTGCTTACCGAGATGATCAAGGTAAACCCTTTGTCTTGCCGTCTGTTCGTGAGGCCGAACGTATCATTGCTCAAAAAGGATTGAACAAAGAATACGCTCCAATTGGAGGTGAACCCGAGTTTGGCAGGCTATCCGCTAACTTAGCATTTGGCCAAGGTACGTAATCTCTGTCAAGGACGAATTGAGGAGCGTTCAGCTTAAATCAAAG-AAAAAAATGGTATCATCTTCAGGAAATGAGATTGTTTCAAGTGGACGGAACGTGTCGGTTCAGACTATCTCGGGCACAGGTGCCTTGCGAGTAGGTGCCACTTATTTAGCCAAATGGTTCCCTGGGAACAAAACCGTGTATTTGCCCAGGCCTTCATGGGGCAATCATACCCCCATCTTTAAGCAAAGTGGGATGAATGTCGATGGTTATCGTTACTACGACCCTACAACTTGTGGATTCGATTTTAATGGCGCCATGGAGGACATAAGCCGGATCCCTGAAAAAAGTGTTATCATGCTTCACGCTTGCGCTCACAACCCAACCGGAGTCGATCCCAAGGATGAACAATGGAAGGAAATGTCACAATTGGTCAAGAAAAGGAATTTGTTTGCTTTCTTTGACATGGCTTATCAAGGCTTTGCTTCTGGTGACGTTGACGGAGATGCCTTTGCCGTCCGCCAATTTTTGGAAGATGGGCACAATATTTGTTTGTCCCAATCGTACGCTAAAAACATGGGTCTTTATGGCGAGCGAGTAGGAGCGTTTACTGTTGTTTGCAAAGTAAGTATACGCGAAAAAAGGAAGAACCGAACTAGTAACATATTTGGTCAAATTTAGGACAAGGAAGAAGCAGCCCGCGTGAACTCTCAAATCAAGATCCTCATCAGACCCATGTATTCCAATCCTCCTGTTAATGGATCACGGATTGTGAGCGAGATTCTTACAAACACTACTTTAAACAAGCAGTGGCTGGAAGATGTGAAGGGTATGGCTGACCGAATCATTACAATGAGACAAGAATTAAAGGATGGATTGGCCAAGGAAGGATCGAGCAAAAATTGGGAGCACATTGTGGATCAAATTGGAATGTTTTGTTTCACAGGAATGACCCCTGAACAGG--TATTTGTTGTGATAACGGAAAAGGTCTCCATACTTAACTGTTTTTCTGTCTTCAGGTTGAAAAAATTACCAAGGACTTCAGTGTTTACATGACCAAAGATGGCCGTATTTCCGTGGCAGGGATTTCCTCTTCTAATGTTGGATATTTGGCTAAAGCCATG

SCN_21_a_GOT2 GGCCCCCCTGATCCTATTTTGGGCGTGAGTGAGGCCTTCAAAAAGGATACTAATCCCCTGAAAATGAATTTAGGGGTCGGTGCTTACCGAGATGATCAAGGTAAACCCTTTGTCTTGCCGTCTGTTCGTGAGGCCGAACGTATCATTGCTCAAAAAGGATTGAACAAAGAATACGCTCCAATTGGAGGTGAACCCGAGTTTGGCAGGCTATCCGCTAACTTAGCATTTGGCCAAGGTACGTAATCTCTGTCAAGGACGAATTGAGGAGCGTTCAGCTTAAATCAAAG-AAAAAAATGGTATCATCTTCAGGAAATGAGATTGTTTCAAGTGGACGGAACGTGTCGGTTCAGACTATCTCGGGCACAGGTGCCTTGCGAGTAGGTGCCACTTATTTAGCCAAATGGTTCCCTGGGAACAAAACCGTGTATTTGCCCAGGCCTTCATGGGGCAATCATACCCCCATCTTTAAGCAAAGTGGGATGAATGTCGATGGTTATCGTTACTACGACCCTACAACTTGTGGATTCGATTTTAATGGCGCCATGGAGGACATAAGCCGGATCCCTGAAAAAAGTGTTATCATGCTTCACGCTTGCGCTCACAACCCAACCGGAGTCGATCCCAAGGATGAACAATGGAAGGAAATGTCACAATTGGTCAAGAAAAGGAATTTGTTTGCTTTCTTTGACATGGCTTATCAAGGCTTTGCTTCTGGTGACGTTGACGGAGATGCCTTTGCCGTCCGCCAATTTTTGGAAGATGGGCACAATATTTGTTTGTCCCAATCGTACGCCAAAAACATGGGTCTTTATGGCGAGCGAGTAGGAGCGTTTACTGTTGTTTGCAAAGTAAGTATACGCGAAAAAAGGAAGAACCGAACTAGTAACATATTTGGTCAAATTTAGGACAAGGAAGAAGCAGCCCGCGTGAACTCTCAAATCAAGATCCTCATCAGACCCATGTATTCCAATCCTCCTGTTAATGGATCACGGATTGTGAGCGAGATTCTTACAAACACTACTTTAAACAAGCAGTGGCTGGAAGATGTGAAGGGTATGGCTGACCGAATCATTACAATGAGACAAGAATTAAAGGATGGATTGGCCAAGGAAGGATCGAGCAAAAATTGGGAGCACATTGTGGATCAAATTGGAATGTTTTGTTTCACAGGAATGACCCCTGAACAGG--TATTTGTTGTGATAACGGAAAAGGTCTCCATACTTAACTGTTTTTCTGTCTTCAGGTTGAAAAAATTACCAAGGACTTCAGTGTTTACATGACCAAAGATGGCCGTATTTCCGTGGCAGGGATTTCCTCTTCTAATGTTGGATATTTGGCTAAAGCCATG

SCN_21_b_GOT2a GGCCCCCCTGATCCTATTTTGGGCGTGAGTGAGGCCTTCAAAAAGGATACTAATCCCCTGAAAATGAATTTAGGGGTCGGTGCTTACCGAGATGATCAAGGTAAACCCTTTGTCTTGCCGTCTGTTCGTGAGGCCGAACGTATCATTGCTCAAAAAGGATTGAACAAAGAATACGCTCCAATTGGAGGTGAACCCGAGTTTGGCAGGCTATCCGCTAACTTAGCATTTGGCCAAGGTACGTAATCTCTGTCAAGGACGAATTGAGGAGCGTTCAGCTTAAATCAAAG-AAAAAAATGGTATCATCTTCAGGAAATGAGATTGTTTCAAGTGGACGGAACGTGTCGGTTCAGACTATCTCGGGCACAGGTGCCTTGCGAGTAGGTGCCACTTATTTAGCCAAATGGTTCCCTGGGAACAAAACCGTGTATTTGCCCAGGCCTTCATGGGGCAATCATACCCCCATCTTTAAGCAAAGTGGGATGAATGTCGATGGTTATCGTTACTACGACCCTACAACTTGTGGATTCGATTTTAATGGCGCCATGGAGGACATAAGCCGGATCCCTGAAAAAAGTGTTATCATGCTTCACGCTTGCGCTCACAACCCAACCGGAGTCGATCCCAAGGATGAACAATGGAAGGAAATGTCACAATTGGTCAAGAAAAGGAATTTGTTTGCTTTCTTTGACATGGCTTATCAAGGCTTTGCTTCTGGTGACGTTGACGGAGATGCCTTTGCCGTCCGCCAATTTTTGGAAGATGGGCACAATATTTGTTTGTCCCAATCGTACGCCAAAAACATGGGTCTTTATGGCGAGCGAGTAGGAGCGTTTACTGTTGTTTGCAAAGTAAGTATACGCGAAAAAAGGAAGAACCGAACTAGTAACATATTTGGTCAAATTTAGGACAAGGAAGAAGCAGCCCGCGTGAACTCTCAAATCAAGATCCTCATCAGACCCATGTATTCCAATCCTCCTGTTAATGGATCACGGATTGTGAGCGAGATTCTTACAAACACTACTTTAAACAAGCAGTGGCTGGAAGATGTGAAGGGTATGGCTGACCGAATCATTACAATGAGACAAGAATTAAAGGATGGATTGGCCAAGGAAGGATCGAGCAAAAATTGGGAGCACATTGTGGATCAAATTGGAATGTTTTGTTTCACAGGAATGACCCCTGAACAGG--TATTTGTTGTGATAACGGAAAAGGTCTCCATACTTAACTGTTTTTCTGTCTTCAGGTTGAAAAAATTACCAAGGACTTCAGTGTTTACATGACCAAAGATGGCCGTATTTCCGTGGCAGGGATTTCCTCTTCTAATGTTGGATATTTGGCTAAAGCCATG

GOT2_SS2_A GGCCCCCCTGATCNTATTTTGGGCGTGAGCGAGGCCTTCAAAAAGGATACTAATCCCCTGAAAATGAATTTAGGGGTTGGTGCTTACCGAGATGATCAAGGCAAACCCTTTGTCTTGCCGTCTGTTCGTGAGGCCGAACGTATCATTGCTCAAAAGGGATTGAACAAAGAATACGCTCCAATTGGAGGTGAACCCGAGTTTGGCAGGCTATCCGCTAACTTAGCATTTGGCCAAGGTACGTAATCTCTGTCAAGGATGAATGGAAGAGCGTTCAGCTTAAATCAATG-AAAAAAATTGTATCATCTTTAGGAAATGAGATTGTTTCAAGTGGACGGAACGTGTCGGTTCAAACTATCTCTGGCACAGGTGCCTTGCGAGTAGGTGCCACTTATTTAGCCAAATGGTTCCCTGGGAACAAAACCGTGTATTTGCCCAAGCCTTCATGGGGCAATCATACCCCCATCTTTAAGCAAAGTGGGATGAATGTCGATGGTTATCGTTACTACGACCCTACAACATGTGGATTCGATTTTAATGGCGCCATGGAGGACATAAGCCGGATCCCTGAAAAAAGTGTTATTATGCTTCACGCTTGCGCTCATAACCCAACCGGAGTCGATCCCAAGGATGAGCAATGGAAAGAAATGTCACAATTGGTCAAGAAAAGGAATTTGTTTGCTTTCTTTGACATGGCTTATCAAGGCTTTGCTTCCGGCGANNNNNNNNNNNNNNNNNNNNNNNNNNNNCAATTTTTGGAAGATGGGCACAATATTTGTTTGTCCCAATCGTACGCCAAAAACATGGGTCTTTATGGCGAGCGAGTAGGAGCGTTTACTGTTGTTTGCAAAGTAAGTAGACGCGAAAAAAGGAAGAACCGAACTAGTAATAAATTTGGTCAAATTTAGGACAAGGAAGAAGCAGCCCGCGTGAACTCTCAAATCAAGATCCTCATCAGACCCATGTATTCCAATCCTCCTGTTAATGGATCACGGATTGTGAGCGAGATTCTTACCAACACTACTTTAAACAAGCAGTGGCTGGAAGATGTGAAGGGTATGGCTGACCGAATCATTACAATGAGACAAGAATTAAAGGATGGATTGGCCAAGGAAGGATCGAGCAAAAATTGGGAGCACATTGTGGATCAAATTGGAATGTTTTGTTTCACAGGAATGACCCCTGAGCAGGTATATTTGTTGTGATAAGGGAAAAGGTCTCCATACTTAACTGTTTTTCTGTCTTCAGGTTGAAAAAATTACCAAGGACTTTAGTGTTTACATGACCAAAGATGGCCGTNTTTCCGTGGCAGGGATTTCCTCTTCTAATGTTGGATATTTGGCTAAAGCCATG

GOT2_SS2_B GGCCCCCCTGATCNTATTTTGGGCGTGAGCGAGGCCTTCAAAAAGGATACTAATCCCCTGAAAATGAATTTAGGGGTTGGTGCTTACCGAGATGATCAAGGCAAACCCTTTGTCTTGCCGTCTGTTCGTGAGGCCGAACGTATCATTGCTCAAAAGGGATTGAACAAAGAATACGCTCCAATTGGAGGTGAACCCGAGTTTGGCAGGCTATCCGCTAACTTAGCATTTGGCCAAGGTACGTAATCTCTGTCAAGGAAGAATGGAAGAGCGTTCAGCTTAAATCAATG-AAAAAAATTGTATCATCTTTAGGAAATGAGATTGTTTCAAGTGGACGGAACGTGTCGGTTCAAACTATCTCTGGCACAGGTGCCTTGCGAGTAGGTGCCACTTATTTAGCCAAATGGTTCCCTGGGAACAAAACCGTGTATTTGCCCAAGCCTTCATGGGGCAATCATACCCCCATCTTTAAGCAAAGTGGGATGAATGTCGATGGTTATCGTTACTACGACCCTACAACATGTGGATTCGATTTTAATGGCGCCATGGAGGACATAAGCCGGATCCCTGAAAAAAGTGTTATTATGCTTCACGCTTGCGCTCATAACCCAACCGGAGTCGATCCCAAGGATGAGCAATGGAAAGAAATGTCACAATTGGTCAAGAAAAGGAATTTGTTTGCTTTCTTTGACATGGCTTATCAAGGCTTTGCTTCCGGCGANNNNNNNNNNNNNNNNNNNNNNNNNNNNCAATTTTTGGAAGATGGGCACAATATTTGTTTGTCCCAATCGTACGCCAAAAACATGGGTCTTTATGGCGAGCGAGTAGGAGCGTTTACTGTTGTTTGCAAAGTAAGTAGACGCGAAAAAAGGAAGAACCGAACTAGTAATAAATTTGGTCAAATTTAGGACAAGGAAGAAGCAGCCCGCGTGAACTCTCAAATCAAGATCCTCATCAGACCCATGTATTCCAATCCTCCTGTTAATGGATCACGGATTGTGAGCGAGATTCTTACCAACACTACTTTAAACAAGCAGTGGCTGGAAGATGTGAAGGGTATGGCTGACCGAATCATTACAATGAGACAAGAATTAAAGGATGGATTGGCCAAGGAAGGATCGAGCAAAAATTGGGAGCACATTGTGGATCAAATTGGAATGTTTTGTTTCACAGGAATGACCCCTGAGCAGGTATATTTGTTGTGATAAGGGAAAAGGTCTCCATACTTAACTGTTTTTCTGTCTTCAGGTTGAAAAAATTACCAAGGACTTTAGTGTTTACATGACCAAAGATGGCCGTNTTTCCGTGGCAGGGATTTCCTCTTCTAATGTTGGATATTTGGCTAAAGCCATG

GOT2_SS5_A GGCCCCCCTGATCCTATTTTGGGCGTGAGCGAGGCCTTCAAAAAGGATACTAATCCCCTGAAAATGAATTTAGGGGTTGGTGCTTACCGAGATGATCAAGGCAAACCCTTTGTCTTGCCGTCTGTTCGTGAGGCCGAACGTATCATTGCTCAAAAGGGATTGAACAAAGAATACGCTCCAATTGGAGGTGAACCCGAGTTTGGCAGGCTATCCGCTAACTTAGCATTTGGCCAAGGTACGTAATCTCTGTCAAGGATGAATGGAAGAGCGTTCAGCTTAAATCAATG-AAAAAAATTGTATCATCTTTAGGAAATGAGATTGTTTCAAGTGGACGGAACGTGTCGGTTCAAACTATCTCTGGCACAGGTGCCTTGCGAGTAGGTGCCACTTATTTAGCCAAATGGTTCCCTGGGAACAAAACCGTGTATTTGCCCAAGCCTTCATGGGGCAATCATACCCCCATCTTTAAGCAAAGTGGGATGAATGTCGATGGTTATCGTTACTACGACCCTACAACATGTGGATTCGATTTTAATGGCGCCATGGAGGACATAAGCCGGATCCCTGAAAAAAGTGTTATTATGCTTCACGCTTGCGCTCATAACCCAACCGGAGTCGATCCCAAGGATGAGCAATGGAAAGAAATGTCACAATTGGTCAAGAAAAGGAATTTGTTTGCTTTCTTTGACATGGCTTATCAAGGCTTTGCTTCCGGCGACGTTGACGGAGATGCCTTTGCCGTCCGCCAATTTTTGGAAGATGGGCACAATATTTGTTTGTCCCAATCGTACGCCAAAAACATGGGTCTTTATGGCGAGCGAGTAGGAGCGTTTACTGTTGTTTGCAAAGTAAGTAGACGCGAAAAAAGGAAGAACCGAACTAGTAATAAATTTGGTCAAATTTAGGACAAGGAAGAAGCAGCCCGCGTGAACTCTCAAATCAAGATCCTCATCAGACCCATGTATTCCAATCCTCCTGTTAATGGATCACGGATTGTGAGCGAGATTCTTACCAACACTACTTTAAACAAGCAGTGGCTGGAAGATGTGAAGGGTATGGCTGACCGAATCATTACAATGAGACAAGAATTAAAGGATGGATTGGCCAAGGAAGGATCGAGCAAAAATTGGGAGCACATTGTGGATCAAATTGGAATGTTTTGTTTCACAGGAATGACCCCTGAGCAGGTATATTTGTTGTGATAAGGGAAAAGGTCTCCATACTTAACTGTTTTTCTGTCTTCAGGTTGAAAAAATTACCAAGGACTTTAGTGTTTACATGACCAAAGATGGCCGTATTTCCGTGGCAGGGATTTCCTCTTCTAATGTTGGATATTTGGCTAAAGCCATG

GOT2_SS5_B GGCCCCCCTGATCCTATTTTGGGCGTGAGCGAGGCCTTCAAAAAGGATACTAATCCCCTGAAAATGAATTTAGGGGTTGGTGCTTACCGAGATGATCAAGGCAAACCCTTTGTCTTGCCGTCTGTTCGTGAGGCCGAACGTATCATTGCTCAAAAGGGATTGAACAAAGAATACGCTCCAATTGGAGGTGAACCCGAGTTTGGCAGGCTATCCGCTAACTTAGCATTTGGCCAAGGTACGTAATCTCTGTCAAGGAAGAATGGAAGAGCGTTCAGCTTAAATCAATG-AAAAAAATTGTATCATCTTTAGGAAATGAGATTGTTTCAAGTGGACGGAACGTGTCGGTTCAAACTATCTCTGGCACAGGTGCCTTGCGAGTAGGTGCCACTTATTTAGCCAAATGGTTCCCTGGGAACAAAACCGTGTATTTGCCCAAGCCTTCATGGGGCAATCATACCCCCATCTTTAAGCAAAGTGGGATGAATGTCGATGGTTATCGTTACTACGACCCTACAACATGTGGATTCGATTTTAATGGCGCCATGGAGGACATAAGCCGGATCCCTGAAAAAAGTGTTATTATGCTTCACGCTTGCGCTCATAACCCAACCGGAGTCGATCCCAAGGATGAGCAATGGAAAGAAATGTCACAATTGGTCAAGAAAAGGAATTTGTTTGCTTTCTTTGACATGGCTTATCAAGGCTTTGCTTCCGGCGACGTTGACGGAGATGCCTTTGCCGTCCGCCAATTTTTGGAAGATGGGCACAATATTTGTTTGTCCCAATCGTACGCCAAAAACATGGGTCTTTATGGCGAGCGAGTAGGAGCGTTTACTGTTGTTTGCAAAGTAAGTAGACGCGAAAAAAGGAAGAACCGAACTAGTAATAAATTTGGTCAAATTTAGGACAAGGAAGAAGCAGCCCGCGTGAACTCTCAAATCAAGATCCTCATCAGACCCATGTATTCCAATCCTCCTGTTAATGGATCACGGATTGTGAGCGAGATTCTTACCAACACTACTTTAAACAAGCAGTGGCTGGAAGATGTGAAGGGTATGGCTGACCGAATCATTACAATGAGACAAGAATTAAAGGATGGATTGGCCAAGGAAGGATCGAGCAAAAATTGGGAGCACATTGTGGATCAAATTGGAATGTTTTGTTTCACAGGAATGACCCCTGAGCAGGTATATTTGTTGTGATAAGGGAAAAGGTCTCCATACTTAACTGTTTTTCTGTCTTCAGGTTGAAAAAATTACCAAGGACTTTAGTGTTTACATGACCAAAGATGGCCGTATTTCCGTGGCAGGGATTTCCTCTTCTAATGTTGGATATTTGGCTAAAGCCATG

GOT2_SS8_A GGCCCCCCTGATCCTATTTTGGGCGTGAGCGAGGCCTTCAAAAAGGATACTAATCCCCTGAAAATGAATTTAGGGGTTGGTGCTTACCGAGATGATCAAGGCAAACCCTTTGTCTTGCCGTCTGTTCGTGAGGCCGAACGTATCATTGCTCAAAAGGGATTGAACAAAGAATACGCTCCAATTGGAGGTGAACCCGAGTTTGGCAGGCTATCCGCTAACTTAGCATTTGGCCAAGGTACGTAATCTCTGTCAAGGATGAATGGAAGAGCGTTCAGCTTAAATCAATG-AAAAAAATTGTATCATCTTTAGGAAATGAGATTGTTTCAAGTGGACGGAACGTGTCGGTTCAAACTATCTCTGGCACAGGTGCCTTGCGAGTAGGTGCCACTTATTTAGCCAAATGGTTCCCTGGGAACAAAACCGTGTATTTGCCCAAGCCTTCATGGGGCAATCATACCCCCATCTTTAAGCAAAGTGGGATGAATGTCGATGGTTATCGTTACTACGACCCTACAACATGTGGATTCGATTTTAATGGCGCCATGGAGGACATAAGCCGGATCCCTGAAAAAAGTGTTATTATGCTTCACGCTTGCGCTCATAACCCAACCGGAGTCGATCCCAAGGATGAGCAATGGAAAGAAATGTCACAATTGGTCAAGAAAAGGAATTTGTTTGCTTTCTTTGACATGGCTTATCAAGGCTTTGCTTCCGGCGACGTTGACGGAGATGCCTTTGCCGTCCGCCAATTTTTGGAAGATGGGCACAATATTTGTTTGTCCCAATCGTACGCCAAAAACATGGGTCTTTATGGCGAGCGAGTAGGAGCGTTTACTGTTGTTTGCAAAGTAAGTAGACGCGAAAAAAGGAAGAACCGAACTAGTAATAAATTTGGTCAAATTTAGGACAAGGAAGAAGCAGCCCGCGTGAACTCTCAAATCAAGATCCTCATCAGACCCATGTATTCCAATCCTCCTGTTAATGGATCACGGATTGTGAGCGAGATTCTTACCAACACTACTTTAAACAAGCAGTGGCTGGAAGATGTGAAGGGTATGGCTGACCGAATCATTACAATGAGACAAGAATTAAAGGATGGATTGGCCAAGGAAGGATCGAGCAAAAATTGGGAGCACATTGTGGATCAAATTGGAATGTTTTGTTTCACAGGAATGACCCCTGAGCAGGTATATTTGTTGTGATAAGGGAAAAGGTCTCCATACTTAACTGTTTTTCTGTCTTCAGGTTGAAAAAATTACCAAGGACTTTAGTGTTTACATGACCAAAGATGGCCGTATTTCCGTGGCAGGGATTTCCTCTTCTAATGTTGGATATTTGGCTAAAGCCATG

GOT2_SS8_B GGCCCCCCTGATCCTATTTTGGGCGTGAGCGAGGCCTTCAAAAAGGATACTAATCCCCTGAAAATGAATTTAGGGGTTGGTGCTTACCGAGATGATCAAGGCAAACCCTTTGTCTTGCCGTCTGTTCGTGAGGCCGAACGTATCATTGCTCAAAAGGGATTGAACAAAGAATACGCTCCAATTGGAGGTGAACCCGAGTTTGGCAGGCTATCCGCTAACTTAGCATTTGGCCAAGGTACGTAATCTCTGTCAAGGAAGAATGGAAGAGCGTTCAGCTTAAATCAATG-AAAAAAATTGTATCATCTTTAGGAAATGAGATTGTTTCAAGTGGACGGAACGTGTCGGTTCAAACTATCTCTGGCACAGGTGCCTTGCGAGTAGGTGCCACTTATTTAGCCAAATGGTTCCCTGGGAACAAAACCGTGTATTTGCCCAAGCCTTCATGGGGCAATCATACCCCCATCTTTAAGCAAAGTGGGATGAATGTCGATGGTTATCGTTACTACGACCCTACAACATGTGGATTCGATTTTAATGGCGCCATGGAGGACATAAGCCGGATCCCTGAAAAAAGTGTTATTATGCTTCACGCTTGCGCTCATAACCCAACCGGAGTCGATCCCAAGGATGAGCAATGGAAAGAAATGTCACAATTGGTCAAGAAAAGGAATTTGTTTGCTTTCTTTGACATGGCTTATCAAGGCTTTGCTTCCGGCGACGTTGACGGAGATGCCTTTGCCGTCCGCCAATTTTTGGAAGATGGGCACAATATTTGTTTGTCCCAATCGTACGCCAAAAACATGGGTCTTTATGGCGAGCGAGTAGGAGCGTTTACTGTTGTTTGCAAAGTAAGTAGACGCGAAAAAAGGAAGAACCGAACTAGTAATAAATTTGGTCAAATTTAGGACAAGGAAGAAGCAGCCCGCGTGAACTCTCAAATCAAGATCCTCATCAGACCCATGTATTCCAATCCTCCTGTTAATGGATCACGGATTGTGAGCGAGATTCTTACCAACACTACTTTAAACAAGCAGTGGCTGGAAGATGTGAAGGGTATGGCTGACCGAATCATTACAATGAGACAAGAATTAAAGGATGGATTGGCCAAGGAAGGATCGAGCAAAAATTGGGAGCACATTGTGGATCAAATTGGAATGTTTTGTTTCACAGGAATGACCCCTGAGCAGGTATATTTGTTGTGATAAGGGAAAAGGTCTCCATACTTAACTGTTTTTCTGTCTTCAGGTTGAAAAAATTACCAAGGACTTTAGTGTTTACATGACCAAAGATGGCCGTATTTCCGTGGCAGGGATTTCCTCTTCTAATGTTGGATATTTGGCTAAAGCCATG

GOT2_SS7_A GGCCCCCCTGATCCTATTTTGGGCGTGAGCGAGGCCTTCAAAAAGGATACTAATCCCCTGAAAATGAATTTAGGGGTTGGTGCTTACCGAGATGATCAAGGCAAACCCTTTGTCTTGCCGTCTGTTCGTGAGGCCGAACGTATCATTGCTCAAAAGGGATTGAACAAAGAATACGCTCCAATTGGAGGTGAACCCGAGTTTGGCAGGCTATCCGCTAACTTAGCATTTGGCCAAGGTACGTAATCTCTGTCAAGGATGAATGGAAGAGCGTTCAGCTTAAATCAATG-AAAAAAATTGTATCATCTTTAGGAAATGAGATTGTTTCAAGTGGACGGAACGTGTCGGTTCAAACTATCTCTGGCACAGGTGCCTTGCGAGTAGGTGCCACTTATTTAGCCAAATGGTTCCCTGGGAACAAAACCGTGTATTTGCCCAAGCCTTCATGGGGCAATCATACCCCCATCTTTAAGCAAAGTGGGATGAATGTCGATGGTTATCGTTACTACGACCCTACAACATGTGGATTCGATTTTAATGGCGCCATGGAGGACATAAGCCGGATCCCTGAAAAAAGTGTTATTATGCTTCACGCTTGCGCTCATAACCCAACCGGAGTCGATCCCAAGGATGAGCAATGGAAAGAAATGTCACAATTGGTCAAGAAAAGGAATTTGTTTGCTTTCTTTGACATGGCTTATCAAGGCTTTGCTTCCGGCGACGTTGACGGAGATGCCTTTGCCGTCCGCCAATTTTTGGAAGATGGGCACAATATTTGTTTGTCCCAATCGTACGCCAAAAACATGGGTCTTTATGGCGAGCGAGTAGGAGCGTTTACTGTTGTTTGCAAAGTAAGTAGACGCGAAAAAAGGAAGAACCGAACTAGTAATAAATTTGGTCAAATTTAGGACAAGGAAGAAGCAGCCCGCGTGAACTCTCAAATCAAGATCCTCATCAGACCCATGTATTCCAATCCTCCTGTTAATGGATCACGGATTGTGAGCGAGATTCTTACCAACACTACTTTAAACAAGCAGTGGCTGGAAGATGTGAAGGGTATGGCTGACCGAATCATTACAATGAGACAAGAATTAAAGGATGGATTGGCCAAGGAAGGATCGAGCAAAAATTGGGAGCACATTGTGGATCAAATTGGAATGTTTTGTTTCACAGGAATGACCCCTGAGCAGGTATATTTGTTGTGATAAGGGAAAAGGTCTCCATACTTAACTGTTTTTCTGTCTTCAGGTTGAAAAAATTACCAAGGACTTTAGTGTTTACATGACCAAAGATGGCCGTATTTCCGTGGCAGGGATTTCCTCTTCTAATGTTGGATATTTGGCTAAAGCCATG

GOT2_SS7_B GGCCCCCCTGATCCTATTTTGGGCGTGAGCGAGGCCTTCAAAAAGGATACTAATCCCCTGAAAATGAATTTAGGGGTTGGTGCTTACCGAGATGATCAAGGCAAACCCTTTGTCTTGCCGTCTGTTCGTGAGGCCGAACGTATCATTGCTCAAAAGGGATTGAACAAAGAATACGCTCCAATTGGAGGTGAACCCGAGTTTGGCAGGCTATCCGCTAACTTAGCATTTGGCCAAGGTACGTAATCTCTGTCAAGGAAGAATGGAAGAGCGTTCAGCTTAAATCAATG-AAAAAAATTGTATCATCTTTAGGAAATGAGATTGTTTCAAGTGGACGGAACGTGTCGGTTCAAACTATCTCTGGCACAGGTGCCTTGCGAGTAGGTGCCACTTATTTAGCCAAATGGTTCCCTGGGAACAAAACCGTGTATTTGCCCAAGCCTTCATGGGGCAATCATACCCCCATCTTTAAGCAAAGTGGGATGAATGTCGATGGTTATCGTTACTACGACCCTACAACATGTGGATTCGATTTTAATGGCGCCATGGAGGACATAAGCCGGATCCCTGAAAAAAGTGTTATTATGCTTCACGCTTGCGCTCATAACCCAACCGGAGTCGATCCCAAGGATGAGCAATGGAAAGAAATGTCACAATTGGTCAAGAAAAGGAATTTGTTTGCTTTCTTTGACATGGCTTATCAAGGCTTTGCTTCCGGCGACGTTGACGGAGATGCCTTTGCCGTCCGCCAATTTTTGGAAGATGGGCACAATATTTGTTTGTCCCAATCGTACGCCAAAAACATGGGTCTTTATGGCGAGCGAGTAGGAGCGTTTACTGTTGTTTGCAAAGTAAGTAGACGCGAAAAAAGGAAGAACCGAACTAGTAATAAATTTGGTCAAATTTAGGACAAGGAAGAAGCAGCCCGCGTGAACTCTCAAATCAAGATCCTCATCAGACCCATGTATTCCAATCCTCCTGTTAATGGATCACGGATTGTGAGCGAGATTCTTACCAACACTACTTTAAACAAGCAGTGGCTGGAAGATGTGAAGGGTATGGCTGACCGAATCATTACAATGAGACAAGAATTAAAGGATGGATTGGCCAAGGAAGGATCGAGCAAAAATTGGGAGCACATTGTGGATCAAATTGGAATGTTTTGTTTCACAGGAATGACCCCTGAGCAGGTATATTTGTTGTGATAAGGGAAAAGGTCTCCATACTTAACTGTTTTTCTGTCTTCAGGTTGAAAAAATTACCAAGGACTTTAGTGTTTACATGACCAAAGATGGCCGTATTTCCGTGGCAGGGATTTCCTCTTCTAATGTTGGATATTTGGCTAAAGCCATG

AB_f2_GOT2a GGCCCCCCTGATCCTATTTTGGGCGTGAGCGAGGCCTTCAAAAAGGATACTAATCCCCTGAAAATGAATTTAGGGGTTGGTGCTTACCGGGATGATCAAGGCAAACCCTTTGTCTTGCCGTCTGTTCGTGAGGCCGAACGTATCATTGCTCAAAAAGGATTGAACAAAGAATACGCTCCAATTGGAGGTGAACCTGAGTTTGGCAAGCTATCCGCTAACTTAGCATTTGGCCAAGGTACGTAATCTCTGTCAAGGATGAATTGAGGAGCGTTCAGCTTAAATCAATG--AAAAAATGGTATCATCTTCAGGAAATGAGATTGTTTCAAGTGGACGGAACGTGTCGGTTCAGACTATCTCGGGCACAGGTGCCTTGCGAGTAGGTGCCACTTATTTAGCCAAATGGTTCCCTGGGAACAAAACCGTGTATTTGCCCAGGCCTTCATGGGGCAATCATACCCCCATCTTTAAGCAAAGTGGGATGAATGTCGATGGTTATCGTTACTACGACCCTTCAACTTGTGGATTCGATTTTAATGGCGCCATGGAGGATATAAGCCGGATCCCTGAAAAAAGCGTTATCATGCTTCACGCTTGCGCTCATAACCCAACCGGAGTCGATCCCAAGGATGAACAATGGAAAGAAATGTCACAATTGATCAAGAAAAGGAATCTGTTTGCTTTCTTTGACATGGCTTATCAAGGCTTTGCTTCCGGTGACGTTGACGGAGATGCCTTTGCCGTCCGCCAATTTTTGGAAGATGGGCACAATATTTGTTTGTCCCAATCGTACGCCAAAAACATGGGTCTTTATGGCGAGCGAGTAGGAGCGTTTACTGTTGTTTGCAAAGTAAGTATACGCGAAAAAAGGAAGAACCGACCTAGTAACATGTTTGGTTAAATTTAGGATAAGGAAGAAGCAGCCCGCGTGAACTCTCAAATCAAGATCCTCATCAGACCCATGTATTCCAATCCTCCTGTTAATGGATCACGAATTGTGAGCGAGATTCTTACCAACACTACTTTAAACAAGCAGTGGCTGGAAGATGTGAAGGGAATGGCTGACCGAATTATTACAATGAGACAGGAATTGAAGGATGGATTGGCCAAGGAAGGATCGAGCAAAAATTGGGAGCACATTGTGGATCAAATTGGAATGTTTTGTTTCACAGGAATGACTCCTGAACAGG--TATTTGTTGTGATAAAGGAAAAGGTCTCCATACTTAACTGTTTTTCTGTCTTTAGGTTGAAAAAATTACCAAGGACTTTAGTGTTTACATGACTAAAGATGGCCGTATTTCCGTGGCGGGGATTTCCTCATCTAATGTTGGATATTTGGCTAAAGCCATG

AB_f2_GOT2b GGCCCCCCTGATCCTATTTTGGGCGTGAGCGAGGCCTTCAAAAAGGATACTAATCCCCTGAAAATGAATTTAGGGGTTGGTGCTTACCGGGATGATCAAGGCAAACCCTTTGTCTTGCCGTCTGTTCGTGAGGCCGAACGTATCATTGCTCAAAAAGGATTGAACAAAGAATACGCTCCAATTGGAGGTGAACCTGAGTTTGGCAAGCTATCCGCTAACTTAGCATTTGGCCAAGGTACGTAATCTCTGTCAAGGATGAATTGAGGAGCGTTCAGCTTAAATCAATG--AAAAAATGGTATCATCTTCAGGAAATGAGATTGTTTCAAGTGGACGGAACGTGTCGGTTCAGACTATCTCGGGCACAGGTGCCTTGCGAGTAGGTGCCACTTATTTAGCCAAATGGTTCCCTGGGAACAAAACCGTGTATTTGCCCAGGCCTTCATGGGGCAATCATACCCCCATCTTTAAGCAAAGTGGGATGAATGTCGATGGTTATCGTTACTACGACCCTTCAACTTGTGGATTCGATTTTAATGGCGCCATGGAGGATATAAGCCGGATCCCTGAAAAAAGCGTTATCATGCTTCACGCTTGCGCTCATAACCCAACCGGAGTCGATCCCAAGGATGAACAATGGAAAGAAATGTCTCAATTGATCAAGAAAAGGAATCTGTTTGCTTTCTTTGACATGGCTTATCAAGGCTTTGCTTCCGGTGACGTTGACGGAGATGCCTTTGCCGTCCGCCAATTTTTGGAAGATGGGCACAATATTTGTTTGTCCCAATCGTACGCCAAAAACATGGGTCTTTATGGCGAGCGAGTAGGAGCGTTTACTGTTGTTTGCAAAGTAAGTATACGCGAAAAAAGGAAGAACCGACCTAGTAACATGTTTGGTTAAATTTAGGATAAGGAAGAAGCAGCCCGCGTGAACTCTCAAATCAAGATCCTCATCAGACCCATGTATTCCAATCCTCCTGTTAATGGATCACGAATTGTGAGCGAGATTCTTACCAACACTACTTTAAACAAGCAGTGGCTGGAAGATGTGAAGGGAATGGCTGACCGAATTATTACAATGAGACAGGAATTGAAGGATGGATTGGCCAAGGAAGGATCGAGCAAAAATTGGGAGCACATTGTGGATCAAATTGGAATGTTTTGTTTCACAGGAATGACTCCTGAACAGG--TATTTGTTGTGATAAAGGAAAAGGTCTCCATACTTAACTGTTTTTCTGTCTTTAGGTTGAAAAAATTACCAAGGACTTTAGTGTTTACATGACTAAAGATGGCCGTATTTCCGTGGCGGGGATTTCCTCATCTAATGTTGGATATTTGGCTAAAGCCATG

Ab_f9_GOT2a GGCCCCCCTGATCCTATTTTGGGCGTGAGCGAGGCCTTCAAAAAGGATACTAATCCCCTGAAAATGAATTTAGGGGTTGGTGCTTACCGGGATGATCAAGGCAAACCCTTTGTCTTGCCGTCTGTTCGTGAGGCCGAACGTATCATTGCTCAAAAAGGATTGAACAAAGAATACGCTCCAATTGGAGGTGAACCTGAGTTTGGCAAGCTATCCGCTAACTTAGCATTTGGCCAAGGTACGTAATCTCTGTCAAGGATGAATTGAGGAGCGTTCAGCTTAAATCAATG--AAAAAATGGTATCATCTTCAGGAAATGAGATTGTTTCAAGTGGACGGAACGTGTCGGTTCAGACTATCTCGGGCACAGGTGCCTTGCGAGTAGGTGCCACTTATTTAGCCAAATGGTTCCCTGGGAACAAAACCGTGTATTTGCCCAGGCCTTCATGGGGCAATCATACCCCCATCTTTAAGCAAAGTGGGATGAATGTCGATGGTTATCGTTACTACGACCCTTCAACTTGTGGATTCGATTTTAATGGCGCCATGGAGGATATAAGCCGGATCCCTGAAAAAAGCGTTATCATGCTTCACGCTTGCGCTCATAACCCAACCGGAGTCGATCCCAAGGATGAACAATGGAAAGAAATGTCACAATTGATCAAGAAAAGGAATCTGTTTGCTTTCTTTGACATGGCTTATCAAGGCTTTGCTTCCGGTGACGTTGACGGAGATGCCTTTGCCGTCCGCCAATTTTTGGAAGATGGGCACAATATTTGTTTGTCCCAATCGTACGCCAAAAACATGGGTCTTTATGGCGAGCGAGTAGGAGCGTTTACTGTTGTTTGCAAAGTAAGTATACGCGAAAAAAGGAAGAACCGACCTAGTAACATGTTTGGTTAAATTTAGGATAAGGAAGAAGCAGCCCGCGTGAACTCTCAAATCAAGATCCTCATCAGACCCATGTATTCCAATCCTCCTGTTAATGGATCACGAATTGTGAGCGAGATTCTTACCAACACTACTTTAAACAAGCAGTGGCTGGAAGATGTGAAGGGAATGGCTGACCGAATTATTACAATGAGACAGGAATTGAAGGATGGATTGGCCAAGGAAGGATCGAGCAAAAATTGGGAGCACATTGTGGATCAAATTGGAATGTTTTGTTTCACAGGAATGACTCCTGAACAGG--TATTTGTTGTGATAAAGGAAAAGGTCTCCATACTTAACTGTTTTTCTGTCTTTAGGTTGAAAAAATTACCAAGGACTTTAGTGTTTACATGACTAAAGATGGCCGTATTTCCGTGGCGGGGATTTCCTCATCTAATGTTGGATATTTGGCTAAAGCCATG

Ab_f9_GOT2b GGCCCCCCTGATCCTATTTTGGGCGTGAGCGAGGCCTTCAAAAAGGATACTAATCCCCTGAAAATGAATTTAGGGGTTGGTGCTTACCGGGATGATCAAGGCAAACCCTTTGTCTTGCCGTCTGTTCGTGAGGCCGAACGTATCATTGCTCAAAAAGGATTGAACAAAGAATACGCTCCAATTGGAGGTGAACCTGAGTTTGGCAAGCTATCCGCTAACTTAGCATTTGGCCAAGGTACGTAATCTCTGTCAAGGATGAATTGAGGAGCGTTCAGCTTAAATCAATG--AAAAAATGGTATCATCTTCAGGAAATGAGATTGTTTCAAGTGGACGGAACGTGTCGGTTCAGACTATCTCGGGCACAGGTGCCTTGCGAGTAGGTGCCACTTATTTAGCCAAATGGTTCCCTGGGAACAAAACCGTGTATTTGCCCAGGCCTTCATGGGGCAATCATACCCCCATCTTTAAGCAAAGTGGGATGAATGTCGATGGTTATCGTTACTACGACCCTTCAACTTGTGGATTCGATTTTAATGGCGCCATGGAGGATATAAGCCGGATCCCTGAAAAAAGCGTTATCATGCTTCACGCTTGCGCTCATAACCCAACCGGAGTCGATCCCAAGGATGAACAATGGAAAGAAATGTCACAATTGATCAAGAAAAGGAATCTGTTTGCTTTCTTTGACATGGCTTATCAAGGCTTTGCTTCCGGTGACGTTGACGGAGATGCCTTTGCCGTCCGCCAATTTTTGGAAGATGGGCACAATATTTGTTTGTCCCAATCGTACGCCAAAAACATGGGTCTTTATGGCGAGCGAGTAGGAGCGTTTACTGTTGTTTGCAAAGTAAGTATACGCGAAAAAAGGAAGAACCGACCTAGTAACATGTTTGGTTAAATTTAGGATAAGGAAGAAGCAGCCCGCGTGAACTCTCAAATCAAGATCCTCATCAGACCCATGTATTCCAATCCTCCTGTTAATGGATCACGAATTGTGAGCGAGATTCTTACCAACACTACTTTAAACAAGCAGTGGCTGGAAGATGTGAAGGGAATGGCTGACCGAATTATTACAATGAGACAGGAATTGAAGGATGGATTGGCCAAGGAAGGATCGAGCAAAAATTGGGAGCACATTGTGGATCAAATTGGAATGTTTTGTTTCACAGGAATGACTCCTGAACAGG--TATTTGTTGTGATAAAGGAAAAGGTCTCCATACTTAACTGTTTTTCTGTCTTTAGGTTGAAAAAATTACCAAGGACTTTAGTGTTTACATGACTAAAGATGGCCGTATTTCCGTGGCGGGGATTTCCTCATCTAATGTTGGATATTTGGCTAAAGCCATG

Ab_f10_GOT2a GGCCCCCCTGATCCTATTTTGGGCGTGAGCGAGGCCTTCAAAAAGGATACTAATCCCCTGAAAATGAATTTAGGGGTTGGTGCTTACCGGGATGATCAAGGCAAACCCTTTGTCTTGCCGTCTGTTCGTGAGGCCGAACGTATCATTGCTCAAAAAGGATTGAACAAAGAATACGCTCCAATTGGAGGTGAACCTGAGTTTGGCAAGCTATCCGCTAACTTAGCATTTGGCCAAGGTACGTAATCTCTGTCAAGGATGAATTGAGGAGCGTTCAGCTTAAATCAATG--AAAAAATGGTATCATCTTCAGGAAATGAGATTGTTTCAAGTGGACGGAACGTGTCGGTTCAGACTATCTCGGGCACAGGTGCCTTGCGAGTAGGTGCCACTTATTTAGCCAAATGGTTCCCTGGGAACAAAACCGTGTATTTGCCCAGGCCTTCATGGGGCAATCATACCCCCATCTTTAAGCAAAGTGGGATGAATGTCGATGGTTATCGTTACTACGACCCTTCAACTTGTGGATTCGATTTTAATGGCGCCATGGAGGATATAAGCCGGATCCCTGAAAAAAGCGTTATCATGCTTCACGCTTGCGCTCATAACCCAACCGGAGTCGATCCCAAGGATGAACAATGGAAAGAAATGTCACAATTGATCAAGAAAAGGAATCTGTTTGCTTTCTTTGACATGGCTTATCAAGGCTTTGCTTCCGGTGACGTTGACGGAGATGCCTTTGCCGTCCGCCAATTTTTGGAAGATGGGCACAATATTTGTTTGTCCCAATCGTACGCCAAAAACATGGGTCTTTATGGCGAGCGAGTAGGAGCGTTTACTGTTGTTTGCAAAGTAAGTATACGCGAAAAAAGGAAGAACCGACCTAGTAACATGTTTGGTTAAATTTAGGATAAGGAAGAAGCAGCCCGCGTGAACTCTCAAATCAAGATCCTCATCAGACCCATGTATTCCAATCCTCCTGTTAATGGATCACGAATTGTGAGCGAGATTCTTACCAACACTACTTTAAACAAGCAGTGGCTGGAAGATGTGAAGGGAATGGCTGACCGAATTATTACAATGAGACAGGAATTGAAGGATGGATTGGCCAAGGAAGGATCGAGCAAAAATTGGGAGCACATTGTGGATCAAATTGGAATGTTTTGTTTCACAGGAATGACTCCTGAACAGG--TATTTGTTGTGATAAAGGAAAAGGTCTCCATACTTAACTGTTTTTCTGTCTTTAGGTTGAAAAAATTACCAAGGACTTTAGTGTTTACATGACTAAAGATGGCCGTATTTCCGTGGCGGGGATTTCCTCATCTAATGTTGGATATTTGGCTAAAGCCATG

Ab_f10_GOT2b GGCCCCCCTGATCCTATTTTGGGCGTGAGCGAGGCCTTCAAAAAGGATACTAATCCCCTGAAAATGAATTTAGGGGTTGGTGCTTACCGGGATGATCAAGGCAAACCCTTTGTCTTGCCGTCTGTTCGTGAGGCCGAACGTATCATTGCTCAAAAAGGATTGAACAAAGAATACGCTCCAATTGGAGGTGAACCTGAGTTTGGCAAGCTATCCGCTAACTTAGCATTTGGCCAAGGTACGTAATCTCTGTCAAGGATGAATTGAGGAGCGTTCAGCTTAAATCAATG--AAAAAATGGTATCATCTTCAGGAAATGAGATTGTTTCAAGTGGACGGAACGTGTCGGTTCAGACTATCTCGGGCACAGGTGCCTTGCGAGTAGGTGCCACTTATTTAGCCAAATGGTTCCCTGGGAACAAAACCGTGTATTTGCCCAGGCCTTCATGGGGCAATCATACCCCCATCTTTAAGCAAAGTGGGATGAATGTCGATGGTTATCGTTACTACGACCCTTCAACTTGTGGATTCGATTTTAATGGCGCCATGGAGGATATAAGCCGGATCCCTGAAAAAAGCGTTATCATGCTTCACGCTTGCGCTCATAACCCAACCGGAGTCGATCCCAAGGATGAACAATGGAAAGAAATGTCACAATTGATCAAGAAAAGGAATCTGTTTGCTTTCTTTGACATGGCTTATCAAGGCTTTGCTTCCGGTGACGTTGACGGAGATGCCTTTGCCGTCCGCCAATTTTTGGAAGATGGGCACAATATTTGTTTGTCCCAATCGTACGCCAAAAACATGGGTCTTTATGGCGAGCGAGTAGGAGCGTTTACTGTTGTTTGCAAAGTAAGTATACGCGAAAAAAGGAAGAACCGACCTAGTAACATGTTTGGTTAAATTTAGGATAAGGAAGAAGCAGCCCGCGTGAACTCTCAAATCAAGATCCTCATCAGACCCATGTATTCCAATCCTCCTGTTAATGGATCACGAATTGTGAGCGAGATTCTTACCAACACTACTTTAAACAAGCAGTGGCTGGAAGATGTGAAGGGAATGGCTGACCGAATTATTACAATGAGACAGGAATTGAAGGATGGATTGGCCAAGGAAGGATCGAGCAAAAATTGGGAGCACATTGTGGATCAAATTGGAATGTTTTGTTTCACAGGAATGACTCCTGAACAGG--TATTTGTTGTGATAAAGGAAAAGGTCTCCATACTTAACTGTTTTTCTGTCTTTAGGTTGAAAAAATTACCAAGGACTTTAGTGTTTACATGACTAAAGATGGCCGTATTTCCGTGGCGGGGATTTCCTCATCTAATGTTGGATATTTGGCTAAAGCCATG

AB_f11_Got2a GGCCCCCCTGATCCTATTTTGGGCGTGAGCGAGGCCTTCAAAAAGGATACTAATCCCCTGAAAATGAATTTAGGGGTTGGTGCTTACCGGGATGATCAAGGCAAACCCTTTGTCTTGCCGTCTGTTCGTGAGGCCGAACGTATCATTGCTCAAAAAGGATTGAACAAAGAATACGCTCCAATTGGAGGTGAACCTGAGTTTGGCAAGCTATCCGCTAACTTAGCATTTGGCCAAGGTACGTAATCTCTGTCAAGGATGAATTGAGGAGCGTTCAGCTTAAATCAATG--AAAAAATGGTATCATCTTCAGGAAATGAGATTGTTTCAAGTGGACGGAACGTGTCGGTTCAGACTATCTCGGGCACAGGTGCCTTGCGAGTAGGTGCCACTTATTTAGCCAAATGGTTCCCTGGGAACAAAACCGTGTATTTGCCCAGGCCTTCATGGGGCAATCATACCCCCATCTTTAAGCAAAGTGGGATGAATGTCGATGGTTATCGTTACTACGACCCTTCAACTTGTGGATTCGATTTTAATGGCGCCATGGAGGATATAAGCCGGATCCCTGAAAAAAGCGTTATCATGCTTCACGCTTGCGCTCATAACCCAACCGGAGTCGATCCCAAGGATGAACAATGGAAAGAAATGTCACAATTGATCAAGAAAAGGAATCTGTTTGCTTTCTTTGACATGGCTTATCAAGGCTTTGCTTCCGGTGACGTTGACGGAGATGCCTTTGCCGTCCGCCAATTTTTGGAAGATGGGCACAATATTTGTTTGTCCCAATCGTACGCCAAAAACATGGGTCTTTATGGCGAGCGAGTAGGAGCGTTTACTGTTGTTTGCAAAGTAAGTATACGCGAAAAAAGGAAGAACCGACCTAGTAACATGTTTGGTTAAATTTAGGATAAGGAAGAAGCAGCCCGCGTGAACTCTCAAATCAAGATCCTCATCAGACCCATGTATTCCAATCCTCCTGTTAATGGATCACGAATTGTGAGCGAGATTCTTACCAACACTACTTTAAACAAGCAGTGGCTGGAAGATGTGAAGGGAATGGCTGACCGAATTATTACAATGAGACAGGAATTGAAGGATGGATTGGCCAAGGAAGGATCGAGCAAAAATTGGGAGCACATTGTGGATCAAATTGGAATGTTTTGTTTCACAGGAATGACTCCTGAACAGG--TATTTGTTGTGATAAAGGAAAAGGTCTCCATACTTAACTGTTTTTCTGTCTTTAGGTTGAAAAAATTACCAAGGACTTTAGTGTTTACATGACTAAAGATGGCCGTATTTCCGTGGCGGGGATTTCCTCATCTAATGTTGGATATTTGGCTAAAGCCATG

AB_f11_Got2b GGCCCCCCTGATCCTATTTTGGGCGTGAGCGAGGCCTTCAAAAAGGATACTAATCCCCTGAAAATGAATTTAGGGGTTGGTGCTTACCGGGATGATCAAGGCAAACCCTTTGTCTTGCCGTCTGTTCGTGAGGCCGAACGTATCATTGCTCAAAAAGGATTGAACAAAGAATACGCTCCAATTGGAGGTGAACCTGAGTTTGGCAAGCTATCCGCTAACTTAGCATTTGGCCAAGGTACGTAATCTCTGTCAAGGATGAATTGAGGAGCGTTCAGCTTAAATCAATG--AAAAAATGGTATCATCTTCAGGAAATGAGATTGTTTCAAGTGGACGGAACGTGTCGGTTCAGACTATCTCGGGCACAGGTGCCTTGCGAGTAGGTGCCACTTATTTAGCCAAATGGTTCCCTGGGAACAAAACCGTGTATTTGCCCAGGCCTTCATGGGGCAATCATACCCCCATCTTTAAGCAAAGTGGGATGAATGTCGATGGTTATCGTTACTACGACCCTTCAACTTGTGGATTCGATTTTAATGGCGCCATGGAGGATATAAGCCGGATCCCTGAAAAAAGCGTTATCATGCTTCACGCTTGCGCTCATAACCCAACCGGAGTCGATCCCAAGGATGAACAATGGAAAGAAATGTCACAATTGATCAAGAAAAGGAATCTGTTTGCTTTCTTTGACATGGCTTATCAAGGCTTTGCTTCCGGTGACGTTGACGGAGATGCCTTTGCCGTCCGCCAATTTTTGGAAGATGGGCACAATATTTGTTTGTCCCAATCGTACGCCAAAAACATGGGTCTTTATGGCGAGCGAGTAGGAGCGTTTACTGTTGTTTGCAAAGTAAGTATACGCGAAAAAAGGAAGAACCGACCTAGTAACATGTTTGGTTAAATTTAGGATAAGGAAGAAGCAGCCCGCGTGAACTCTCAAATCAAGATCCTCATCAGACCCATGTATTCCAATCCTCCTGTTAATGGATCACGAATTGTGAGCGAGATTCTTACCAACACTACTTTAAACAAGCAGTGGCTGGAAGATGTGAAGGGAATGGCTGACCGAATTATTACAATGAGACAGGAATTGAAGGATGGATTGGCCAAGGAAGGATCGAGCAAAAATTGGGAGCACATTGTGGATCAAATTGGAATGTTTTGTTTCACAGGAATGACTCCTGAACAGG--TATTTGTTGTGATAAAGGAAAAGGTCTCCATACTTAACTGTTTTTCTGTCTTTAGGTTGAAAAAATTACCAAGGACTTTAGTGTTTACATGACTAAAGATGGCCGTATTTCCGTGGCGGGGATTTCCTCATCTAATGTTGGATATTTGGCTAAAGCCATG

AB_f12_GOT2a GGCCCCCCTGATCCTATTTTGGGCGTGAGCGAGGCCTTCAAAAAGGATACTAATCCCCTGAAAATGAATTTAGGGGTTGGTGCTTACCGGGATGATCAAGGCAAACCCTTTGTCTTGCCGTCTGTTCGTGAGGCCGAACGTATCATTGCTCAAAAAGGATTGAACAAAGAATACGCTCCAATTGGAGGTGAACCTGAGTTTGGCAAGCTATCCGCTAACTTAGCATTTGGCCAAGGTACGTAATCTCTGTCAAGGATGAATTGAGGAGCGTTCAGCTTAAATCAATG--AAAAAATGGTATCATCTTCAGGAAATGAGATTGTTTCAAGTGGACGGAACGTGTCGGTTCAGACTATCTCGGGCACAGGTGCCTTGCGAGTAGGTGCCACTTATTTAGCCAAATGGTTCCCTGGGAACAAAACCGTGTATTTGCCCAGGCCTTCATGGGGCAATCATACCCCCATCTTTAAGCAAAGTGGGATGAATGTCGATGGTTATCGTTACTACGACCCTTCAACTTGTGGATTCGATTTTAATGGCGCCATGGAGGATATAAGCCGGATCCCTGAAAAAAGCGTTATCATGCTTCACGCTTGCGCTCATAACCCAACCGGAGTCGATCCCAAGGATGAACAATGGAAAGAAATGTCACAATTGATCAAGAAAAGGAATCTGTTTGCTTTCTTTGACATGGCTTATCAAGGCTTTGCTTCCGGTGACGTTGACGGAGATGCCTTTGCCGTCCGCCAATTTTTGGAAGATGGGCACAATATTTGTTTGTCCCAATCGTACGCCAAAAACATGGGTCTTTATGGCGAGCGAGTAGGAGCGTTTACTGTTGTTTGCAAAGTAAGTATACGCGAAAAAAGGAAGAACCGACCTAGTAACATGTTTGGTTAAATTTAGGATAAGGAAGAAGCAGCCCGCGTGAACTCTCAAATCAAGATCCTCATCAGACCCATGTATTCCAATCCTCCTGTTAATGGATCACGAATTGTGAGCGAGATTCTTACCAACACTACTTTAAACAAGCAGTGGCTGGAAGATGTGAAGGGAATGGCTGACCGAATTATTACAATGAGACAGGAATTGAAGGATGGATTGGCCAAGGAAGGATCGAGCAAAAATTGGGAGCACATTGTGGATCAAATTGGAATGTTTTGTTTCACAGGAATGACTCCTGAACAGG--TATTTGTTGTGATAAAGGAAAAGGTCTCCATACTTAACTGTTTTTCTGTCTTTAGGTTGAAAAAATTACCAAGGACTTTAGTGTTTACATGACTAAAGATGGCCGTATTTCCGTGGCGGGGATTTCCTCATCTAATGTTGGATATTTGGCTAAAGCCATG

AB_f12_GOT2b GGCCCCCCTGATCCTATTTTGGGCGTGAGCGAGGCCTTCAAAAAGGATACTAATCCCCTGAAAATGAATTTAGGGGTTGGTGCTTACCGGGATGATCAAGGCAAACCCTTTGTCTTGCCGTCTGTTCGTGAGGCCGAACGTATCATTGCTCAAAAAGGATTGAACAAAGAATACGCTCCAATTGGAGGTGAACCTGAGTTTGGCAAGCTATCCGCTAACTTAGCATTTGGCCAAGGTACGTAATCTCTGTCAAGGATGAATTGAGGAGCGTTCAGCTTAAATCAATG--AAAAAATGGTATCATCTTCAGGAAATGAGATTGTTTCAAGTGGACGGAACGTGTCGGTTCAGACTATCTCGGGCACAGGTGCCTTGCGAGTAGGTGCCACTTATTTAGCCAAATGGTTCCCTGGGAACAAAACCGTGTATTTGCCCAGGCCTTCATGGGGCAATCATACCCCCATCTTTAAGCAAAGTGGGATGAATGTCGATGGTTATCGTTACTACGACCCTTCAACTTGTGGATTCGATTTTAATGGCGCCATGGAGGATATAAGCCGGATCCCTGAAAAAAGCGTTATCATGCTTCACGCTTGCGCTCATAACCCAACCGGAGTCGATCCCAAGGATGAACAATGGAAAGAAATGTCACAATTGATCAAGAAAAGGAATCTGTTTGCTTTCTTTGACATGGCTTATCAAGGCTTTGCTTCCGGTGACGTTGACGGAGATGCCTTTGCCGTCCGCCAATTTTTGGAAGATGGGCACAATATTTGTTTGTCCCAATCGTACGCCAAAAACATGGGTCTTTATGGCGAGCGAGTAGGAGCGTTTACTGTTGTTTGCAAAGTAAGTATACGCGAAAAAAGGAAGAACCGACCTAGTAACATGTTTGGTTAAATTTAGGATAAGGAAGAAGCAGCCCGCGTGAACTCTCAAATCAAGATCCTCATCAGACCCATGTATTCCAATCCTCCTGTTAATGGATCACGAATTGTGAGCGAGATTCTTACCAACACTACTTTAAACAAGCAGTGGCTGGAAGATGTGAAGGGAATGGCTGACCGAATTATTACAATGAGACAGGAATTGAAGGATGGATTGGCCAAGGAAGGATCGAGCAAAAATTGGGAGCACATTGTGGATCAAATTGGAATGTTTTGTTTCACAGGAATGACTCCTGAACAGG--TATTTGTTGTGATAAAGGAAAAGGTCTCCATACTTAACTGTTTTTCTGTCTTTAGGTTGAAAAAATTACCAAGGACTTTAGTGTTTACATGACTAAAGATGGCCGTATTTCCGTGGCGGGGATTTCCTCATCTAATGTTGGATATTTGGCTAAAGCCATG

GOT2_BC3 GGCCCCCCTGATCCTATTTTGGGCGTGAGCGAGGCCTTCAAAAAGGATACTAATCCCCTGAAAATGAATTTAGGGGTCGGTGCTTACCGAGATGATCAAGGCAAACCCTTTGTCTTGCCGTCTGTTCGTGAAGCCGAACGTATCATTGCTCAAAAAGGATTGAACAAAGAATACGCTCCAATTGGAGGTGAACCCGAGTTTGGCAGGCTATCCGCTAACTTAGCATTTGGCCAAGGTACGTAATCTCTGTCAAGGATGAATTGAGGAGCGTTCAGCTTAAATCAATG-AAAAAAATGGTATCATCTTCAGGAAATGAGATTGTTTCAAGTGGACGGAACGTGTCGGTTCAGACTATCTCGGGCACAGGTGCCTTGCGAGTAGGTGCCACTTATTTAGCCAAATGGTTCCCTGGGAACAAAACCGTGTATTTGCCCAGGCCTTCATGGGGCAATCATACCCCCATCTTTAAGCAAAGTGGGATGAATGTCGATGGTTATCGTTACTACGACCCTACAACTTGTGGATTCGATTTTAATGGCGCCATGGAGGACATAAGCCGGATCCCTGAAAAAAGTGTTATCATGCTTCACGCTTGCGCTCACAACCCAACCGGAGTCGATCCCAAGGATGAACAATGGAAAGAAATGTCACAATTGGTCAAGAAAAGGAATTTGTTTGCTTTCTTTGACATGGCTTATCAAGGCTTTGCTTCCGGTGACGTTGACGGAGATGCCTTTGCCGTCCGCCAATTTTTGGAAGATGGGCACAATATTTGTTTGTCCCAATCGTACGCCAAAAACATGGGTCTTTATGGCGAGCGAGTAGGAGCGTTTACTGTTGTTTGCAAAGTAAGTATACGCGAAAAAAGGAAGAACCGAACTAGTAACATATTTGGTCAAATTTAGGACAAGGAAGAAGCAGCCCGCGTGAACTCTCAAATCAAGATCCTCATCAGACCCATGTATTCCAATCCTCCTGTTAATGGATCACGGATTGTGAGCGAGATTCTTACAAACACTACTTTAAACAAGCAGTGGCTGGAAGATGTGAAGGGTATGGCTGACCGAATCATTACAATGAGACAAGAATTAAAGGATGGATTGGCCAAGGAAGGATCGAGCAAAAATTGGGAGCACATTGTGGATCAAATTGGAATGTTTTGTTTCACAGGAATGACCCCTGAACAGG--TATTTGTTGTGATAATGGAAAAGGTCTCCATACTTAATTGTTTTTCTGTCTTCAGGTTGAAAAAATTACCAAGGACTTCAGTGTTTACATGACCAAAGATGGCCGTATTTCCGTGGCAGGGATTTCCTCTTCTAATGTTGGATATTTGGCTAAAGCCATG

GOT2_BC3_B GGCCCCCCTGATCCTATTTTGGGCGTGAGCGAGGCCTTCAAAAAGGATACTAATCCCCTGAAAATGAATTTAGGGGTCGGTGCTTACCGAGATGATCAAGGCAAACCCTTTGTCTTGCCGTCTGTTCGTGAAGCCGAACGTATCATTGCTCAAAAAGGATTGAACAAAGAATACGCTCCAATTGGAGGTGAACCCGAGTTTGGCAGGCTATCCGCTAACTTAGCATTTGGCCAAGGTACGTAATCTCTGTCAAGGATGAATTGAGGAGCGTTCAGCTTAAATCAATG-AAAAAAATGGTATCATCTTCAGGAAATGAGATTGTTTCAAGTGGACGGAACGTGTCGGTTCAGACTATCTCGGGCACAGGTGCCTTGCGAGTAGGTGCCACTTATTTAGCCAAATGGTTCCCTGGGAACAAAACCGTGTATTTGCCCAGGCCTTCATGGGGCAATCATACCCCCATCTTTAAGCAAAGTGGGATGAATGTCGATGGTTATCGTTACTACGACCCTACAACTTGTGGATTCGATTTTAATGGCGCCATGGAGGACATAAGCCGGATCCCTGAAAAAAGTGTTATCATGCTTCACGCTTGCGCTCACAACCCAACCGGAGTCGATCCCAAGGATGAACAATGGAAAGAAATGTCACAATTGGTCAAGAAAAGGAATTTGTTTGCTTTCTTTGACATGGCTTATCAAGGCTTTGCTTCCGGTGACGTTGACGGAGATGCCTTTGCCGTCCGCCAATTTTTGGAAGATGGGCACAATATTTGTTTGTCCCAATCGTACGCCAAAAACATGGGTCTTTATGGCGAGCGAGTAGGAGCGTTTACTGTTGTTTGCAAAGTAAGTATACGCGAAAAAAGGAAGAACCGAACTAGTAACATATTTGGTCAAATTTAGGACAAGGAAGAAGCAGCCCGCGTGAACTCTCAAATCAAGATCCTCATCAGACCCATGTATTCCAATCCTCCTGTTAATGGATCACGGATTGTGAGCGAGATTCTTACAAACACTACTTTAAACAAGCAGTGGCTGGAAGATGTGAAGGGTATGGCTGACCGAATCATTACAATGAGACAAGAATTAAAGGATGGATTGGCCAAGGAAGGATCGAGCAAAAATTGGGAGCACATTGTGGATCAAATTGGAATGTTTTGTTTCACAGGAATGACCCCTGAACAGG--TATTTGTTGTGATAATGGAAAAGGTCTCCATACTTAATTGTTTTTCTGTCTTCAGGTTGAAAAAATTACCAAGGACTTCAGTGTTTACATGACCAAAGATGGCCGTATTTCCGTGGCAGGGATTTCCTCTTCTAATGTTGGATATTTGGCTAAAGCCATG

GOT2_BC4 GGCCCCCCTGATCCTATTTTGGGCGTGAGCGAGGCCTTCAAAAAGGATACTAATCCCCTGAAAATGAATTTAGGGGTCGGTGCTTACCGAGATGATCAAGGCAAACCCTTTGTCTTGCCGTCTGTTCGTGAAGCCGAACGTATCATTGCTCAAAAAGGATTGAACAAAGAATACGCTCCAATTGGAGGTGAACCCGAGTTTGGCAGGCTATCCGCTAACTTAGCATTTGGCCAAGGTACGTAATCTCTGTCAAGGATGAATTGAGGAGCGTTCAGCTTAAATCAATG-AAAAAAATGGTATCATCTTCAGGAAATGAGATTGTTTCAAGTGGACGGAACGTGTCGGTTCAGACTATCTCGGGCACAGGTGCCTTGCGAGTAGGTGCCACTTATTTAGCCAAATGGTTCCCTGGGAACAAAACCGTGTATTTGCCCAGGCCTTCATGGGGCAATCATACCCCCATCTTTAAGCAAAGTGGGATGAATGTCGATGGTTATCGTTACTACGACCCTACAACTTGTGGATTCGATTTTAATGGCGCCATGGAGGACATAAGCCGGATCCCTGAAAAAAGTGTTATCATGCTTCACGCTTGCGCTCACAACCCAACCGGAGTCGATCCCAAGGATGAACAATGGAAAGAAATGTCACAATTGGTCAAGAAAAGGAATTTGTTTGCTTTCTTTGACATGGCTTATCAAGGCTTTGCTTCCGGTGACGTTGACGGAGATGCCTTTGCCGTCCGCCAATTTTTGGAAGATGGGCACAATATTTGTTTGTCCCAATCGTACGCCAAAAACATGGGTCTTTATGGCGAGCGAGTAGGAGCGTTTACTGTTGTTTGCAAAGTAAGTATACGCGAAAAAAGGAAGAACCGAACTAGTAACATATTTGGTCAAATTTAGGACAAGGAAGAAGCAGCCCGCGTGAACTCTCAAATCAAGATCCTCATCAGACCCATGTATTCCAATCCTCCTGTTAATGGATCACGGATTGTGAGCGAGATTCTTACAAACACTACTTTAAACAAGCAGTGGCTGGAAGATGTGAAGGGTATGGCTGACCGAATCATTACAATGAGACAAGAATTAAAGGATGGATTGGCCAAGGAAGGATCGAGCAAAAATTGGGAGCACATTGTGGATCAAATTGGAATGTTTTGTTTCACAGGAATGACCCCTGAACAGG--TATTTGTTGTGATAATGGAAAAGGTCTCCATACTTAATTGTTTTTCTGTCTTCAGGTTGAAAAAATTACCAAGGACTTCAGTGTTTACATGACCAAAGATGGCCGTATTTCCGTGGCAGGGATTTCCTCTTCTAATGTTGGATATTTGGCTAAAGCCATG

GOT2_BC4_B GGCCCCCCTGATCCTATTTTGGGCGTGAGCGAGGCCTTCAAAAAGGATACTAATCCCCTGAAAATGAATTTAGGGGTCGGTGCTTACCGAGATGATCAAGGCAAACCCTTTGTCTTGCCGTCTGTTCGTGAAGCCGAACGTATCATTGCTCAAAAAGGATTGAACAAAGAATACGCTCCAATTGGAGGTGAACCCGAGTTTGGCAGGCTATCCGCTAACTTAGCATTTGGCCAAGGTACGTAATCTCTGTCAAGGATGAATTGAGGAGCGTTCAGCTTAAATCAATG-AAAAAAATGGTATCATCTTCAGGAAATGAGATTGTTTCAAGTGGACGGAACGTGTCGGTTCAGACTATCTCGGGCACAGGTGCCTTGCGAGTAGGTGCCACTTATTTAGCCAAATGGTTCCCTGGGAACAAAACCGTGTATTTGCCCAGGCCTTCATGGGGCAATCATACCCCCATCTTTAAGCAAAGTGGGATGAATGTCGATGGTTATCGTTACTACGACCCTACAACTTGTGGATTCGATTTTAATGGCGCCATGGAGGACATAAGCCGGATCCCTGAAAAAAGTGTTATCATGCTTCACGCTTGCGCTCACAACCCAACCGGAGTCGATCCCAAGGATGAACAATGGAAAGAAATGTCACAATTGGTCAAGAAAAGGAATTTGTTTGCTTTCTTTGACATGGCTTATCAAGGCTTTGCTTCCGGTGACGTTGACGGAGATGCCTTTGCCGTCCGCCAATTTTTGGAAGATGGGCACAATATTTGTTTGTCCCAATCGTACGCCAAAAACATGGGTCTTTATGGCGAGCGAGTAGGAGCGTTTACTGTTGTTTGCAAAGTAAGTATACGCGAAAAAAGGAAGAACCGAACTAGTAACATATTTGGTCAAATTTAGGACAAGGAAGAAGCAGCCCGCGTGAACTCTCAAATCAAGATCCTCATCAGACCCATGTATTCCAATCCTCCTGTTAATGGATCACGGATTGTGAGCGAGATTCTTACAAACACTACTTTAAACAAGCAGTGGCTGGAAGATGTGAAGGGTATGGCTGACCGAATCATTACAATGAGACAAGAATTAAAGGATGGATTGGCCAAGGAAGGATCGAGCAAAAATTGGGAGCACATTGTGGATCAAATTGGAATGTTTTGTTTCACAGGAATGACCCCTGAACAGG--TATTTGTTGTGATAATGGAAAAGGTCTCCATACTTAATTGTTTTTCTGTCTTCAGGTTGAAAAAATTACCAAGGACTTCAGTGTTTACATGACCAAAGATGGCCGTATTTCCGTGGCAGGGATTTCCTCTTCTAATGTTGGATATTTGGCTAAAGCCATG

GOT2_BC7 GGCCCCCCTGATCCTATTTTGGGCGTGAGCGAGGCCTTCAAAAAGGATACTAATCCCCTGAAAATGAATTTAGGGGTCGGTGCTTACCGAGATGATCAAGGCAAACCCTTTGTCTTGCCGTCTGTTCGTGAAGCCGAACGTATCATTGCTCAAAAAGGATTGAACAAAGAATACGCTCCAATTGGAGGTGAACCCGAGTTTGGCAGGCTATCCGCTAACTTAGCATTTGGCCAAGGTACGTAATCTCTGTCAAGGATGAATTGAGGAGCGTTCAGCTTAAATCAATG-AAAAAAATGGTATCATCTTCAGGAAATGAGATTGTTTCAAGTGGACGGAACGTGTCGGTTCAGACTATCTCGGGCACAGGTGCCTTGCGAGTAGGTGCCACTTATTTAGCCAAATGGTTCCCTGGGAACAAAACCGTGTATTTGCCCAGGCCTTCATGGGGCAATCATACCCCCATCTTTAAGCAAAGTGGGATGAATGTCGATGGTTATCGTTACTACGACCCTACAACTTGTGGATTCGATTTTAATGGCGCCATGGAGGACATAAGCCGGATCCCTGAAAAAAGTGTTATCATGCTTCACGCTTGCGCTCACAACCCAACCGGAGTCGATCCCAAGGATGAACAATGGAAAGAAATGTCACAATTGGTCAAGAAAAGGAATTTGTTTGCTTTCTTTGACATGGCTTATCAAGGCTTTGCTTCCGGTGACGTTGACGGAGATGCCTTTGCCGTCCGCCAATTTTTGGAAGATGGGCACAATATTTGTTTGTCCCAATCGTACGCCAAAAACATGGGTCTTTATGGCGAGCGAGTAGGAGCGTTTACTGTTGTTTGCAAAGTAAGTATACGCGAAAAAAGGAAGAACCGAACTAGTAACATATTTGGTCAAATTTAGGACAAGGAAGAAGCAGCCCGCGTGAACTCTCAAATCAAGATCCTCATCAGACCCATGTATTCCAATCCTCCTGTTAATGGATCACGGATTGTGAGCGAGATTCTTACAAACACTACTTTAAACAAGCAGTGGCTGGAAGATGTGAAGGGTATGGCTGACCGAATCATTACAATGAGACAAGAATTAAAGGATGGATTGGCCAAGGAAGGATCGAGCAAAAATTGGGAGCACATTGTGGATCAAATTGGAATGTTTTGTTTCACAGGAATGACCCCTGAACAGG--TATTTGTTGTGATAATGGAAAAGGTCTCCATACTTAATTGTTTTTCTGTCTTCAGGTTGAAAAAATTACCAAGGACTTCAGTGTTTACATGACCAAAGATGGCCGTATTTCCGTGGCAGGGATTTCCTCTTCTAATGTTGGATATTTGGCTAAAGCCATG

GOT2_BC7_B GGCCCCCCTGATCCTATTTTGGGCGTGAGCGAGGCCTTCAAAAAGGATACTAATCCCCTGAAAATGAATTTAGGGGTCGGTGCTTACCGAGATGATCAAGGCAAACCCTTTGTCTTGCCGTCTGTTCGTGAAGCCGAACGTATCATTGCTCAAAAAGGATTGAACAAAGAATACGCTCCAATTGGAGGTGAACCCGAGTTTGGCAGGCTATCCGCTAACTTAGCATTTGGCCAAGGTACGTAATCTCTGTCAAGGATGAATTGAGGAGCGTTCAGCTTAAATCAATG-AAAAAAATGGTATCATCTTCAGGAAATGAGATTGTTTCAAGTGGACGGAACGTGTCGGTTCAGACTATCTCGGGCACAGGTGCCTTGCGAGTAGGTGCCACTTATTTAGCCAAATGGTTCCCTGGGAACAAAACCGTGTATTTGCCCAGGCCTTCATGGGGCAATCATACCCCCATCTTTAAGCAAAGTGGGATGAATGTCGATGGTTATCGTTACTACGACCCTACAACTTGTGGATTCGATTTTAATGGCGCCATGGAGGACATAAGCCGGATCCCTGAAAAAAGTGTTATCATGCTTCACGCTTGCGCTCACAACCCAACCGGAGTCGATCCCAAGGATGAACAATGGAAAGAAATGTCACAATTGGTCAAGAAAAGGAATTTGTTTGCTTTCTTTGACATGGCTTATCAAGGCTTTGCTTCCGGTGACGTTGACGGAGATGCCTTTGCCGTCCGCCAATTTTTGGAAGATGGGCACAATATTTGTTTGTCCCAATCGTACGCCAAAAACATGGGTCTTTATGGCGAGCGAGTAGGAGCGTTTACTGTTGTTTGCAAAGTAAGTATACGCGAAAAAAGGAAGAACCGAACTAGTAACATATTTGGTCAAATTTAGGACAAGGAAGAAGCAGCCCGCGTGAACTCTCAAATCAAGATCCTCATCAGACCCATGTATTCCAATCCTCCTGTTAATGGATCACGGATTGTGAGCGAGATTCTTACAAACACTACTTTAAACAAGCAGTGGCTGGAAGATGTGAAGGGTATGGCTGACCGAATCATTACAATGAGACAAGAATTAAAGGATGGATTGGCCAAGGAAGGATCGAGCAAAAATTGGGAGCACATTGTGGATCAAATTGGAATGTTTTGTTTCACAGGAATGACCCCTGAACAGG--TATTTGTTGTGATAATGGAAAAGGTCTCCATACTTAATTGTTTTTCTGTCTTCAGGTTGAAAAAATTACCAAGGACTTCAGTGTTTACATGACCAAAGATGGCCGTATTTCCGTGGCAGGGATTTCCTCTTCTAATGTTGGATATTTGGCTAAAGCCATG

GOT2_BC8 GGCCCCCCTGATCCTATTTTGGGCGTGAGCGAGGCCTTCAAAAAGGATACTAATCCCCTGAAAATGAATTTAGGGGTCGGTGCTTACCGAGATGATCAAGGCAAACCCTTTGTCTTGCCGTCTGTTCGTGAAGCCGAACGTATCATTGCTCAAAAAGGATTGAACAAAGAATACGCTCCAATTGGAGGTGAACCCGAGTTTGGCAGGCTATCCGCTAACTTAGCATTTGGCCAAGGTACGTAATCTCTGTCAAGGATGAATTGAGGAGCGTTCAGCTTAAATCAATG-AAAAAAATGGTATCATCTTCAGGAAATGAGATTGTTTCAAGTGGACGGAACGTGTCGGTTCAGACTATCTCGGGCACAGGTGCCTTGCGAGTAGGTGCCACTTATTTAGCCAAATGGTTCCCTGGGAACAAAACCGTGTATTTGCCCAGGCCTTCATGGGGCAATCATACCCCCATCTTTAAGCAAAGTGGGATGAATGTCGATGGTTATCGTTACTACGACCCTACAACTTGTGGATTCGATTTTAATGGCGCCATGGAGGACATAAGCCGGATCCCTGAAAAAAGTGTTATCATGCTTCACGCTTGCGCTCACAACCCAACCGGAGTCGATCCCAAGGATGAACAATGGAAAGAAATGTCACAATTGGTCAAGAAAAGGAATTTGTTTGCTTTCTTTGACATGGCTTATCAAGGCTTTGCTTCCGGTGACGTTGACGGAGATGCCTTTGCCGTCCGCCAATTTTTGGAAGATGGGCACAATATTTGTTTGTCCCAATCGTACGCCAAAAACATGGGTCTTTATGGCGAGCGAGTAGGAGCGTTTACTGTTGTTTGCAAAGTAAGTATACGCGAAAAAAGGAAGAACCGAACTAGTAACATATTTGGTCAAATTTAGGACAAGGAAGAAGCAGCCCGCGTGAACTCTCAAATCAAGATCCTCATCAGACCCATGTATTCCAATCCTCCTGTTAATGGATCACGGATTGTGAGCGAGATTCTTACAAACACTACTTTAAACAAGCAGTGGCTGGAAGATGTGAAGGGTATGGCTGACCGAATCATTACAATGAGACAAGAATTAAAGGATGGATTGGCCAAGGAAGGATCGAGCAAAAATTGGGAGCACATTGTGGATCAAATTGGAATGTTTTGTTTCACAGGAATGACCCCTGAACAGG--TATTTGTTGTGATAATGGAAAAGGTCTCCATACTTAATTGTTTTTCTGTCTTCAGGTTGAAAAAATTACCAAGGACTTCAGTGTTTACATGACCAAAGATGGCCGTATTTCCGTGGCAGGGATTTCCTCTTCTAATGTTGGATATTTGGCTAAAGCCATG

GOT2_BC8_B GGCCCCCCTGATCCTATTTTGGGCGTGAGCGAGGCCTTCAAAAAGGATACTAATCCCCTGAAAATGAATTTAGGGGTCGGTGCTTACCGAGATGATCAAGGCAAACCCTTTGTCTTGCCGTCTGTTCGTGAAGCCGAACGTATCATTGCTCAAAAAGGATTGAACAAAGAATACGCTCCAATTGGAGGTGAACCCGAGTTTGGCAGGCTATCCGCTAACTTAGCATTTGGCCAAGGTACGTAATCTCTGTCAAGGATGAATTGAGGAGCGTTCAGCTTAAATCAATG-AAAAAAATGGTATCATCTTCAGGAAATGAGATTGTTTCAAGTGGACGGAACGTGTCGGTTCAGACTATCTCGGGCACAGGTGCCTTGCGAGTAGGTGCCACTTATTTAGCCAAATGGTTCCCTGGGAACAAAACCGTGTATTTGCCCAGGCCTTCATGGGGCAATCATACCCCCATCTTTAAGCAAAGTGGGATGAATGTCGATGGTTATCGTTACTACGACCCTACAACTTGTGGATTCGATTTTAATGGCGCCATGGAGGACATAAGCCGGATCCCTGAAAAAAGTGTTATCATGCTTCACGCTTGCGCTCACAACCCAACCGGAGTCGATCCCAAGGATGAACAATGGAAAGAAATGTCACAATTGGTCAAGAAAAGGAATTTGTTTGCTTTCTTTGACATGGCTTATCAAGGCTTTGCTTCCGGTGACGTTGACGGAGATGCCTTTGCCGTCCGCCAATTTTTGGAAGATGGGCACAATATTTGTTTGTCCCAATCGTACGCCAAAAACATGGGTCTTTATGGCGAGCGAGTAGGAGCGTTTACTGTTGTTTGCAAAGTAAGTATACGCGAAAAAAGGAAGAACCGAACTAGTAACATATTTGGTCAAATTTAGGACAAGGAAGAAGCAGCCCGCGTGAACTCTCAAATCAAGATCCTCATCAGACCCATGTATTCCAATCCTCCTGTTAATGGATCACGGATTGTGAGCGAGATTCTTACAAACACTACTTTAAACAAGCAGTGGCTGGAAGATGTGAAGGGTATGGCTGACCGAATCATTACAATGAGACAAGAATTAAAGGATGGATTGGCCAAGGAAGGATCGAGCAAAAATTGGGAGCACATTGTGGATCAAATTGGAATGTTTTGTTTCACAGGAATGACCCCTGAACAGG--TATTTGTTGTGATAATGGAAAAGGTCTCCATACTTAATTGTTTTTCTGTCTTCAGGTTGAAAAAATTACCAAGGACTTCAGTGTTTACATGACCAAAGATGGCCGTATTTCCGTGGCAGGGATTTCCTCTTCTAATGTTGGATATTTGGCTAAAGCCATG

GOT2_BH1f_A GGCCCCCCTGATCCTATTTTGGGCGTGAGCGAGGCCTTCAAAAAGGATACTAATCCCCTGAAAATGAATTTAGGGGTCGGTGCTTACCGAGATGATCAAGGCAAACCCTTTGTCTTGCCGTCTGTTCGTGAGGCCGAACGTATCATTGCTCAAAAAGGATTGAACAAAGAATACGCTCCAATTGGAGGTGAACCCGAGTTTGGCAGGCTATCCGCTAACTTAGCATTTGGCCAAGGTACGTAATCTCTGTCAAGGATGAATTGAGGAGCGTTCAGCTTAAATCAATG-AAAAAAATGGTATCATCTTCAGGAAATGAGATTGTTTCAAGTGGACGGAACGTGTCGGTTCAGACTATCTCGGGCACAGGTGCCTTGCGAGTAGGTGCCACTTATTTAGCCAAATGGTTCCCTGGGAACAAAACCGTGTATTTGCCCAGGCCTTCATGGGGCAATCATACCCCCATCTTTAAGCAAAGTGGGATGAATGTCGATGGTTATCGTTACTACGACCCTACAACTTGTGGATTCGATTTTAATGGCGCCATGGAGGACATAAGCCGGATCCCTGAAAAAAGTGTTATCATGCTTCACGCTTGCGCTCACAACCCAACCGGAGTCGATCCCAAGGATGAACAATGGAAAGAAATGTCACAATTGGTCAAGAAAAGGAATTTGTTTGCTTTCTTTGACATGGCTTATCAAGGCTTTGCTTCCGGTGACGTTGACGAAGATGCCTTTGCCGTCCGCCAATTTTTGGAAGATGGGCACAATATTTGTTTGTCCCAATCGTACGCCAAAAACATGGGTCTTTATGGCGAGCGAGTAGGAGCGTTTACTGTTGTTTGCAAAGTAAGTATACGCGAAAAAAGGAAGAACCGAACTAGTAACATATTTGGTCAAATTTAGGACAAGGAAGAAGCAGCCCGCGTGAACTCTCAAATCAAGATCCTCATCAGACCCATGTATTCCAATCCTCCTGTTAATGGATCACGGATTGTGAGCGAGATTCTTACAAACACTACTTTAAACAAGCAGTGGCTGGAAGATGTGAAGGGTATGGCTGACCGAATCATTACAATGAGACAAGAATTAAAGGATGGATTGGCCAAGGAAGGATCGAGCAAAAATTGGGAGCACATTGTGGATCAAATTGGAATGTTTTGTTTCACAGGAATGACCCCTGAACAGG--TATTTGTTGTGATAATGGAAAAGGTCTCCATACTTAACTGTTTTTCTGTCTTCAGGTTGAAAAAATTACCAAGGACTTCAGTGTTTACATGACCAAAGATGGCCGTATTTCCGTGGCAGGGATTTCCTCTTCTAATGTTGGATATTTGGCTAAAGCCATG

GOT2_BH1f_B GGCCCCCCTGATCCTATTTTGGGCGTGAGCGAGGCCTTCAAAAAGGATACTAATCCCCTGAAAATGAATTTAGGAGTCGGTGCTTACCGAGATGATCAAGGCAAACCCTTTGTCTTGCCGTCTGTTCGTGAGGCCGAACGTATCATTGCTCAAAAAGGATTGAACAAAGAATACGCTCCAATTGGAGGTGAACCCGAGTTTGGCAGGCTATCCGCTAACTTAGCATTTGGCCAAGGTACGTAATCTCTGTCAAGGATGAATTGAGGAGCGTTCAGCTTAAATCAATG-AAAAAAATGGTATCATCTTCAGGAAATGAGATTGTTTCAAGTGGACGGAACGTGTCGGTTCAGACTATCTCGGGCACAGGTGCCTTGCGAGTAGGTGCCACTTATTTAGCCAAATGGTTCCCTGGGAACAAAACCGTGTATTTGCCCAGGCCTTCATGGGGCAATCATACCCCCATCTTTAAGCAAAGTGGGATGAATGTCGATGGTTATCGTTACTACGACCCTACAACTTGTGGATTCGATTTTAATGGCGCCATGGAGGACATAAGCCGGATCCCTGAAAAAAGTGTTATCATGCTTCACGCTTGCGCTCACAACCCAACCGGAGTCGATCCCAAGGATGAACAATGGAAAGAAATGTCACAATTGGTCAAGAAAAGGAATTTGTTTGCTTTCTTTGACATGGCTTATCAAGGCTTTGCTTCCGGTGACGTTGACGAAGATGCCTTTGCCGTCCGCCAATTTTTGGAAGATGGGCACAATATTTGTTTGTCCCAATCGTACGCCAAAAACATGGGTCTTTATGGCGAGCGAGTAGGAGCGTTTACTGTTGTTTGCAAAGTAAGTATACGCGAAAAAAGGAAGAACCGAACTAGTAACATATTTGGTCAAATTTAGGACAAGGAAGAAGCAGCCCGCGTGAACTCTCAAATCAAGATCCTCATCAGACCCATGTATTCCAATCCTCCTGTTAATGGATCACGGATTGTGAGCGAGATTCTTACAAACACTACTTTAAACAAGCAGTGGCTGGAAGATGTGAAGGGTATGGCTGACCGAATCATTACAATGAGACAAGAATTAAAGGATGGATTGGCCAAGGAAGGATCGAGCAAAAATTGGGAGCACATTGTGGATCAAATTGGAATGTTTTGTTTCACAGGAATGACCCCTGAACAGG--TATTTGTTGTGATAATGGAAAAGGTCTCCATACTTAACTGTTTTTCTGTCTTCAGGTTGAAAAAATTACCAAGGACTTCAGTGTTTACATGACCAAAGATGGCCGTATTTCCGTGGCAGGGATTTCCTCTTCTAATGTTGGATATTTGGCTAAAGCCATG

GOT2_BH2f_A GGCCCCCCTGATCCTATTTTGGGCGTGAGCGAGGCCTTCAAAAAGGATACTAATCCCCTGAAAATGAATTTAGGAGTCGGTGCTTACCGAGATGATCAAGGCAAACCCTTTGTCTTGCCGTCTGTTCGTGAGGCCGAACGTATCATTGCTCAAAAAGGATTGAACAAAGAATACGCTCCAATTGGAGGTGAACCCGAGTTTGGCAGGCTATCCGCTAACTTAGCATTTGGCCAAGGTACGTAATCTCTGTCAAGGATGAATTGAGGAGCGTTCAGCTTAAATCAATG-AAAAAAATGGTATCATCTTCAGGAAATGAGATTGTTTCAAGTGGACGGAACGTGTCGGTTCAGACTATCTCGGGCACAGGTGCCTTGCGAGTAGGTGCCACTTATTTAGCCAAATGGTTCCCTGGGAACAAAACCGTGTATTTGCCCAGGCCTTCATGGGGCAATCATACCCCCATCTTTAAGCAAAGTGGGATGAATGTCGATGGTTATCGTTACTACGACCCTACAACTTGTGGATTCGATTTTAATGGCGCCATGGAGGACATAAGCCGGATCCCTGAAAAAAGTGTTATCATGCTTCACGCTTGCGCTCACAACCCAACCGGAGTCGATCCCAAGGATGAACAATGGAAAGAAATGTCACAATTGGTCAAGAAAAGGAATTTGTTTGCTTTCTTTGACATGGCTTATCAAGGCTTTGCTTCCGGTNACNNNNNNNNNNNNNNNNNNNNNNNNNNCCAATTTTTGGNAGATGGGCACAATATTTGTTTGTCCCAATCGTACGCCAAAAACATGGGTCTTTATGGCGAGCGAGTAGGAGCGTTTACTGTTGTTTGCAAAGTAAGTATACGCGAAAAAAGGAAGAACCGAACTAGTAACATATTTGGTCAAATTTAGGACAAGGAAGAAGCAGCCCGCGTGAACTCTCAAATCAAGATCCTCATCAGACCCATGTATTCCAATCCTCCTGTTAATGGATCACGGATTGTGAGCGAGATTCTTACAAACACTACTTTAAACAAGCAGTGGCTGGAAGATGTGAAGGGTATGGCTGACCGAATCATTACAATGAGACAAGAATTAAAGGATGGATTGGCCAAGGAAGGATCGAGCAAAAATTGGGAGCACATTGTGGATCAAATTGGAATGTTTTGTTTCACAGGAATGACCCCTGAACAGG--TATTTGTTGTGATAATGGAAAAGGTCTCCATACTTAATTGTTTTTCTGTCCTCCGGTTGAAAAAATTACCAAGGACTTCAGTGTTTACATGACCAAAGATGGCCGTATTTCCGTGGCAGGGATTTCCTCTTCTAATGTTGGATATTTGGCTAAAGCCATG

GOT2_BH2f_B GGCCCCCCTGATCCTATTTTGGGCGTGAGCGAGGCCTTCAAAAAGGATACTAATCCCCTGAAAATGAATTTAGGGGTCGGTGCTTACCGAGATGATCAAGGCAAACCCTTTGTCTTGCCGTCTGTTCGTGAGGCCGAACGTATCATTGCTCAAAAAGGATTGAACAAAGAATACGCTCCAATTGGAGGTGAACCCGAGTTTGGCAGGCTATCCGCTAACTTAGCATTTGGCCAAGGTACGTAATCTCTGTCAAGGATGAATTGAGGAGCGTTCAGCTTAAATCAATG-AAAAAAATGGTATCATCTTCAGGAAATGAGATTGTTTCAAGTGGACGGAACGTGTCGGTTCAGACTATCTCGGGCACAGGTGCCTTGCGAGTAGGTGCCACTTATTTAGCCAAATGGTTCCCTGGGAACAAAACCGTGTATTTGCCCAGGCCTTCATGGGGCAATCATACCCCCATCTTTAAGCAAAGTGGGATGAATGTCGATGGTTATCGTTACTACGACCCTACAACTTGTGGATTCGATTTTAATGGCGCCATGGAGGACATAAGCCGGATCCCTGAAAAAAGTGTTATCATGCTTCACGCTTGCGCTCACAACCCAACCGGAGTCGATCCCAAGGATGAACAATGGAAAGAAATGTCACAATTGGTCAAGAAAAGGAATTTGTTTGCTTTCTTTGACATGGCTTATCAAGGCTTTGCTTCCGGTNACNNNNNNNNNNNNNNNNNNNNNNNNNNCCAATTTTTGGNAGATGGGCACAATATTTGTTTGTCCCAATCGTACGCCAAAAACATGGGTCTTTATGGCGAGCGAGTAGGAGCGTTTACTGTTGTTTGCAAAGTAAGTATACGCGAAAAAAGGAAGAACCGAACTAGTAACATATTTGGTCAAATTTAGGACAAGGAAGAAGCAGCCCGCGTGAACTCTCAAATCAAGATCCTCATCAGACCCATGTATTCCAATCCTCCTGTTAATGGATCACGGATTGTGAGCGAGATTCTTACAAACACTACTTTAAACAAGCAGTGGCTGGAAGATGTGAAGGGTATGGCTGACCGAATCATTACAATGAGACAAGAATTAAAGGATGGATTGGCCAAGGAAGGATCGAGCAAAAATTGGGAGCACATTGTGGATCAAATTGGAATGTTTTGTTTCACAGGAATGACCCCTGAACAGG--TATTTGTTGTGATAATGGAAAAGGTCTCCATACTTAACTGTTTTTCTGTCTTCAGGTTGAAAAAATTACCAAGGACTTCAGTGTTTACATGACCAAAGATGGCCGTATTTCCGTGGCAGGGATTTCCTCTTCTAATGTTGGATATTTGGCTAAAGCCATG

GOT2_BH3f_A GGCCCCCCTGATCCTATTTTGGGCGTGAGCGAGGCCTTCAAAAAGGATACTAATCCCCTGAAAATGAATTTAGGGGTCGGTGCTTACCGAGATGATCAAGGCAAACCCTTTGTCTTGCCGTCTGTTCGTGAGGCCGAACGTATCATTGCTCAAAAAGGATTGAACAAAGAATACGCTCCAATTGGAGGTGAACCCGAGTTTGGCAGGCTATCCGCTAACTTAGCATTTGGCCAAGGTACGTAATCTCTGTCAAGGATGAATTGAGGAGCGTTCAGCTTAAATCAATG-AAAAAAATGGTATCATCTTCAGGAAATGAGATTGTTTCAAGTGGACGGAACGTGTCGGTTCAGACTATCTCGGGCACAGGTGCCTTGCGAGTAGGTGCCACTTATTTAGCCAAATGGTTCCCTGGGAACAAAACCGTGTATTTGCCCAGGCCTTCATGGGGCAATCATACCCCCATCTTTAAGCAAAGTGGGATGAATGTCGATGGTTATCGTTACTACGACCCTACAACTTGTGGATTCGATTTTAATGGCGCCATGGAGGACATAAGCCGGATCCCTGAAAAAAGTGTTATCATGCTTCACGCTTGCGCTCACAACCCAACCGGAGTCGATCCCAAGGATGAACAATGGAAAGAAATGTCACAATTGGTCAAGAAAAGGAATTTGTTTGCTTTCTTTGACATGGCTTATCAAGGCTTTGCTTCCGGTGACGTTGACGAANATGCCTTTGCCGTCCGCCAATTTTTGGAAGATGGGCACAATATTTGTTTGTCCCAATCGTACGCCAAAAACATGGGTCTTTATGGCGAGCGAGTAGGAGCGTTTACTGTTGTTTGCAAAGTAAGTATACGCGAAAAAAGGAAGAACCGAACTAGTAACATATTTGGTCAAATTTAGGACAAGGAAGAAGCAGCCCGCGTGAACTCTCAAATCAAGATCCTCATCAGACCCATGTATTCCAATCCTCCTGTTAATGGATCACGGATTGTGAGCGAGATTCTTACAAACACTACTTTAAACAAGCAGTGGCTGGAAGATGTGAAGGGTATGGCTGACCGAATCATTACAATGAGACAAGAATTAAAGGATGGATTGGCCAAGGAAGGATCGAGCAAAAATTGGGAGCACATTGTGGATCAAATTGGAATGTTTTGTTTCACAGGAATGACCCCTGAACAGG--TATTTGTTGTGATAATGGAAAAGGTCTCCATACTTAACTGTTTTTCTGTCTTCAGGTTGAAAAAATTACCAAGGACTTCAGTGTTTACATGACCAAAGATGGCCGTATTTCCGTGGCAGGGATTTCCTCTTCTAATGTTGGATATTTGGCTAAAGCCATG

GOT2_BH3f_B GGCCCCCCTGATCCTATTTTGGGCGTGAGCGAGGCCTTCAAAAAGGATACTAATCCCCTGAAAATGAATTTAGGGGTCGGTGCTTACCGAGATGATCAAGGCAAACCCTTTGTCTTGCCGTCTGTTCGTGAGGCCGAACGTATCATTGCTCAAAAAGGATTGAACAAAGAATACGCTCCAATTGGAGGTGAACCCGAGTTTGGCAGGCTATCCGCTAACTTAGCATTTGGCCAAGGTACGTAATCTCTGTCAAGGATGAATTGAGGAGCGTTCAGCTTAAATCAATG-AAAAAAATGGTATCATCTTCAGGAAATGAGATTGTTTCAAGTGGACGGAACGTGTCGGTTCAGACTATCTCGGGCACAGGTGCCTTGCGAGTAGGTGCCACTTATTTAGCCAAATGGTTCCCTGGGAACAAAACCGTGTATTTGCCCAGGCCTTCATGGGGCAATCATACCCCCATCTTTAAGCAAAGTGGGATGAATGTCGATGGTTATCGTTACTACGACCCTACAACTTGTGGATTCGATTTTAATGGCGCCATGGAGGACATAAGCCGGATCCCTGAAAAAAGTGTTATCATGCTTCACGCTTGCGCTCACAACCCAACCGGAGTCGATCCCAAGGATGAACAATGGAAAGAAATGTCACAATTGGTCAAGAAAAGGAATTTGTTTGCTTTCTTTGACATGGCTTATCAAGGCTTTGCTTCCGGTGACGTTGACGAANATGCCTTTGCCGTCCGCCAATTTTTGGAAGATGGGCACAATATTTGTTTGTCCCAATCGTACGCCAAAAACATGGGTCTTTATGGCGAGCGAGTAGGAGCGTTTACTGTTGTTTGCAAAGTAAGTATACGCGAAAAAAGGAAGAACCGAACTAGTAACATATTTGGTCAAATTTAGGACAAGGAAGAAGCAGCCCGCGTGAACTCTCAAATCAAGATCCTCATCAGACCCATGTATTCCAATCCTCCTGTTAATGGATCACGGATTGTGAGCGAGATTCTTACAAACACTACTTTAAACAAGCAGTGGCTGGAAGATGTGAAGGGTATGGCTGACCGAATCATTACAATGAGACAAGAATTAAAGGATGGATTGGCCAAGGAAGGATCGAGCAAAAATTGGGAGCACATTGTGGATCAAATTGGAATGTTTTGTTTCACAGGAATGACCCCTGAACAGG--TATTTGTTGTGATAATGGAAAAGGTCTCCATACTTAACTGTTTTTCTGTCTTCAGGTTGAAAAAATTACCAAGGACTTCAGTGTTTACATGACCAAAGATGGCCGTATTTCCGTGGCAGGGATTTCCTCTTCTAATGTTGGATATTTGGCTAAAGCCATG

GOT2_BH7f_A GGCCCCCCTGATCCTATTTTGGGCGTGAGCGAGGCCTTCAAAAAGGATACTAATCCCCTGAAAATGAATTTAGGGGTCGGTGCTTACCGAGATGATCAAGGCAAACCCTTTGTCTTGCCGTCTGTTCGTGAGGCCGAACGTATCATTGCTCAAAAAGGATTGAACAAAGAATACGCTCCAATTGGAGGTGAACCCGAGTTTGGCAGGCTATCCGCTAACTTAGCATTTGGCCAAGGTACGTAATCTCTGTCAAGGATGAATTGAGGAGCGTTCAGCTTAAATCAATG-AAAAAAATGGTATCATCTTCAGGAAATGAGATTGTTTCAAGTGGACGGAACGTGTCGGTTCAGACTATCTCGGGCACAGGTGCCTTGCGAGTAGGTGCCACTTATTTAGCCAAATGGTTCCCTGGGAACAAAACCGTGTATTTGCCCAGGCCTTCATGGGGCAATCATACCCCCATCTTTAAGCAAAGTGGGATGAATGTCGATGGTTATCGTTACTACGACCCTACAACTTGTGGATTCGATTTTAATGGCGCCATGGAGGACATAAGCCGGATCCCTGAAAAAAGTGTTATCATGCTTCACGCTTGCGCTCACAACCCAACCGGAGTCGATCCCAAGGATGAACAATGGAAAGAAATGTCACAATTGGTCAAGAAAAGGAATTTGTTTGCTTTCTTTGACATGGCTTATCAAGGCTTTGCTTCCGGTGACGTTGACGAAGATGCCTTTGCCGTCCGCCAATTTTTGGAAGATGGGCACAATATTTGTTTGTCCCAATCGTACGCCAAAAACATGGGTCTTTATGGCGAGCGAGTAGGAGCGTTTACTGTTGTTTGCAAAGTAAGTATACGCGAAAAAAGGAAGAACCGAACTAGTAACATATTTGGTCAAATTTAGGACAAGGAAGAAGCAGCCCGCGTGAACTCTCAAATCAAGATCCTCATCAGACCCATGTATTCCAATCCTCCTGTTAATGGATCACGGATTGTGAGCGAGATTCTTACAAACACTACTTTAAACAAGCAGTGGCTGGAAGATGTGAAGGGTATGGCTGACCGAATCATTACAATGAGACAAGAATTAAAGGATGGATTGGCCAAGGAAGGATCGAGCAAAAATTGGGAGCACATTGTGGATCAAATTGGAATGTTTTGTTTCACAGGAATGACCCCTGAACAGG--TATTTGTTGTGATAATGGAAAAGGTCTCCATACTTAACTGTTTTTCTGTCTTCAGGTTGAAAAAATTACCAAGGACTTCAGTGTTTACATGACCAAAGATGGCCGTATTTCCGTGGCAGGGATTTCCTCTTCTAATGTTGGATATTTGGCTAAAGCCATG

GOT2_BH7f_B GGCCCCCCTGATCCTATTTTGGGCGTGAGCGAGGCCTTCAAAAAGGATACTAATCCCCTGAAAATGAATTTAGGGGTCGGTGCTTACCGAGATGATCAAGGCAAACCCTTTGTCTTGCCGTCTGTTCGTGAGGCCGAACGTATCATTGCTCAAAAAGGATTGAACAAAGAATACGCTCCAATTGGAGGTGAACCCGAGTTTGGCAGGCTATCCGCTAACTTAGCATTTGGCCAAGGTACGTAATCTCTGTCAAGGATGAATTGAGGAGCGTTCAGCTTAAATCAATG-AAAAAAATGGTATCATCTTCAGGAAATGAGATTGTTTCAAGTGGACGGAACGTGTCGGTTCAGACTATCTCGGGCACAGGTGCCTTGCGAGTAGGTGCCACTTATTTAGCCAAATGGTTCCCTGGGAACAAAACCGTGTATTTGCCCAGGCCTTCATGGGGCAATCATACCCCCATCTTTAAGCAAAGTGGGATGAATGTCGATGGTTATCGTTACTACGACCCTACAACTTGTGGATTCGATTTTAATGGCGCCATGGAGGACATAAGCCGGATCCCTGAAAAAAGTGTTATCATGCTTCACGCTTGCGCTCACAACCCAACCGGAGTCGATCCCAAGGATGAACAATGGAAAGAAATGTCACAATTGGTCAAGAAAAGGAATTTGTTTGCTTTCTTTGACATGGCTTATCAAGGCTTTGCTTCCGGTGACGTTGACGAAGATGCCTTTGCCGTCCGCCAATTTTTGGAAGATGGGCACAATATTTGTTTGTCCCAATCGTACGCCAAAAACATGGGTCTTTATGGCGAGCGAGTAGGAGCGTTTACTGTTGTTTGCAAAGTAAGTATACGCGAAAAAAGGAAGAACCGAACTAGTAACATATTTGGTCAAATTTAGGACAAGGAAGAAGCAGCCCGCGTGAACTCTCAAATCAAGATCCTCATCAGACCCATGTATTCCAATCCTCCTGTTAATGGATCACGGATTGTGAGCGAGATTCTTACAAACACTACTTTAAACAAGCAGTGGCTGGAAGATGTGAAGGGTATGGCTGACCGAATCATTACAATGAGACAAGAATTAAAGGATGGATTGGCCAAGGAAGGATCGAGCAAAAATTGGGAGCACATTGTGGATCAAATTGGAATGTTTTGTTTCACAGGAATGACCCCTGAACAGG--TATTTGTTGTGATAATGGAAAAGGTCTCCATACTTAACTGTTTTTCTGTCTTCAGGTTGAAAAAATTACCAAGGACTTCAGTGTTTACATGACCAAAGATGGCCGTATTTCCGTGGCAGGGATTTCCTCTTCTAATGTTGGATATTTGGCTAAAGCCATG

SD_2f_got2a GGCCCCCCTGATCCTATTTTGGGCGTGAGTGAGGCCTTCAAAAAGGATACTAATCCCCTGAAAATGAATTTAGGGGTTGGTGCTTACCGAGATGATCAAGGCAAACCCTTTGTCTTGCCGTCTGTTCGTGAGGCCGAACGGATCATTGCTCAAAAAGGATTGAACAAAGAATACGCTCCAATTGGAGGTGAACCCGAGTTTGGCAGGCTTTCCGCTAACTTAGCATTTGGCCAAGGTACGTAATCTCTGTCAAGCATGAATTGAGGAGCGTTCAGCTTAAATCAATG-AAAAAAATGGTAT---CTTCAGGAAATGAGATTGTTTCAAGTGGACGGAACGTGTCGGTTCAGACTATCTCGGGCACAGGTGCCTTGCGAGTAGGTGCCACTTATTTAGCCAAATGGTTCCCTGGGAACAAAACCGTGTATTTGCCCAGGCCTTCATGGGGCAATCATACCCCCATCTTTAAGCAAAGTGGGATGAATGTCGATGGTTATCGTTACTACGACCCTACAACTTGTGGATTCGATTTTAATGGCGCCATGGAGGACATAAGCCGGATCCCTGAAAAAAGCGTTATCATGCTTCACGCTTGCGCTCATAACCCAACCGGAGTCGATCCCAAGGATGAACAATGGAAAGAAATGTCACAATTGGTCAAGAAAAGGAATCTGTTTGCTTTCTTTGACATGGCTTATCAAGGCTTTGCTTCCGGTGACGTTGACAGAGATGCCTTTGCCGTCCGCCAATTTTTGGAAGATGGGCACAATATTTGTTTGTCCCAATCGTACGCCAAAAACATGGGTCTTTATGGCGAGCGAGTAGGAGCGTTTACTGTTGTTTGCAAAGTAAGTATACGCGAAAAAAGGAAGAACCGAACTAGTAACATGTTTGATTAAATTTAGGATAAGGAAGAAGCAGCCCGCGTGAACTCTCAAATCAAGATCCTCATTAGACCCATGTATTCCAATCCTCCTGTTAATGGATCACGGATTGTGAGCGAGATTCTTACCAACACTGCTTTAAACAAGCAGTGGCTGGAAGATGTGAAGGGTATGGCTGACCGAATCATTACAATGAGACAGGAATTAAAGGATGGGTTGGCCAATGAAGGATCGAGCAAAAATTGGGAGCACATTGTGGATCAAATTGGAATGTTTTGTTTCACTGGAATGACCCCTGAACAGG--TATATGTTGTGATAAGGGAAAAGGTCTCCATACTAAACTATTTTTCTATCTTCAGGTTGAAAAAATAACCACGGAATTTAGTGTTTACATGACCAAAGATGGCCGTATTTCAGTGGCGGGGATTTCCTCATCTAATGTTGGATATTTGGCTAAAGCCATG

SD_2f_got2b GGCCCCCCTGATCCTATTTTGGGCGTGAGTGAGGCCTTCAAAAAGGATACTAATCCCCTGAAAATGAATTTAGGGGTTGGTGCTTACCGAGATGATCAAGGCAAACCCTTTGTCTTGCCGTCTGTTCGTGAGGCCGAACGGATCATTGCTCAAAAAGGATTGAACAAAGAATACGCTCCAATTGGAGGTGAACCCGAGTTTGGCAGGCTTTCCGCTAACTTAGCATTTGGCCAAGGTACGTAATCTCTGTCAAGCATGAATTGAGGAGCGTTCAGCTTAAATCAATG-AAAAAAATGGTAT---CTTCAGGAAATGAGATTGTTTCAAGTGGACGGAACGTGTCGGTTCAGACTATCTCGGGCACAGGTGCCTTGCGAGTAGGTGCCACTTATTTAGCCAAATGGTTCCCTGGGAACAAAACCGTGTATTTGCCCAGGCCTTCATGGGGCAATCATACCCCCATCTTTAAGCAAAGTGGGATGAATGTCGATGGTTATCGTTACTACGACCCTACAACTTGTGGATTCGATTTTAATGGCGCCATGGAGGACATAAGCCGGATCCCTGAAAAAAGTGTTATCATGCTTCACGCTTGCGCTCATAACCCAACCGGAGTCGATCCCAAGGATGAACAATGGAAAGAAATGTCACAATTGGTCAAGAAAAGGAATCTGTTTGCTTTCTTTGACATGGCTTATCAAGGCTTTGCTTCCGGTGACGTTGACAGAGATGCCTTTGCCGTCCGCCAATTTTTGGAAGATGGGCACAATATTTGTTTGTCCCAATCGTACGCCAAAAACATGGGTCTTTATGGCGAGCGAGTAGGAGCGTTTACTGTTGTTTGCAAAGTAAGTATACGCGAAAAAAGGAAGAACCGAACTAGTAACATGTTTGATTAAATTTAGGATAAGGAAGAAGCAGCCCGCGTGAACTCTCAAATCAAGATCCTCATTAGACCCATGTATTCCAATCCTCCTGTTAATGGATCACGGATTGTGAGCGAGATTCTTACCAACACTGCTTTAAACAAGCAGTGGCTGGAAGATGTGAAGGGTATGGCTGACCGAATCATTACAATGAGACAGGAATTAAAGGATGGGTTGGCCAATGAAGGATCGAGCAAAAATTGGGAGCACATTGTGGATCAAATTGGAATGTTTTGTTTCACTGGAATGACCCCTGAACAGG--TATATGTTGTGATAAGGGAAAAGGTCTCCATACTAAACTATTTTTCTATCTTCAGGTTGAAAAAATAACCACGGAATTTAGTGTTTACATGACCAAAGATGGCCGTATTTCAGTGGCGGGGATTTCCTCATCTAATGTTGGATATTTGGCTAAAGCCATG

SD_4f_GOT2a GGCCCCCCTGATCCTATTTTGGGCGTGAGTGAGGCCTTCAAAAAGGATACTAATCCCCTGAAAATGAATTTAGGGGTTGGTGCTTACCGAGATGATCAAGGCAAACCCTTTGTCTTGCCGTCTGTTCGTGAGGCCGAACGGATCATTGCTCAAAAAGGATTGAACAAAGAATACGCTCCAATTGGAGGTGAACCCGAGTTTGGCAGGCTTTCCGCTAACTTAGCATTTGGCCAAGGTACGTAATCTCTGTCAAGCATGAATTGAGGAGCGTTCAGCTTAAATCAATG-AAAAAAATGGTAT---CTTCAGGAAATGAGATTGTTTCAAGTGGACGGAACGTGTCGGTTCAGACTATCTCGGGCACAGGTGCCTTGCGAGTAGGTGCCACTTATTTAGCCAAATGGTTCCCTGGGAACAAAACCGTGTATTTGCCCAGGCCTTCATGGGGCAATCATACCCCCATCTTTAAGCAAAGTGGGATGAATGTCGATGGTTATCGTTACTACGACCCTACAACTTGTGGATTCGATTTTAATGGCGCCATGGAGGACATAAGCCGGATCCCTGAAAAAAGCGTTATCATGCTTCACGCTTGCGCTCATAACCCAACCGGAGTCGATCCCAAGGATGAACAATGGAAAGAAATGTCACAATTGGTCAAGAAAAGGAATCTGTTTGCTTTCTTTGACATGGCTTATCAAGGCTTTGCTTCCGGTGACGTTGACAGAGATGCCTTTGCCGTCCGCCAATTTTTGGAAGATGGGCACAATATTTGTTTGTCCCAATCGTACGCCAAAAACATGGGTCTTTATGGCGAGCGAGTAGGAGCGTTTACTGTTGTTTGCAAAGTAAGTATACGCGAAAAAAGGAAGAACCGAACTAGTAACATGTTTGATTAAATTTAGGATAAGGAAGAAGCAGCCCGCGTGAACTCTCAAATCAAGATCCTCATTAGACCCATGTATTCCAATCCTCCTGTTAATGGATCACGGATTGTGAGCGAGATTCTTACCAACACTGCTTTAAACAAGCAGTGGCTGGAAGATGTGAAGGGTATGGCTGACCGAATCATTACAATGAGACAGGAATTAAAGGATGGGTTGGCCAATGAAGGATCGAGCAAAAATTGGGAGCACATTGTGGATCAAATTGGAATGTTTTGTTTCACTGGAATGACCCCTGAACAGG--TATATGTTGTGATAAGGGAAAAGGTCTCCATACTAAACTATTTTTCTATCTTCAGGTTGAAAAAATAACCACGGAATTTAGTGTTTACATGACCAAAGATGGCCGTATTTCAGTGGCGGGGATTTCCTCATCTAATGTTGGATATTTGGCTAAAGCCATG

SD_4f_GOT2b GGCCCCCCTGATCCTATTTTGGGCGTGAGTGAGGCCTTCAAAAAGGATACTAATCCCCTGAAAATGAATTTAGGGGTTGGTGCTTACCGAGATGATCAAGGCAAACCCTTTGTCTTGCCGTCTGTTCGTGAGGCCGAACGGATCATTGCTCAAAAAGGATTGAACAAAGAATACGCTCCAATTGGAGGTGAACCCGAGTTTGGCAGGCTTTCCGCTAACTTAGCATTTGGCCAAGGTACGTAATCTCTGTCAAGCATGAATTGAGGAGCGTTCAGCTTAAATCAATG-AAAAAAATGGTAT---CTTCAGGAAATGAGATTGTTTCAAGTGGACGGAACGTGTCGGTTCAGACTATCTCGGGCACAGGTGCCTTGCGAGTAGGTGCCACTTATTTAGCCAAATGGTTCCCTGGGAACAAAACCGTGTATTTGCCCAGGCCTTCATGGGGCAATCATACCCCCATCTTTAAGCAAAGTGGGATGAATGTCGATGGTTATCGTTACTACGACCCTACAACTTGTGGATTCGATTTTAATGGCGCCATGGAGGACATAAGCCGGATCCCTGAAAAAAGCGTTATCATGCTTCACGCTTGCGCTCATAACCCAACCGGAGTCGATCCCAAGGATGAACAATGGAAAGAAATGTCACAATTGGTCAAGAAAAGGAATCTGTTTGCTTTCTTTGACATGGCTTATCAAGGCTTTGCTTCCGGTGACGTTGACAGAGATGCCTTTGCCGTCCGCCAATTTTTGGAAGATGGGCACAATATTTGTTTGTCCCAATCGTACGCCAAAAACATGGGTCTTTATGGCGAGCGAGTAGGAGCGTTTACTGTTGTTTGCAAAGTAAGTATACGCGAAAAAAGGAAGAACCGAACTAGTAACATGTTTGATTAAATTTAGGATAAGGAAGAAGCAGCCCGCGTGAACTCTCAAATCAAGATCCTCATTAGACCCATGTATTCCAATCCTCCTGTTAATGGATCACGGATTGTGAGCGAGATTCTTACCAACACTGCTTTAAACAAGCAGTGGCTGGAAGATGTGAAGGGTATGGCTGACCGAATCATTACAATGAGACAGGAATTAAAGGATGGGTTGGCCAATGAAGGATCGAGCAAAAATTGGGAGCACATTGTGGATCAAATTGGAATGTTTTGTTTCACTGGAATGACCCCTGAACAGG--TATATGTTGTGATAAGGGAAAAGGTCTCCATACTAAACTATTTTTCTATCTTCAGGTTGAAAAAATAACCACGGAATTTAGTGTTTACATGACCAAAGATGGCCGTATTTCAGTGGCGGGGATTTCCTCATCTAATGTTGGATATTTGGCTAAAGCCATG

SD_5f_GOT2a GGCCCCCCTGATCCTATTTTGGGCGTGAGTGAGGCCTTCAAAAAGGATACTAATCCCCTGAAAATGAATTTAGGGGTTGGTGCTTACCGAGATGATCAAGGCAAACCCTTTGTCTTGCCGTCTGTTCGTGAGGCCGAACGGATCATTGCTCAAAAAGGATTGAACAAAGAATACGCTCCAATTGGAGGTGAACCCGAGTTTGGCAGGCTTTCCGCTAACTTAGCATTTGGCCAAGGTACGTAATCTCTGTCAAGCATGAATTGAGGAGCGTTCAGCTTAAATCAATG-AAAAAAATGGTAT---CTTCAGGAAATGAGATTGTTTCAAGTGGACGGAACGTGTCGGTTCAGACTATCTCGGGCACAGGTGCCTTGCGAGTAGGTGCCACTTATTTAGCCAAATGGTTCCCTGGGAACAAAACCGTGTATTTGCCCAGGCCTTCATGGGGCAATCATACCCCCATCTTTAAGCAAAGTGGGATGAATGTCGATGGTTATCGTTACTACGACCCTACAACTTGTGGATTCGATTTTAATGGCGCCATGGAGGACATAAGCCGGATCCCTGAAAAAAGCGTTATCATGCTTCACGCTTGCGCTCATAACCCAACCGGAGTCGATCCCAAGGATGAACAATGGAAAGAAATGTCACAATTGGTCAAGAAAAGGAATCTGTTTGCTTTCTTTGACATGGCTTATCAAGGCTTTGCTTCCGGTGACGTTGACAGAGATGCCTTTGCCGTCCGCCAATTTTTGGAAGATGGGCACAATATTTGTTTGTCCCAATCGTACGCCAAAAACATGGGTCTTTATGGCGAGCGAGTAGGAGCGTTTACTGTTGTTTGCAAAGTAAGTATACGCGAAAAAAGGAAGAACCGAACTAGTAACATGTTTGATTAAATTTAGGATAAGGAAGAAGCAGCCCGCGTGAACTCTCAAATCAAGATCCTCATTAGACCCATGTATTCCAATCCTCCTGTTAATGGATCACGGATTGTGAGCGAGATTCTTACCAACACTGCTTTAAACAAGCAGTGGCTGGAAGATGTGAAGGGTATGGCTGACCGAATCATTACAATGAGACAGGAATTAAAGGATGGGTTGGCCAATGAAGGATCGAGCAAAAATTGGGAGCACATTGTGGATCAAATTGGAATGTTTTGTTTCACTGGAATGACCCCTGAACAGG--TATATGTTGTGATAAGGGAAAAGGTCTCCATACTAAACTATTTTTCTATCTTCAGGTTGAAAAAATAACCACGGAATTTAGTGTTTACATGACCAAAGATGGCCGTATTTCAGTGGCGGGGATTTCCTCATCTAATGTTGGATATTTGGCTAAAGCCATG

SD_5f_GOT2b GGCCCCCCTGATCCTATTTTGGGCGTGAGTGAGGCCTTCAAAAAGGATACTAATCCCCTGAAAATGAATTTAGGGGTTGGTGCTTACCGAGATGATCAAGGCAAACCCTTTGTCTTGCCGTCTGTTCGTGAGGCCGAACGGATCATTGCTCAAAAAGGATTGAACAAAGAATACGCTCCAATTGGAGGTGAACCCGAGTTTGGCAGGCTTTCCGCTAACTTAGCATTTGGCCAAGGTACGTAATCTCTGTCAAGCATGAATTGAGGAGCGTTCAGCTTAAATCAATG-AAAAAAATGGTAT---CTTCAGGAAATGAGATTGTTTCAAGTGGACGGAACGTGTCGGTTCAGACTATCTCGGGCACAGGTGCCTTGCGAGTAGGTGCCACTTATTTAGCCAAATGGTTCCCTGGGAACAAAACCGTGTATTTGCCCAGGCCTTCATGGGGCAATCATACCCCCATCTTTAAGCAAAGTGGGATGAATGTCGATGGTTATCGTTACTACGACCCTACAACTTGTGGATTCGATTTTAATGGCGCCATGGAGGACATAAGCCGGATCCCTGAAAAAAGCGTTATCATGCTTCACGCTTGCGCTCATAACCCAACCGGAGTCGATCCCAAGGATGAACAATGGAAAGAAATGTCACAATTGGTCAAGAAAAGGAATCTGTTTGCTTTCTTTGACATGGCTTATCAAGGCTTTGCTTCCGGTGACGTTGACAGAGATGCCTTTGCCGTCCGCCAATTTTTGGAAGATGGGCACAATATTTGTTTGTCCCAATCGTACGCCAAAAACATGGGTCTTTATGGCGAGCGAGTAGGAGCGTTTACTGTTGTTTGCAAAGTAAGTATACGCGAAAAAAGGAAGAACCGAACTAGTAACATGTTTGATTAAATTTAGGATAAGGAAGAAGCAGCCCGCGTGAACTCTCAAATCAAGATCCTCATTAGACCCATGTATTCCAATCCTCCTGTTAATGGATCACGGATTGTGAGCGAGATTCTTACCAACACTGCTTTAAACAAGCAGTGGCTGGAAGATGTGAAGGGTATGGCTGACCGAATCATTACAATGAGACAGGAATTAAAGGATGGGTTGGCCAATGAAGGATCGAGCAAAAATTGGGAGCACATTGTGGATCAAATTGGAATGTTTTGTTTCACTGGAATGACCCCTGAACAGG--TATATGTTGTGATAAGGGAAAAGGTCTCCATACTAAACTATTTTTCTATCTTCAGGTTGAAAAAATAACCACGGAATTTAGTGTTTACATGACCAAAGATGGCCGTATTTCAGTGGCGGGGATTTCCTCATCTAATGTTGGATATTTGGCTAAAGCCATG

sd_f6_GOT2a GGCCCCCCTGATCCTATTTTGGGCGTGAGTGAGGCCTTCAAAAAGGATACTAATCCCCTGAAAATGAATTTAGGGGTTGGTGCTTACCGAGATGATCAAGGCAAACCCTTTGTCTTGCCGTCTGTTCGTGAGGCCGAACGGATCATTGCTCAAAAAGGATTGAACAAAGAATACGCTCCAATTGGAGGTGAACCCGAGTTTGGCAGGCTTTCCGCTAACTTAGCATTTGGCCAAGGTACGTAATCTCTGTCAAGCATGAATTGAGGAGCGTTCAGCTTAAATCAATG-AAAAAAATGGTAT---CTTCAGGAAATGAGATTGTTTCAAGTGGACGGAACGTGTCGGTTCAGACTATCTCGGGCACAGGTGCCTTGCGAGTAGGTGCCACTTATTTAGCCAAATGGTTCCCTGGGAACAAAACCGTGTATTTGCCCAGGCCTTCATGGGGCAATCATACCCCCATCTTTAAGCAAAGTGGGATGAATGTCGATGGTTATCGTTACTACGACCCTACAACTTGTGGATTCGATTTTAATGGCGCCATGGAGGACATAAGCCGGATCCCTGAAAAAAGTGTTATCATGCTTCACGCTTGCGCTCATAACCCAACCGGAGTCGATCCCAAGGATGAACAATGGAAAGAAATGTCACAATTGGTCAAGAAAAGGAATCTGTTTGCTTTCTTTGACATGGCTTATCAAGGCTTTGCTTCCGGTGACGTTGACAGAGATGCCTTTGCCGTCCGCCAATTTTTGGAAGATGGGCACAATATTTGTTTGTCCCAATCGTACGCCAAAAACATGGGTCTTTATGGCGAGCGAGTAGGAGCGTTTACTGTTGTTTGCAAAGTAAGTATACGCGAAAAAAGGAAGAACCGAACTAGTAACATGTTTGATTAAATTTAGGATAAGGAAGAAGCAGCCCGCGTGAACTCTCAAATCAAGATCCTCATTAGACCCATGTATTCCAATCCTCCTGTTAATGGATCACGGATTGTGAGCGAGATTCTTACCAACACTGCTTTAAACAAGCAGTGGCTGGAAGATGTGAAGGGTATGGCTGACCGAATCATTACAATGAGACAGGAATTAAAGGATGGGTTGGCCAATGAAGGATCGAGCAAAAATTGGGAGCACATTGTGGATCAAATTGGAATGTTTTGTTTCACTGGAATGACCCCTGAACAGG--TATATGTTGTGATAAGGGAAAAGGTCTCCATACTAAACTATTTTTCTATCTTCAGGTTGAAAAAATAACCACGGAATTTAGTGTTTACATGACCAAAGATGGCCGTATTTCAGTGGCGGGGATTTCCTCATCTAATGTTGGATATTTGGCTAAAGCCATG

sd_f6_GOT2b GGCCCCCCTGATCCTATTTTGGGCGTGAGTGAGGCCTTCAAAAAGGATACTAATCCCCTGAAAATGAATTTAGGGGTTGGTGCTTACCGAGATGATCAAGGCAAACCCTTTGTCTTGCCGTCTGTTCGTGAGGCCGAACGGATCATTGCTCAAAAAGGATTGAACAAAGAATACGCTCCAATTGGAGGTGAACCCGAGTTTGGCAGGCTTTCCGCTAACTTAGCATTTGGCCAAGGTACGTAATCTCTGTCAAGCATGAATTGAGGAGCGTTCAGCTTAAATCAATG-AAAAAAATGGTAT---CTTCAGGAAATGAGATTGTTTCAAGTGGACGGAACGTGTCGGTTCAGACTATCTCGGGCACAGGTGCCTTGCGAGTAGGTGCCACTTATTTAGCCAAATGGTTCCCTGGGAACAAAACCGTGTATTTGCCCAGGCCTTCATGGGGCAATCATACCCCCATCTTTAAGCAAAGTGGGATGAATGTCGATGGTTATCGTTACTACGACCCTACAACTTGTGGATTCGATTTTAATGGCGCCATGGAGGACATAAGCCGGATCCCTGAAAAAAGTGTTATCATGCTTCACGCTTGCGCTCATAACCCAACCGGAGTCGATCCCAAGGATGAACAATGGAAAGAAATGTCACAATTGGTCAAGAAAAGGAATCTGTTTGCTTTCTTTGACATGGCTTATCAAGGCTTTGCTTCCGGTGACGTTGACAGAGATGCCTTTGCCGTCCGCCAATTTTTGGAAGATGGGCACAATATTTGTTTGTCCCAATCGTACGCCAAAAACATGGGTCTTTATGGCGAGCGAGTAGGAGCGTTTACTGTTGTTTGCAAAGTAAGTATACGCGAAAAAAGGAAGAACCGAACTAGTAACATGTTTGATTAAATTTAGGATAAGGAAGAAGCAGCCCGCGTGAACTCTCAAATCAAGATCCTCATTAGACCCATGTATTCCAATCCTCCTGTTAATGGATCACGGATTGTGAGCGAGATTCTTACCAACACTGCTTTAAACAAGCAGTGGCTGGAAGATGTGAAGGGTATGGCTGACCGAATCATTACAATGAGACAGGAATTAAAGGATGGGTTGGCCAATGAAGGATCGAGCAAAAATTGGGAGCACATTGTGGATCAAATTGGAATGTTTTGTTTCACTGGAATGACCCCTGAACAGG--TATATGTTGTGATAAGGGAAAAGGTCTCCATACTAAACTATTTTTCTATCTTCAGGTTGAAAAAATAACCACGGAATTTAGTGTTTACATGACCAAAGATGGCCGTATTTCAGTGGCGGGGATTTCCTCATCTAATGTTGGATATTTGGCTAAAGCCATG

sd_f7_GOT2a GGCCCCCCTGATCCTATTTTGGGCGTGAGTGAGGCCTTCAAAAAGGATACTAATCCCCTGAAAATGAATTTAGGGGTTGGTGCTTACCGAGATGATCAAGGCAAACCCTTTGTCTTGCCGTCTGTTCGTGAGGCCGAACGGATCATTGCTCAAAAAGGATTGAACAAAGAATACGCTCCAATTGGAGGTGAACCCGAGTTTGGCAGGCTTTCCGCTAACTTAGCATTTGGCCAAGGTACGTAATCTCTGTCAAGCATGAATTGAGGAGCGTTCAGCTTAAATCAATG-AAAAAAATGGTAT---CTTCAGGAAATGAGATTGTTTCAAGTGGACGGAACGTGTCGGTTCAGACTATCTCGGGCACAGGTGCCTTGCGAGTAGGTGCCACTTATTTAGCCAAATGGTTCCCTGGGAACAAAACCGTGTATTTGCCCAGGCCTTCATGGGGCAATCATACCCCCATCTTTAAGCAAAGTGGGATGAATGTCGATGGTTATCGTTACTACGACCCTACAACTTGTGGATTCGATTTTAATGGCGCCATGGAGGACATAAGCCGGATCCCTGAAAAAAGCGTTATCATGCTTCACGCTTGCGCTCATAACCCAACCGGAGTCGATCCCAAGGATGAACAATGGAAAGAAATGTCACAATTGGTCAAGAAAAGGAATCTGTTTGCTTTCTTTGACATGGCTTATCAAGGCTTTGCTTCCGGTGACGTTGACAGAGATGCCTTTGCCGTCCGCCAATTTTTGGAAGATGGGCACAATATTTGTTTGTCCCAATCGTACGCCAAAAACATGGGTCTTTATGGCGAGCGAGTAGGAGCGTTTACTGTTGTTTGCAAAGTAAGTATACGCGAAAAAAGGAAGAACCGAACTAGTAACATGTTTGATTAAATTTAGGATAAGGAAGAAGCAGCCCGCGTGAACTCTCAAATCAAGATCCTCATTAGACCCATGTATTCCAATCCTCCTGTTAATGGATCACGGATTGTGAGCGAGATTCTTACCAACACTGCTTTAAACAAGCAGTGGCTGGAAGATGTGAAGGGTATGGCTGACCGAATCATTACAATGAGACAGGAATTAAAGGATGGGTTGGCCAATGAAGGATCGAGCAAAAATTGGGAGCACATTGTGGATCAAATTGGAATGTTTTGTTTCACTGGAATGACCCCTGAACAGG--TATATGTTGTGATAAGGGAAAAGGTCTCCATACTAAACTATTTTTCTATCTTCAGGTTGAAAAAATAACCACGGAATTTAGTGTTTACATGACCAAAGATGGCCGTATTTCAGTGGCGGGGATTTCCTCATCTAATGTTGGATATTTGGCTAAAGCCATG

sd_f7_GOT2b GGCCCCCCTGATCCTATTTTGGGCGTGAGTGAGGCCTTCAAAAAGGATACTAATCCCCTGAAAATGAATTTAGGGGTTGGTGCTTACCGAGATGATCAAGGCAAACCCTTTGTCTTGCCGTCTGTTCGTGAGGCCGAACGGATCATTGCTCAAAAAGGATTGAACAAAGAATACGCTCCAATTGGAGGTGAACCCGAGTTTGGCAGGCTTTCCGCTAACTTAGCATTTGGCCAAGGTACGTAATCTCTGTCAAGCATGAATTGAGGAGCGTTCAGCTTAAATCAATG-AAAAAAATGGTAT---CTTCAGGAAATGAGATTGTTTCAAGTGGACGGAACGTGTCGGTTCAGACTATCTCGGGCACAGGTGCCTTGCGAGTAGGTGCCACTTATTTAGCCAAATGGTTCCCTGGGAACAAAACCGTGTATTTGCCCAGGCCTTCATGGGGCAATCATACCCCCATCTTTAAGCAAAGTGGGATGAATGTCGATGGTTATCGTTACTACGACCCTACAACTTGTGGATTCGATTTTAATGGCGCCATGGAGGACATAAGCCGGATCCCTGAAAAAAGTGTTATCATGCTTCACGCTTGCGCTCATAACCCAACCGGAGTCGATCCCAAGGATGAACAATGGAAAGAAATGTCACAATTGGTCAAGAAAAGGAATCTGTTTGCTTTCTTTGACATGGCTTATCAAGGCTTTGCTTCCGGTGACGTTGACAGAGATGCCTTTGCCGTCCGCCAATTTTTGGAAGATGGGCACAATATTTGTTTGTCCCAATCGTACGCCAAAAACATGGGTCTTTATGGCGAGCGAGTAGGAGCGTTTACTGTTGTTTGCAAAGTAAGTATACGCGAAAAAAGGAAGAACCGAACTAGTAACATGTTTGATTAAATTTAGGATAAGGAAGAAGCAGCCCGCGTGAACTCTCAAATCAAGATCCTCATTAGACCCATGTATTCCAATCCTCCTGTTAATGGATCACGGATTGTGAGCGAGATTCTTACCAACACTGCTTTAAACAAGCAGTGGCTGGAAGATGTGAAGGGTATGGCTGACCGAATCATTACAATGAGACAGGAATTAAAGGATGGGTTGGCCAATGAAGGATCGAGCAAAAATTGGGAGCACATTGTGGATCAAATTGGAATGTTTTGTTTCACTGGAATGACCCCTGAACAGG--TATATGTTGTGATAAGGGAAAAGGTCTCCATACTAAACTATTTTTCTATCTTCAGGTTGAAAAAATAACCACGGAATTTAGTGTTTACATGACCAAAGATGGCCGTATTTCAGTGGCGGGGATTTCCTCATCTAATGTTGGATATTTGGCTAAAGCCATG

LJS_f1_GOT2a GGCCCCCCTGATCCTATTTTGGGCGTGAGCGAGGCCTTCAAAAAGGATACTAATCCCCTGAAAATGAATTTAGGGGTTGGTGCTTACCGAGATGATCAAGGCAAACCCTTTGTCTTGCCGTCTGTTCGTGAGGCCGAACGGATCATTGCTCAAAAAGGATTGAACAAAGAATACGCTCCAATTGGAGGTGAACCCGAGTTTGGCAGGCTTTCCGCTAACTTAGCATTTGGCCAAGGTACGTAATCTCTGTCAAGCATGAATTGAGGAGCGTTCAGCTTAAATCAATG-AAAAAAATGGTAT---CTTCAGGAAATGAGATTGTTTCAAGTGGACGGAACGTGTCGGTTCAGACTATCTCGGGCACAGGTGCCTTGCGAGTAGGTGCCACTTATTTAGCCAAATGGTTCCCTGGGAACAAAACCGTGTATTTGCCCAGGCCTTCATGGGGCAATCATACCCCCATCTTTAAGCAAAGTGGGATGAATGTCGATGGTTATCGTTACTACGACCCTACAACTTGTGGATTCGATTTTAATGGCGCCATGGAGGACATAAACCGGATCCCTGAAAAAAGCGTTATCATGCTTCACGCTTGCGCTCATAACCCAACCGGAGTCGATCCCAAGGATGAACAATGGAAAGAAATGTCACAATTGGTCAAGAAAAGGAATCTGTTTGCTTTCTTTGACATGGCTTATCAAGGCTTTGCTTCCGGTGACGTTGACAGAGATGCCTTTGCCGTCCGCCAATTTTTGGAAGATGGGCACAATATTTGTTTGTCCCAATCGTACGCCAAAAACATGGGTCTTTATGGCGAGCGAGTAGGAGCGTTTACTGTTGTTTGCAAAGTAAGTATACGCGAAAAAAGGAAGAACCGAACTAGTAACATGTTTGATTAAATTTAGGATAAGGAAGAAGCAGCCCGCGTGAACTCTCAAATCAAGATCCTCATTAGACCCATGTATTCCAATCCTCCTGTTAATGGATCACGGATTGTGAGCGAGATTCTTACCAACACTACTTTAAACAAGCAGTGGCTGGAAGATGTGAAGGGTATGGCTGACCGAATCATTACAATGAGACAGGAATTAAAGGATGGGTTGGCCAAGGAAGGATCGAGCAAAAATTGGGAGCACATTGTGGATCAAATTGGAATGTTTTGTTTCACAGGAATGACCCCTGAACAGG--TATATGTTGTGATAAGGGAAATGGTCTCCATAC-TAACTATTTTTCTATCTTCAGGTTGAAAAAATAACCACGGAATTTAGTGTTTACATGACCAAAGATGGCCGTATTTCCGTGGCGGGGATTTCCTCATCTAATGTTGGATATTTGGCTAAAGCCATG

LJS_f1_GOT2b GGCCCCCCTGATCCTATTTTGGGCGTGAGCGAGGCCTTCAAAAAGGATACTAATCCCCTGAAAATGAATTTAGGGGTTGGTGCTTACCGAGATGATCAAGGCAAACCCTTTGTCTTGCCGTCTGTTCGTGAGGCCGAACGGATCATTGCTCAAAAAGGATTGAACAAAGAATACGCTCCAATTGGAGGTGAACCCGAGTTTGGCAGGCTTTCTGCTAACTTAGCATTTGGCCAAGGTACGTAATCTCTGTCAAGCATGAATTGAGGAGCGTTTAGCTTAAATCAATG-AAAAAAATGGTAT---CTTCAGGAAATGAGATTGTTTCAAGTGGACGGAACGTGTCGGTTCAGACTATCTCGGGCACAGGTGCCTTGCGAGTAGGTGCCACTTATTTAGCCAAATGGTTCCCTGGGAACAAAACCGTGTATTTGCCCAGGCCTTCATGGGGCAATCATACCCCCATCTTTAAGCAAAGTGGGATGAATGTCGATGGTTATCGTTACTACGACCCTACAACTTGTGGATTCGATTTTAATGGCGCCATGGAGGACATAAATCGGATCCCTGAAAAAAGCGTTATCATGCTTCACGCTTGCGCTCATAACCCAACCGGAGTCGATCCCAAGGATGAACAATGGAAAGAAATGTCACAATTGGTCAAGAAAAGGAATCTGTTTGCTTTCTTTGACATGGCTTATCAAGGCTTTGCTTCCGGTGACGTTGACAGAGATGCCTTTGCCGTCCGCCAATTTTTGGAAGATGGGCACAATATTTGTTTGTCCCAATCGTACGCCAAAAACATGGGTCTTTATGGCGAGCGAGTAGGAGCGTTTACTGTTGTTTGCAAAGTAAGTATACGCGAAAAAAGGAAGAACCGAACTAGTAACATGTTTGATTAAATTTAGGATAAGGAAGAAGCAGCCCGCGTGAACTCTCAAATCAAGATCCTCATTAGACCCATGTATTCCAATCCTCCTGTTAATGGATCACGGATTGTGAGCGAGATTCTTACCAACACTACTTTAAACAAGCAGTGGCTGGAAGATGTGAAGGGTATGGCTGACCGAATCATTACAATGAGACAGGAATTAAAGGATGGGTTGGCCAAGGAAGGATCGAGCAAAAATTGGGAGCACATTGTGGATCAAATTGGAATGTTTTGTTTCACAGGAATGACCCCTGAACAGG--TATATGTTGTGATAAGGGAAATGGTCTCCATAC-TAACTATTTTTCTATCTTCAGGTTGAAAAAATAACCACGGAATTTAGTGTTTACATGACCAAAGATGGCCGTATTTCCGTGGCGGGGATTTCCTCATCTAATGTTGGATATTTGGCTAAAGCCATG

LJS_f2_GOT2a GGCCCCCCTGATCCTATTTTGGGCGTGAGCGAGGCCTTCAAAAAGGATACTAATCCCCTGAAAATGAATTTAGGGGTTGGTGCTTACCGAGATGATCAAGGCAAACCCTTTGTCTTGCCGTCTGTTCGTGAGGCCGAACGGATCATTGCTCAAAAAGGATTGAACAAAGAATACGCTCCAATTGGAGGTGAACCCGAGTTTGGCAGGCTTTCCGCTAACTTAGCATTTGGCCAAGGTACGTAATCTCTGTCAAGCATGAATTGAGGAGCGTTCAGCTTAAATCAATG-AAAAAAATGGTAT---CTTCAGGAAATGAGATTGTTTCAAGTGGACGGAACGTGTCGGTTCAGACTATCTCGGGCACAGGTGCCTTGCGAGTAGGTGCCACTTATTTAGCCAAATGGTTCCCTGGGAACAAAACCGTGTATTTGCCCAGGCCTTCATGGGGCAATCATACCCCCATCTTTAAGCAAAGTGGGATGAATGTCGATGGTTATCGTTACTACGACCCTACAACTTGTGGATTCGATTTTAATGGCGCCATGGAGGACATAAACCGGATCCCTGAAAAAAGCGTTATCATGCTTCACGCTTGCGCTCATAACCCAACCGGAGTCGATCCCAAGGATGAACAATGGAAAGAAATGTCACAATTGGTCAAGAAAAGGAATCTGTTTGCTTTCTTTGACATGGCTTATCAAGGCTTTGCTTCCGGTGACGTTGACAGAGATGCCTTTGCCGTCCGCCAATTTTTGGAAGATGGGCACAATATTTGTTTGTCCCAATCGTACGCCAAAAACATGGGTCTTTATGGCGAGCGAGTAGGAGCGTTTACTGTTGTTTGCAAAGTAAGTATACGCGAAAAAAGGAAGAACCGAACTAGTAACATGTTTGATTAAATTTAGGATAAGGAAGAAGCAGCCCGCGTGAACTCTCAAATCAAGATCCTCATTAGACCCATGTATTCCAATCCTCCTGTTAATGGATCACGGATTGTGAGCGAGATTCTTACCAACACTACTTTAAACAAGCAGTGGCTGGAAGATGTGAAGGGTATGGCTGACCGAATCATTACAATGAGACAGGAATTAAAGGATGGGTTGGCCAAGGAAGGATCGAGCAAAAATTGGGAGCACATTGTGGATCAAATTGGAATGTTTTGTTTCACAGGAATGACCCCTGAACAGG--TATATGTTGTGATAAGGGAAATGGTCTCCATACTTAACTATTTTTCTATCTTCAGGTTGAAAAAATAACCACGGAATTTAGTGTTTACATGACCAAAGATGGCCGTATTTCCGTGGCGGGGATTTCCTCATCTAATGTTGGATATTTGGCTAAAGCCATG

LJS_f2_GOT2b GGCCCCCCTGATCCTATTTTGGGCGTGAGCGAGGCCTTCAAAAAGGATACTAATCCCCTGAAAATGAATTTAGGGGTTGGTGCTTACCGAGATGATCAAGGCAAACCCTTTGTCTTGCCGTCTGTTCGTGAGGCCGAACGGATCATTGCTCAAAAAGGATTGAACAAAGAATACGCTCCAATTGGAGGTGAACCCGAGTTTGGCAGGCTTTCCGCTAACTTAGCATTTGGCCAAGGTACGTAATCTCTGTCAAGCATGAATTGAGGAGCGTTTAGCTTAAATCAATG-AAAAAAATGGTAT---CTTCAGGAAATGAGATTGTTTCAAGTGGACGGAACGTGTCGGTTCAGACTATCTCGGGCACAGGTGCCTTGCGAGTAGGTGCCACTTATTTAGCCAAATGGTTCCCTGGGAACAAAACCGTGTATTTGCCCAGGCCTTCATGGGGCAATCATACCCCCATCTTTAAGCAAAGTGGGATGAATGTCGATGGTTATCGTTACTACGACCCTACAACTTGTGGATTCGATTTTAATGGCGCCATGGAGGACATAAACCGGATCCCTGAAAAAAGCGTTATCATGCTTCACGCTTGCGCTCATAACCCAACCGGAGTCGATCCCAAGGATGAACAATGGAAAGAAATGTCACAATTGGTCAAGAAAAGGAATCTGTTTGCTTTCTTTGACATGGCTTATCAAGGCTTTGCTTCCGGTGACGTTGACAGAGATGCCTTTGCCGTCCGCCAATTTTTGGAAGATGGGCACAATATTTGTTTGTCCCAATCGTACGCCAAAAACATGGGTCTTTATGGCGAGCGAGTAGGAGCGTTTACTGTTGTTTGCAAAGTAAGTATACGCGAAAAAAGGAAGAACCGAACTAGTAACATGTTTGATTAAATTTAGGATAAGGAAGAAGCAGCCCGCGTGAACTCTCAAATCAAGATCCTCATTAGACCCATGTATTCCAATCCTCCTGTTAATGGATCACGGATTGTGAGCGAGATTCTTACCAACACTACTTTAAACAAGCAGTGGCTGGAAGATGTGAAGGGTATGGCTGACCGAATCATTACAATGAGACAGGAATTAAAGGATGGGTTGGCCAAGGAAGGATCGAGCAAAAATTGGGAGCACATTGTGGATCAAATTGGAATGTTTTGTTTCACAGGAATGACCCCTGAACAGG--TATATGTTGTGATAAGGGAAATGGTCTCCATAC-TAACTATTTTTCTATCTTCAGGTTGAAAAAATAACCACGGAATTTAGTGTTTACATGACCAAAGATGGCCGTATTTCCGTGGCGGGGATTTCCTCATCTAATGTTGGATATTTGGCTAAAGCCATG

LJS_3f_GOT2a GGCCCCCCTGATCCTATTTTGGGGGTGAGCGAGGCCTTCAAAAAGGATACTAATCCCCTGAAAATGAATTTAGGGGTTGGTGCTTACCGAGATGATCAAGGCAAACCCTTTGTCTTGCCGTCTGTTCGTGAGGCCGAACGGATCATTGCTCAAAAAGGATTGAACAAAGAATACGCTCCAATTGGAGGTGAACCCGAGTTTGGCAGGCTTTCCGCTAACTTAGCATTTGGCCAAGGTACGTAATCTCTGTCAAGCATGAATTGAGGAGCGTTCAGCTTAAATCAATG-AAAAAAATGGTAT---CTTCAGGAAATGAGATTGTTTCAAGTGGACGGAACGTGTCGGTTCAGACTATCTCGGGCACAGGTGCCTTGCGAGTAGGTGCCACTTATTTAGCCAAATGGTTCCCTGGGAACAAAACCGTGTATTTGCCCAGGCCTTCATGGGGCAATCATACCCCCATCTTTAAGCAAAGTGGGATGAATGTCGATGGTTATCGTTACTACGACCCTACAACTTGTGGATTCGATTTTAATGGCGCCATGGAGGACATAAACCGGATCCCTGAAAAAAGCGTTATCATGCTTCACGCTTGCGCTCATAACCCAACCGGAGTCGATCCCAAGGATGAACAATGGAAAGAAATGTCACAATTGGTCAAGAAAAGGAATCTGTTTGCTTTCTTTGACATGGCTTATCAAGGCTTTGCTTCCGGTGACGTTGACAGAGATGCCTTTGCCGTCCGCCAATTTTTGGAAGATGGGCACAATATTTGTTTGTCCCAATCGTACGCCAAAAACATGGGTCTTTATGGCGAGCGAGTAGGAGCGTTTACTGTTGTTTGCAAAGTAAGTATACGCGAAAAAAGGAAGAACCGAACTAGTAACATGTTTGATTAAATTTAGGATAAGGAAGAAGCAGCCCGCGTGAACTCTCAAATCAAGATCCTCATTAGACCCATGTATTCCAATCCTCCTGTTAATGGATCACGGATTGTGAGCGAGATTCTTACCAACACTACTTTAAACAAGCAGTGGCTGGAAGATGTGAAGGGTATGGCTGACCGAATCATTACAATGAGACAGGAATTAAAGGATGGGTTGGCCAAGGAAGGATCGAGCAAAAATTGGGAGCACATTGTGGATCAAATTGGAATGTTTTGTTTCACAGGAATGACCCCTGAACAGG--TATATGTTGTGATAAGGGAAATGGTCTCCATAC-TAACTATTTTTCTATCTTCAGGTTGAAAAAATAACCACGGAATTTAGTGTTTACATGACCAAAGATGGCCGTATTTCCGTGGCGGGGATTTCCTCATCTAATGTTGGATATTTGGCTAAAGCCATG

LJS_3f_GOT2b GGCCCCCCTGATCCTATTTTGGGCGTGAGCGAGGCCTTCAAAAAGGATACTAATCCCCTGAAAATGAATTTAGGGGTTGGTGCTTACCGAGATGATCAAGGCAAACCCTTTGTCTTGCCGTCTGTTCGTGAGGCCGAACGGATCATTGCTCAAAAAGGATTGAACAAAGAATACGCTCCAATTGGAGGTGAACCCGAGTTTGGCAGGCTTTCCGCTAACTTAGCATTTGGCCAAGGTACGTAATCTCTGTCAAGCATGAATTGAGGAGCGTTTAGCTTAAATCAATG-AAAAAAATGGTAT---CTTCAGGAAATGAGATTGTTTCAAGTGGACGGAACGTGTCGGTTCAGACTATCTCGGGCACAGGTGCCTTGCGAGTAGGTGCCACTTATTTAGCCAAATGGTTCCCTGGGAACAAAACCGTGTATTTGCCCAGGCCTTCATGGGGCAATCATACCCCCATCTTTAAGCAAAGTGGGATGAATGTCGATGGTTATCGTTACTACGACCCTACAACTTGTGGATTCGATTTTAATGGCGCCATGGAGGACATAAATCGGATCCCTGAAAAAAGCGTTATCATGCTTCACGCTTGCGCTCATAACCCAACCGGAGTCGATCCCAAGGATGAACAATGGAAAGAAATGTCACAATTGGTCAAGAAAAGGAATCTGTTTGCTTTCTTTGACATGGCTTATCAAGGCTTTGCTTCCGGTGACGTTGACAGAGATGCCTTTGCCGTCCGCCAATTTTTGGAAGATGGGCACAATATTTGTTTGTCCCAATCGTACGCCAAAAACATGGGTCTTTATGGCGAGCGAGTAGGAGCGTTTACTGTTGTTTGCAAAGTAAGTATACGCGAAAAAAGGAAGAACCGAACTAGTAACATGTTTGATTAAATTTAGGATAAGGAAGAAGCAGCCCGCGTGAACTCTCAAATCAAGATCCTCATTAGACCCATGTATTCCAATCCTCCTGTTAATGGATCACGGATTGTGAGCGAGATTCTTACCAACACTACTTTAAACAAGCAGTGGCTGGAAGATGTGAAGGGTATGGCTGACCGAATCATTACAATGAGACAGGAATTAAAGGATGGGTTGGCCAAGGAAGGATCGAGCAAAAATTGGGAGCACATTGTGGATCAAATTGGAATGTTTTGTTTCACAGGAATGACCCCTGAACAGG--TATATGTTGTGATAAGGGAAATGGTCTCCATAC-TAACTATTTTTCTATCTTCAGGTTGAAAAAATAACCACGGAATTTAGTGTTTACATGACCAAAGATGGCCGTATTTCCGTGGCGGGGATTTCCTCATCTAATGTTGGATATTTGGCTAAAGCCATG

LJS_4f_GOT2a GGCCCCCCTGATCCTATTTTGGGCGTGAGCGAGGCCTTCAAAAAGGATACTAATCCCCTGAAAATGAATTTAGGGGTTGGTGCTTACCGAGATGATCAAGGCAAACCCTTTGTCTTGCCGTCTGTTCGTGAGGCCGAACGGATCATTGCTCAAAAAGGATTGAACAAAGAATACGCTCCAATTGGAGGTGAACCCGAGTTTGGCAGGCTTTCCGCTAACTTAGCATTTGGCCAAGGTACGTAATCTCTGTCAAGCATGAATTGAGGAGCGTTCAGCTTAAATCAATG-AAAAAAATGGTAT---CTTCAGGAAATGAGATTGTTTCAAGTGGACGGAACGTGTCGGTTCAGACTATCTCGGGCACAGGTGCCTTGCGAGTAGGTGCCACTTATTTAGCCAAATGGTTCCCTGGGAACAAAACCGTGTATTTGCCCAGGCCTTCATGGGGCAATCATACCCCCATCTTTAAGCAAAGTGGGATGAATGTCGATGGTTATCGTTACTACGACCCTACAACTTGTGGATTCGATTTTAATGGCGCCATGGAGGACATAAACCGGATCCCTGAAAAAAGCGTTATCATGCTTCACGCTTGCGCTCATAACCCAACCGGAGTCGATCCCAAGGATGAACAATGGAAAGAAATGTCACAATTGGTCAAGAAAAGGAATCTGTTTGCTTTCTTTGACATGGCTTATCAAGGCTTTGCTTCCGGTGACGTTGACAGAGATGCCTTTGCCGTCCGCCAATTTTTGGAAGATGGGCACAATATTTGTTTGTCCCAATCGTACGCCAAAAACATGGGTCTCTATGGCGAGCGAGTAGGAGCGTTTACTGTTGTTTGCAAAGTAAGTATACGCGAAAAAAGGAAGAACCGAACTAGTAACATGTTTGATTAAATTTAGGATAAGGAAGAAGCAGCCCGCGTGAACTCTCAAATCAAGATCCTCATTAGACCCATGTATTCCAATCCTCCTGTTAATGGATCACGGATTGTGAGCGAGATTCTTACCAACACTACTTTAAACAAGCAGTGGCTGGAAGATGTGAAGGGTATGGCTGACCGAATCATTACAATGAGACAGGAATTAAAGGATGGGTTGGCCAAGGAAGGATCGAGCAAAAATTGGGAGCACATTGTGGATCAAATTGGAATGTTTTGTTTCACAGGAATGACCCCTGAACAGG--TATATGTTGTGATAAGGGAAATGGTCTCCATAC-TAACTATTTTTCTATCTTCAGGTTGAAAAAATAACCACGGAATTTAGTGTTTACATGACCAAAGATGGCCGTATTTCCGTGGCGGGGATTTCCTCATCTAATGTTGGATATTTGGCTAAAGCCATG

LJS_4f_GOT2b GGCCCCCCTGATCCTATTTTGGGCGTGAGCGAGGCCTTCAAAAAGGATACTAATCCCCTGAAAATGAATTTAGGGGTTGGTGCTTACCGAGATGATCAAGGCAAACCCTTTGTCTTGCCGTCTGTTCGTGAGGCCGAACGGATCATTGCTCAAAAAGGATTGAACAAAGAATACGCTCCAATTGGAGGTGAACCCGAGTTTGGCAGGCTTTCCGCTAACTTAGCATTTGGCCAAGGTACGTAATCTCTGTCAAGCATGAATTGAGGAGCGTTCAGCTTAAATCAATG-AAAAAAATGGTAT---CTTCAGGAAATGAGATTGTTTCAAGTGGACGGAACGTGTCGGTTCAGACTATCTCGGGCACAGGTGCCTTGCGAGTAGGTGCCACTTATTTAGCCAAATGGTTCCCTGGGAACAAAACCGTGTATTTGCCCAGGCCTTCATGGGGCAATCATACCCCCATCTTTAAGCAAAGTGGGATGAATGTCGATGGTTATCGTTACTACGACCCTACAACTTGTGGATTCGATTTTAATGGCGCCATGGAGGACATAAACCGGATCCCTGAAAAAAGCGTTATCATGCTTCACGCTTGCGCTCATAACCCAACCGGAGTCGATCCCAAGGATGAACAATGGAAAGAAATGTCACAATTGGTCAAGAAAAGGAATCTGTTTGCTTTCTTTGACATGGCTTATCAAGGCTTTGCTTCCGGTGACGTTGACAGAGATGCCTTTGCCGTCCGCCAATTTTTGGAAGATGGGCACAATATTTGTTTGTCCCAATCGTACGCCAAAAACATGGGTCTTTATGGCGAGCGAGTAGGAGCGTTTACTGTTGTTTGCAAAGTAAGTATACGCGAAAAAAGGAAGAACCGAACTAGTAACATGTTTGATTAAATTTAGGATAAGGAAGAAGCAGCCCGCGTGAACTCTCAAATCAAGATCCTCATTAGACCCATGTATTCCAATCCTCCTGTTAATGGATCACGGATTGTGAGCGAGATTCTTACCAACACTACTTTAAACAAGCAGTGGCTGGAAGATGTGAAGGGTATGGCTGACCGAATCATTACAATGAGACAGGAATTAAAGGATGGGTTGGCCAAGGAAGGATCGAGCAAAAATTGGGAGCACATTGTGGATCAAATTGGAATGTTTTGTTTCACAGGAATGACCCCTGAACAGG--TATATGTTGTGATAAGGGAAATGGTCTCCATAC-TAACTATTTTTCTATCTTCAGGTTGAAAAAATAACCACGGAATTTAGTGTTTACATGACCAAAGATGGCCGTATTTCCGTGGCGGGGATTTCCTCATCTAATGTTGGATATTTGGCTAAAGCCATG

;

endblock;
