# Supplementary material for: Complex Deleterious Interactions Associated with Malic Enzyme May Contribute to Reproductive Isolation in the Copepod Tigriopus californicus
Source: PLoS One. 2011 Jun 22;6(6):e21177. doi: 10.1371/journal.pone.0021177 (PMC3120845; doi:10.1371/journal.pone.0021177)
Supplement: File S2 — Alignment of aspartate aminotransferase homologs with T. californicus GOT2 predicted protein. (DOC) [file pone.0021177.s006.doc]

**Supplemental sequence FILE S2**

**Alignment of aspartate aminotransferase homologs with *T. californicus* GOT2 predicted protein**

#NEXUS

BEGIN TAXA;

TITLE Taxa;

DIMENSIONS NTAX=27;

TAXLABELS

NP_725534_GOT1B_D_melanogaster XP_313023_Anopheles_gambiae ACO15246_cytAAT_Caligus_clemen ACO11819_cytAAT_Lepeophtheirus ACN51888_cytAAT_Daphnia_magna NCBI_GNO_342583_Dpulex_cytAAT_ XP_969549_cytAAT_Tribolium_cas NP_002070_cytAAT_Homo_sapiens AAH45269_Xenopus_laevis ACO11582_ProbcytAAT_Caligus_ro ACO12265_12221_comb_prob_cytAA NP_002071_mtAAT_Homo_sapiens AAH56110_Got2_Xenopus_laevis TcalGot2full ACO10233_mtATT_Caligus_rogercr NP_722744_GOT2A_D_melanogaster XP_318743_Anopheles_gambiae XP_967224_mtAAT_Tribolium_cast NCBI_GNO_436713_Dpulex_mtAAT_D XP_002409595_mtAAT_Ixodes_scap XP_396131_mtAAT_Apis_mellifera NP_001040337_mtAAT_Bombyx_mori NP_180654_mtAAT_Arabidopsis_th P46645_cytAAT_Arabidopsis_thal P46644_cpAAT_Arabidopsis_thal NP_849838_cytAAT_iso2_Arabidop P00509_ECOLI_ASPAT

;

END;

BEGIN CHARACTERS;

TITLE Character_Matrix;

DIMENSIONS NCHAR=509;

FORMAT DATATYPE = Protein INTERLEAVE GAP = - MISSING = X;

MATRIX

[1 50]

NP_725534_GOT1B_D_melanogaster ------------------------MFIILKRLNYPGLLQNFQLSTMSIYA

XP_313023_Anopheles_gambiae ---------------------------VLRKCATT-FQQIPPTATMSIFA

ACO15246_cytAAT_Caligus_clemen --------------------------------------------MASLFA

ACO11819_cytAAT_Lepeophtheirus ---------------------------------------------MSLFS

ACN51888_cytAAT_Daphnia_magna -----------------------------------------------KFA

NCBI_GNO_342583_Dpulex_cytAAT_ -------------------------------------------MATSKFA

XP_969549_cytAAT_Tribolium_cas ---------------------------------------------MSRFA

NP_002070_cytAAT_Homo_sapiens ------------------------------------------MAPPSVFA

AAH45269_Xenopus_laevis ------------------------------------------MA--SIFC

ACO11582_ProbcytAAT_Caligus_ro -----------------------------------------MPGSSSSFS

ACO12265_12221_comb_prob_cytAA -----------------------------------------MPTTSSFFS

NP_002071_mtAAT_Homo_sapiens ---------------MALLHSGRVLPGIAAAFHPGLAAAASARASS-WWT

AAH56110_Got2_Xenopus_laevis ---------------MALLKS-RLLVGVAR-CQPCLAVVQG-RASS-WWS

TcalGot2full -----------MNKSSSILKRSSLLGQSIPWQPIKMGAPLSARASS-WWS

ACO10233_mtATT_Caligus_rogercr ---------------MATPSL-RHLSSSLL-INKPAMGMSSVRAGS-WWK

NP_722744_GOT2A_D_melanogaster ---------------MSQICKRGLLISN-------RLAPAALRCKSTWFS

XP_318743_Anopheles_gambiae ----------------------------------------------SWWS

XP_967224_mtAAT_Tribolium_cast ---------------MSLKKTVNLLVIR-------KNGVIKQRSS-SWWS

NCBI_GNO_436713_Dpulex_mtAAT_D ---------MALISSLKFFSPSTLTTAQ-------NALSVSSRNS-SWWS

XP_002409595_mtAAT_Ixodes_scap --------------------------------------------------

XP_396131_mtAAT_Apis_mellifera ----------MAQNKIKFAFSYNLFNKN-------KVALYGIRSMGTWWP

NP_001040337_mtAAT_Bombyx_mori ---------------MAQVLKKLTTQVLKPNNVDTIVGCTGLRASSTWWN

NP_180654_mtAAT_Arabidopsis_th ---------------MALAMMIRNAASKRG-MTPISGHFGGLRSMSSWWK

P46645_cytAAT_Arabidopsis_thal --------------------------------------------MDSVFS

P46644_cpAAT_Arabidopsis_thal MKTTHFSSSSSSDRRIGALLRHLNSGSDSDNLSSLYASPTSGGTGGSVFS

NP_849838_cytAAT_iso2_Arabidop --------------------------------------------MNSILS

P00509_ECOLI_ASPAT -----------------------------------------------MFE

[51 100]

NP_725534_GOT1B_D_melanogaster DVPKGPAIEVFALTQAFKDDSNPNKVNLSVGAYRTDAGVPWVLPVVRKTE

XP_313023_Anopheles_gambiae SVELGPPVEVFALNKACNDDPNPNKVNLGVGAYRTNEGKPWILPVVKKAE

ACO15246_cytAAT_Caligus_clemen EVSAAPPIEVFKLSKDFREDPTEKKVSLGVGAYRDETGKPWVLPVVKKME

ACO11819_cytAAT_Lepeophtheirus EVPAAPPIEVFKLSRDFREDPCDKKVSLGVGAYRDDEGKPWVLPVVKKME

ACN51888_cytAAT_Daphnia_magna SVESAPPIEVFALNKAYVEDTFPQKVNLGVGAYRTDEGKPWVLPVVRHME

NCBI_GNO_342583_Dpulex_cytAAT_ FVESAPPIEVFALNKAYLDDTFPQKVNLGVGAYRTDEGKPWVLPVVRQME

XP_969549_cytAAT_Tribolium_cas KVEQGPPIEVFALNQQFAADTSPNKVSLGVGAYRTNEGQPWVLPVVREAE

NP_002070_cytAAT_Homo_sapiens EVPQAQPVLVFKLTADFREDPDPRKVNLGVGAYRTDDCHPWVLPVVKKVE

AAH45269_Xenopus_laevis SVPQAPPVAVFKLTADFRADSDARKVNLGVGAYRTDDSQPWVLPVVKKVE

ACO11582_ProbcytAAT_Caligus_ro SIPVGDPVEVTEMCREYSLDKHPKKANISIGGYYGEDGGIYNFKVAQKAE

ACO12265_12221_comb_prob_cytAA TIPMGASVEVTEMCREYQLDKNPLKANLSIGGYYGEDGDIYKFRVVTKAE

NP_002071_mtAAT_Homo_sapiens HVEMGPPDPILGVTEAFKRDTNSKKMNLGVGAYRDDNGKPYVLPSVRKAE

AAH56110_Got2_Xenopus_laevis HVEMGPPDPILGVTEAFKRDTNPKKMNLGVGAYRDDNGKPYVLSSVRKAE

TcalGot2full GVEMGPPDPILGVSEAFKKDTNPLKMNLGVGAYRDDQGKPFVLPSVREAE

ACO10233_mtATT_Caligus_rogercr GVEMGPPDAILGVTEAFKKDANPKKMNLGVGAYRDDEGKPFIVPSVRKAE

NP_722744_GOT2A_D_melanogaster EVQMGPPDAILGVTEAFKKDTNPKKINLGAGAYRDDNTQPFVLPSVREAE

XP_318743_Anopheles_gambiae GVQMGPPDVILGVTEAYKRDTNPKKINLGVGAYRDDSGKPFVLPSVKKAE

XP_967224_mtAAT_Tribolium_cast QVAMGPPDAILGVTEAFKRDTNPNKINLGVGAYRDDNGKPYVLPSVRKAE

NCBI_GNO_436713_Dpulex_mtAAT_D HVEMGPPDPILGVTEAFKRDTNPKKMNLGVGAYRDDNGKPFILPSVKLAE

XP_002409595_mtAAT_Ixodes_scap ---MGPPDAILGVTEAFKKDTNPKKMNLGVGAYRDDTGKPYVLPSVRKAE

XP_396131_mtAAT_Apis_mellifera HVKMGPPDAILGLTEAYKKDQNPNKVNLGVGAYRDDNGKPFVLPSVRKAE

NP_001040337_mtAAT_Bombyx_mori NVQMGPPDVILGITEAYKKDTHPKKVNLGVGAYRDDEGKPFVLPSVRKAE

NP_180654_mtAAT_Arabidopsis_th SVEPAPKDPILGVTEAFLADPSPEKVNVGVGAYRDDNGKPVVLECVREAE

P46645_cytAAT_Arabidopsis_thal NVARAPEDPILGVTVAYNNDPSPVKINLGVGAYRTEEGKPLVLDVVRKAE

P46644_cpAAT_Arabidopsis_thal HLVQAPEDPILGVTVAYNKDPSPVKLNLGVGAYRTEEGKPLVLNVVRKAE

NP_849838_cytAAT_iso2_Arabidop SVLPAPEDPVLSVIFACRDDPSPVKLNLSAGTYRTEEGKPLVLDVVRRAE

P00509_ECOLI_ASPAT NITAAPADPILGLADLFRADERPGKINLGIGVYKDETGKTPVLTSVKKAE

[101 150]

NP_725534_GOT1B_D_melanogaster ISIASD---EQVNHEYLPVTGLETFTNAATELVLGADSPAIK-----ENR

XP_313023_Anopheles_gambiae AAIVAD---GSLNHEYLPVLGMDSITNAASTLLLGDGSEALA-----SKR

ACO15246_cytAAT_Caligus_clemen KKLHEDIDMEVINHEYLPILGLESFSAAATKMLLGEDSPAIK-----EGR

ACO11819_cytAAT_Lepeophtheirus KKLHEDIDKNSINHEYLPILGLEPFSTAATKMLLGTNSKAIQ-----EGR

ACN51888_cytAAT_Daphnia_magna QKLAAD---ETLNKEYLPVLGYEPLASAATRMLLGSDSASLK-----EGR

NCBI_GNO_342583_Dpulex_cytAAT_ QQLAAD---ETLNKEYLPVLGFEPLASAATRMLLGSDSPSLK-----EGR

XP_969549_cytAAT_Tribolium_cas KILAAD---EKLNKEYLPVLGLDTFSSAATQMLLGADSPALK-----ENR

NP_002070_cytAAT_Homo_sapiens QKIAND---NSLNHEYLPILGLAEFRSCASRLALGDDSPALK-----EKR

AAH45269_Xenopus_laevis QMIAND---NSLNHEYLPILGLPEFRSSASRIALGDDSPAFK-----EDR

ACO11582_ProbcytAAT_Caligus_ro KELIQS-----PNHSYLPPNGLPELNKAVFQLAYGHSEEAWK-----RG-

ACO12265_12221_comb_prob_cytAA RELIQS-----PNHSYLPPNGIPELNHAVFKLAFGESEKAWK-----HG-

NP_002071_mtAAT_Homo_sapiens AQIAAK---NLD-KEYLPIGGLAEFCKASAELALGENSEVLK-----SGR

AAH56110_Got2_Xenopus_laevis AQLASK---NLD-KEYLPIGGLAEFARASAQLALGENCEAVK-----NGQ

TcalGot2full RIIAQK---GLN-KEYAPIGGEPEFGRLSANLAFGQGNEIVS-----SGR

ACO10233_mtATT_Caligus_rogercr EKIFNA---DMN-HEYSTIVGDATFNKLSAELAFG--SEVVS-----EGR

NP_722744_GOT2A_D_melanogaster KRVVSR---SLD-KEYATIIGIPEFYNKAIELALGKGSKRLA-----AKH

XP_318743_Anopheles_gambiae QRLAQK---QLD-HEYSPIGGTAEFCKHSILLALGESSEHVA-----NGL

XP_967224_mtAAT_Tribolium_cast EKLRAK---NLD-KEYAPISGIAEFCKAAIELALGANSEIVS-----NGL

NCBI_GNO_436713_Dpulex_mtAAT_D EEIQKK---NMD-KEYSPISGSPEFCKATINLALGEDNEWTK-----NGL

XP_002409595_mtAAT_Ixodes_scap EILMSR---KQD-KEYLPIGGLGDFCTAAAQLAFGEDSPVLK-----NKL

XP_396131_mtAAT_Apis_mellifera EKIKTK---NMD-KEYAPIAGSSDFCKQSIKLALGDNSDVVK-----NGL

NP_001040337_mtAAT_Bombyx_mori EILHSR---GLN-HEYAPISGEATYTDAVAKLAFGEDSPVIK-----NKS

NP_180654_mtAAT_Arabidopsis_th KRLAGS---TF--MEYLPMGGSAKMVDLTLKLAYGDNSEFIK-----DKR

P46645_cytAAT_Arabidopsis_thal QQLVND---PSRVKEYIPIVGISDFNKLSAKLILGADSPAIT-----ESR

P46644_cpAAT_Arabidopsis_thal QQLIND---RTRIKEYLPIVGLVEFNKLSAKLILGADSPAIR-----ENR

NP_849838_cytAAT_iso2_Arabidop QQLAND---LSRDKEYLPLNGLPEFNKLSTKLILGDDSPALK-----ENR

P00509_ECOLI_ASPAT QYLLEN----ETTKNYLGIDGIPEFGRCTQELLFGKGSALIN-----DKR

[151 200]

NP_725534_GOT1B_D_melanogaster AFGVQTISGTGALRVAADFLHTQLN-----RNVVYYSNPTWENHHKIFCD

XP_313023_Anopheles_gambiae AFGVQCLSGTGALRLGAEFLARILH-----RTVFYYSDPTWENHHKVFLY

ACO15246_cytAAT_Caligus_clemen AFGVQSLSGTGALRNGAEFCCKMLK-----HTVFYVSTPTWGNHNSIFLK

ACO11819_cytAAT_Lepeophtheirus AFGVQSLSGTGALRNGAEFCNKMLK-----QTVFYVSTPTWGNHNSIFLK

ACN51888_cytAAT_Daphnia_magna ATGIQCLSGTGALRVGAEFLAHIGK-----HSIVYSSNPTWGNHSLVFLN

NCBI_GNO_342583_Dpulex_cytAAT_ ATGIQCLSGTGALRVGAEFLAHIGK-----HTVVYSSNPTWGNHSLVFLS

XP_969549_cytAAT_Tribolium_cas AFGVQCLSGTGCLRVGAEFLARTVG-----YKTFYVSIPTWENHKLVFTN

NP_002070_cytAAT_Homo_sapiens VGGVQSLGGTGALRIGADFLARWYNGTNNKNTPVYVSSPTWENHNAVFSA

AAH45269_Xenopus_laevis VGGVQSLGGTGALRIGAEFLRRWYNGNNNTATPIYISSPSWENHNAVFMD

ACO11582_ProbcytAAT_Caligus_ro -FCVQSIGGTGPLRLGAEFLLEHLN-----LKTAYYSDPTWINHKYIFER

ACO12265_12221_comb_prob_cytAA -FCVQSIGGTGPLRLGAEFLREHLD-----LKTAYYSDPTWINHKHIFER

NP_002071_mtAAT_Homo_sapiens FVTVQTISGTGALRIGASFLQRFFKF----SRDVFLPKPTWGNHTPIFRD

AAH56110_Got2_Xenopus_laevis FITVQTISGTGSLRIGANFLQRFYKY----SRDVYLPKPSWGNHTPIFRD

TcalGot2full NVSVQTISGTGALRVGATYLAKWFPG----NKTVYLPRPSWGNHTPIFKQ

ACO10233_mtATT_Caligus_rogercr HVTTQAISGTGALRIGAAYLSKWFPG----NKTVYLPKPSWGNHTPIFKH

NP_722744_GOT2A_D_melanogaster NVTAQSISGTGALRIGAAFLAKFWQG----NREIYIPSPSWGNHVAIFEH

XP_318743_Anopheles_gambiae NATVQGISGTGALRIGGAFLASFFPG----PKDIYLPTPSWGNHGPIFRH

XP_967224_mtAAT_Tribolium_cast NATVQGISGTGSLRVGAAFFSNFYPG----IKTVYLPKPTWGNHTPIFKH

NCBI_GNO_436713_Dpulex_mtAAT_D NATVQGISGTGSLRIGTSFLSAFFPG----NKDLYMPTPTWGNHVPLAKH

XP_002409595_mtAAT_Ixodes_scap NTTVQGISGTGSLMIGAFFLGQFFKG----NREIYMPTPTWGNHIPLFKR

XP_396131_mtAAT_Apis_mellifera NATVQGVSGTGSLYIGSLFLSQFFSS----NKEIYVPKPTWGNHSQIFRL

NP_001040337_mtAAT_Bombyx_mori NCTVQTLSGTGALRLGLEFITKHYAK----AKEIWLPTPTWGNHPQICNT

NP_180654_mtAAT_Arabidopsis_th IAAVQTLSGTGACRLFADFQKRFSPG----SQ-IYIPVPTWSNHHNIWKD

P46645_cytAAT_Arabidopsis_thal VTTVQCLSGTGSLRVGAEFLKTHYH-----QSVIYIPKPTWGNHPKVFNL

P46644_cpAAT_Arabidopsis_thal ITTVECLSGTGSLRVGGEFLAKHYH-----QKTIYITQPTWGNHPKIFTL

NP_849838_cytAAT_iso2_Arabidop VVTTQCLSGTGSLRVGAEFLATHNK-----ESVIFVPNPTWGNHPRIFTL

P00509_ECOLI_ASPAT ARTAQTPGGTGALRVAADFLAKNTS-----VKRVWVSNPSWPNHKSVFNS

[201 250]

NP_725534_GOT1B_D_melanogaster AGFTTVKSYRYWDQNK-RELDFKNMLADLND-APPG--------AVIILH

XP_313023_Anopheles_gambiae AGFTEPRTYRYWHQET-RAIDFAGMLEDLEQ-APEG--------AVVILH

ACO15246_cytAAT_Caligus_clemen SGFTEARKYRYWNNES-KGFDFEGMIEDLKN-APQN--------SVIILH

ACO11819_cytAAT_Lepeophtheirus SGFLEARKYRYWNNES-KGFDFEGMMEDLKN-APEN--------AVIILH

ACN51888_cytAAT_Daphnia_magna AGFTSYRSYRYWDAAK-KALDFDGLMEDLRN-APEN--------SVIILH

NCBI_GNO_342583_Dpulex_cytAAT_ AGFSTYKSYRYWDAAK-KALDFDGLMEDLRN-APAN--------SVILLH

XP_969549_cytAAT_Tribolium_cas AGFNDIKEYRYWSSES-RGLDLKGFLEDLNK-APEN--------SVIILH

NP_002070_cytAAT_Homo_sapiens AGFKDIRSYRYWDAEK-RGLDLQGFLNDLEN-APEF--------SIVVLH

AAH45269_Xenopus_laevis AGFKDIRAYRYWDAAK-RGLDLEGFLQDLEN-APEF--------SIFLLH

ACO11582_ProbcytAAT_Caligus_ro AGFSKVEPYPFWNYES-SEIDFCKFTHFLSKKAETG--------SVIILH

ACO12265_12221_comb_prob_cytAA AGFTQVEPYPHWNYDT-YEIDFEKLCHFLKFEAKEK--------SVIILH

NP_002071_mtAAT_Homo_sapiens AGMQLQG-YRYYDPKT-CGFDFTGAVEDISK-IPEQ--------SVLLLH

AAH56110_Got2_Xenopus_laevis AGLEVKG-YRYYDPKT-CGFDFAGALDDLSK-IPEQ--------SIILFH

TcalGot2full SGMNVDG-YRYYDPTT-CGFDFKGAMEDIR--IPEK--------SVIMLH

ACO10233_mtATT_Caligus_rogercr CGMEVGG-YRYYDPKT-CGFDFEGTCEDIKA-IPEK--------SVILLH

NP_722744_GOT2A_D_melanogaster AGLPVNR-YRYYDKDT-CALDFGGLIEDLKK-IPEK--------SIVLLH

XP_318743_Anopheles_gambiae SGLNVKA-YRYYDPST-CGFDFAGALEDLSK-IPEK--------SIVLLH

XP_967224_mtAAT_Tribolium_cast AGMDVQS-YTFYDPKT-CGLDFKGALDDINK-IPER--------SIILLH

NCBI_GNO_436713_Dpulex_mtAAT_D AGLGVKQ-YRYYDPKT-CGFDFHGALQDIAK-IPER--------SMILLH

XP_002409595_mtAAT_Ixodes_scap AGLTVKQ-YRYYDPKT-CGFDFSGALQDIAK-IPEG--------SIILLH

XP_396131_mtAAT_Apis_mellifera AGLPMKF-YRYYDPKT-CGLDFNGALEDLSK-IPGK--------SIVLFH

NP_001040337_mtAAT_Bombyx_mori LNLPHKK-YRYFDPKT-NGFDLQGALEDISK-IPEG--------SIILLH

NP_180654_mtAAT_Arabidopsis_th AQVPQKT-YHYYHPET-KGLDFSALMDDVKN-APEG--------SFFLLH

P46645_cytAAT_Arabidopsis_thal AGLSVEY-FRYYDPAT-RGLDFKGLLEDLGA-APSG--------AIVLLH

P46644_cpAAT_Arabidopsis_thal AGLTVKT-YRYYDPAT-RGLNFQGLLEDLGA-AAPG--------SIVLLH

NP_849838_cytAAT_iso2_Arabidop AGLSVQY-FRYYDPKS-RGLDFKGMLEDLGA-APPG--------AIVVLQ

P00509_ECOLI_ASPAT AGLEVRE-YAYYDAEN-HTLDFDALINSLNE-AQAG--------DVVLFH

[251 300]

NP_725534_GOT1B_D_melanogaster ACAHNPTGIDPTQEQWTELADLMEKKKLFPLFDSAYQGFASGDPDRDAWA

XP_313023_Anopheles_gambiae ACAHNPTGIDPTEDQWKQIADVCEKRKLFPFFDSAYQGFASGDPNKDAFA

ACO15246_cytAAT_Caligus_clemen AVAHNPTGIDPTQDQWKAIADVMEEKKLFPFFDCAYQGFASGDLDKDAWA

ACO11819_cytAAT_Lepeophtheirus AVAHNPTGIDPTQEQWKAIADIMQERKLFPFFDCAYQGFASGDLDKDAWA

ACN51888_cytAAT_Daphnia_magna ACAHNPTGVDPTQDQWRQIADLIEERXLFPFFDSAYQGFASGDLDRDAWA

NCBI_GNO_342583_Dpulex_cytAAT_ ACAHNPTGVDPTQDQWKQIADLIEERGLFPFFDSAYQGFASGDLDRDAWA

XP_969549_cytAAT_Tribolium_cas SCAHNPTGCDPTQEQWAQIADVMEKRKLFPFFDSAYQGFASGDLEKDAWT

NP_002070_cytAAT_Homo_sapiens ACAHNPTGIDPTPEQWKQIASVMKHRFLFPFFDSAYQGFASGNLERDAWA

AAH45269_Xenopus_laevis ACAHNPTGTDPTPDEWRKIADVMKRRSLFPFFDSAYQGFASGSLDKDAWA

ACO11582_ProbcytAAT_Caligus_ro ASVHNPTGMNFSREQWESVGHIISERGLFPFFDLAYHGFGDGGLDEDAFP

ACO12265_12221_comb_prob_cytAA ASVHNPTGMNLSKEQRKIVRDIVRERALFPFFDLAYHGFGDGGLNEDSFP

NP_002071_mtAAT_Homo_sapiens ACAHNPTGVDPRPEQWKEIATVVKKRNLFAFFDMAYQGFASGDGDKDAWA

AAH56110_Got2_Xenopus_laevis ACAHNPTGVDPKQEQWKELAALCKSRRLFPFFDMAYQGFASGDTDRDAWA

TcalGot2full ACAHNPTGVDPKDEQWKEMSQLVKKRNLFAFFDMAYQGFASGDVDRDAFA

ACO10233_mtATT_Caligus_rogercr ACAHNPTGVDPKPEQWKELSALIKKKNLYVFFDMAYQGFASGNVDGDAFA

NP_722744_GOT2A_D_melanogaster ACAHNPTGVDPTLEQWREISALVKKRNLYPFIDMAYQGFATGDIDRDAQA

XP_318743_Anopheles_gambiae ACAHNPTGVDPKPEQWAEMSALIKKRNLFPFFDMAYQGFASGDVDKDALA

XP_967224_mtAAT_Tribolium_cast ACAHNPTGVDPNLDQWAELSSLIKQRNLFPFFDMAYQGFASGDIDRDAQA

NCBI_GNO_436713_Dpulex_mtAAT_D ACAHNPTGVDPKPEQWAEMSKVIKEKKLFPFFDMAYQGFASGDIDKDATP

XP_002409595_mtAAT_Ixodes_scap ACAHNPTGVDPKFEQWKEISRIIKSRRLFPFLDMAYQGFATGDIDRDAAA

XP_396131_mtAAT_Apis_mellifera ACAHNPTGVDPNQDQWKELAETVKRRNLFPFFDMAYQGFASGSLENDAFA

NP_001040337_mtAAT_Bombyx_mori ACAHNPTGVDPKPSDWEQLSKVIKERKLFPFFDMAYQGFATGDVDNDAFA

NP_180654_mtAAT_Arabidopsis_th ACAHNPTGVDPTEEQWREISQLFKAKKHFAFFDMAYQGFASGDPARDAKS

P46645_cytAAT_Arabidopsis_thal ACAHNPTGVDPTSEQWEQIRQLMRSKSLLPFFDSAYQGFASGSLDTDAQS

P46644_cpAAT_Arabidopsis_thal ACAHNPTGVDPTIQQWEQIRKLMRSKGLMPFFDSAYQGFASGSLDTDAKP

NP_849838_cytAAT_iso2_Arabidop ACAHNPTGVDPTFEQWEKIRRLVRSKSLLPFFDSAYQGFASGSLDADAQA

P00509_ECOLI_ASPAT GCCHNPTGIDPTLEQWQTLAQLSVEKGWLPLFDFAYQGFARG-LEEDAEG

[301 350]

NP_725534_GOT1B_D_melanogaster ARYFV------QRGFELFICQSFAKNFGLYCERTG----NLAVVQ-----

XP_313023_Anopheles_gambiae VRYFV------SRGFELFCAQSFAKNFGLYNERIG----NLTVVQ-----

ACO15246_cytAAT_Caligus_clemen VRYFVQ-----ERGFEIFCAQSFSKNFGLYNERCG----NLTVVL-----

ACO11819_cytAAT_Lepeophtheirus VRYFAD-----DRGFELFCAQSFSKNFGLYNERCG----NLSFVL-----

ACN51888_cytAAT_Daphnia_magna VRYFD------SRGFEMVCAQSFAKNFGLYNERVG----NLTFVA-----

NCBI_GNO_342583_Dpulex_cytAAT_ VRYFD------SRGFEMVCAQSFAKNFGLYNERVG----NLTFVA-----

XP_969549_cytAAT_Tribolium_cas VRYFV------SRGFELLCAQSFAKNFGLYNERVG----NLTFVT-----

NP_002070_cytAAT_Homo_sapiens IRYFV------SEGFEFFCAQSFSKNFGLYNERVG----NLTVVG-----

AAH45269_Xenopus_laevis VRFFV------SQGFELFCAQSFSKNFGLYNERVG----NLTVVG-----

ACO11582_ProbcytAAT_Caligus_ro LRLFM------QKNIEFFVSQSFGKNLGLYGERIG----FLSGSI-----

ACO12265_12221_comb_prob_cytAA LRLFK------SSNIEFFVSQSFGKNLGLYGERIG----FLSGSI-----

NP_002071_mtAAT_Homo_sapiens VRHFI------EQGINVCLCQSYAKNMGLYGERVG----AFTMVC-----

AAH56110_Got2_Xenopus_laevis VRHFI------QEGINLVLSQSYAKNMGLYGERVG----AFTVVC-----

TcalGot2full VRQFL------EDGHNICLSQSYAKNMGLYGERVG----AFTVVC-----

ACO10233_mtATT_Caligus_rogercr VRQFL------KDGHDICLAQSYAKNMGLYGERIG----AFTVVC-----

NP_722744_GOT2A_D_melanogaster VRTFE------ADGHDFCLAQSFAKNMGLYGERAG----AFTVLC-----

XP_318743_Anopheles_gambiae VREFL------RDGHQIALAQSFAKNMGLYGERAG----AFSLVT-----

XP_967224_mtAAT_Tribolium_cast VRLFI------KEGHKIVLAQSFAKNMGLYGERAG----AFTVTT-----

NCBI_GNO_436713_Dpulex_mtAAT_D VRMFL------KDGHQIALSQSYAKNMGLYGERAG----AFSLIC-----

XP_002409595_mtAAT_Ixodes_scap VRLFA------EEGHGFAMAQSFAKNMGLYGERTG----AFTLVC-----

XP_396131_mtAAT_Apis_mellifera VRYFV------KNGIDIMLAQSYAKNMGLYGERVG----ALSIIT-----

NP_001040337_mtAAT_Bombyx_mori VRLFV------KEGHQVMLAQSFAKNMGLYGERAG----ALTFLC-----

NP_180654_mtAAT_Arabidopsis_th IRIFL------EDGHHIGISQSYAKNMGLYGQRVG----CLSVLC-----

P46645_cytAAT_Arabidopsis_thal VRTFV------ADGGECLIAQSYAKNMGLYGERVG----ALSIVC-----

P46644_cpAAT_Arabidopsis_thal IRMFV------ADGGECLVAQSYAKNMGLYGERVG----ALSIVC-----

NP_849838_cytAAT_iso2_Arabidop VRMFV------ADGGECLIAQSYAKNMGLYGERIG----SLTIVC-----

P00509_ECOLI_ASPAT LRAFA------AMHKELIVASSYSKNFGLYNERVG----ACTLVA-----

[351 400]

NP_725534_GOT1B_D_melanogaster --------KNGATKAAVHSQLTLLIRGQYSNPPAYGARIVSKVLNTPELR

XP_313023_Anopheles_gambiae --------KEASTSAAVASQITLLVRGMYSNPPAFGSRIVSRVLNDTELR

ACO15246_cytAAT_Caligus_clemen --------KNSDNVVNINSQLTVIIRGAYSNPPAHGCRIVDGVLNDKALY

ACO11819_cytAAT_Lepeophtheirus --------KSSDNIVNINSQLTVIIRGAYSNPPAHGCRIVEGVLNDSNLY

ACN51888_cytAAT_Daphnia_magna --------KDRSVIEPVRSQITLLVRANYSNPPNHGARIVGTVLNDPVLT

NCBI_GNO_342583_Dpulex_cytAAT_ --------KDRAVIEPVRSQITLLVRANYSNPPNHGARIVGTVLNNPALT

XP_969549_cytAAT_Tribolium_cas --------KTTDVIPKVKSQVTLLVRGMYSNPPSHGARIVAHVLSDPKLF

NP_002070_cytAAT_Homo_sapiens --------KEPESILQVLSQMEKIVRITWSNPPAQGARIVASTLSNPELF

AAH45269_Xenopus_laevis --------KDGDNVARVLSQMEKIVRTTWSNPPSQGARIVATTLNTPELF

ACO11582_ProbcytAAT_Caligus_ro --------NDENAVPRIYSQVINISRPMYSNPARHGAAILAKVLLDPSNC

ACO12265_12221_comb_prob_cytAA --------NDENSIPKIYSQMINVSRPMYSNPARYGAAILAKVLLDPSNF

NP_002071_mtAAT_Homo_sapiens --------KDADEAKRVESQLKILIRPMYSNPPLNGARIAAAILNTPDLR

AAH56110_Got2_Xenopus_laevis --------SDAEEAKRVESQIKILIRPMYSNPPLNGARIAASILTQPDLR

TcalGot2full --------KDKEEAARVNSQIKILIRPMYSNPPVNGSRIVSEILTNTALN

ACO10233_mtATT_Caligus_rogercr --------QDKEEASRVASQIKILIRPMYSNPPIHGARVVSQILSDASLR

NP_722744_GOT2A_D_melanogaster --------SDEEEAARVMSQVKILIRGLYSNPPVHGARIAAEILNNEDLR

XP_318743_Anopheles_gambiae --------GSKDEADRTMSQIKILIRPMYSNPPIHGARLVAEILGDKSLR

XP_967224_mtAAT_Tribolium_cast --------ESQEETARVMSQLKILIRALYSNPPINGARIVAEILTDPALR

NCBI_GNO_436713_Dpulex_mtAAT_D --------SSKEEAAATMSQLKIIIRPMYSNPPVTGARIATEILTTPSIR

XP_002409595_mtAAT_Ixodes_scap --------GSKEEADRCLSQIKIIVRPTYSNPPLHGARIAHLILTDQELR

XP_396131_mtAAT_Apis_mellifera --------SNKEEADRVLSQLKIIIRPAYSNPPINGARIVNEILEDSDLR

NP_001040337_mtAAT_Bombyx_mori --------GDEATAAKVMSQVKIMVRVMYSNPPLYGARLVQEILTNAELK

NP_180654_mtAAT_Arabidopsis_th --------EDPKQAVAVKSQLQQLARPMYSNPPLHGAQLVSTILEDPELK

P46645_cytAAT_Arabidopsis_thal --------KSADVASKVESQVKLVVRPMYSSPPIHGASIVATILKSSDMY

P46644_cpAAT_Arabidopsis_thal --------KSADVAGRVESQLKLVIRPMYSSPPIHGASIVAVILRDKNLF

NP_849838_cytAAT_iso2_Arabidop --------TSEDVAKKVENQVLLVVRPMYLTPPIHGASIVATILKNSDMY

P00509_ECOLI_ASPAT --------ADSETVDRAFSQMKAAIRANYSNPPAHGASVVATILSNDALR

[401 450]

NP_725534_GOT1B_D_melanogaster KEWMASIQAMSSRIREMRTALRDKLVALG-TPGTWDHIVNQIGMFSYTGL

XP_313023_Anopheles_gambiae SEWMECIKTMSSRIITMRKALYDELVALK-TPGTWEHITNQIGMFSYTGL

ACO15246_cytAAT_Caligus_clemen DEWKQSIRTMSGRIISMRQGLRERLEKLN-TPGTWNHITDQIGMFSFTGL

ACO11819_cytAAT_Lepeophtheirus NEWKESIKIMSGRIMSMRQGLRERLEKLN-TPGKWNHITDQIGMFSFTGM

ACN51888_cytAAT_Daphnia_magna EQWKSHIKTMADRIISMRLGLRERLEKLE-TPGTWNHITDQIGMFSFTGL

NCBI_GNO_342583_Dpulex_cytAAT_ EQWKGHIKTMADRIISMRHGLRERLEKME-TPGTWNHITDQIGMFSFTGL

XP_969549_cytAAT_Tribolium_cas EQWKGCIRTMATRIIEMRKALRAALEKLN-TPGDWSHITAQIGMFSYTGL

NP_002070_cytAAT_Homo_sapiens EEWTGNVKTMADRILTMRSELRARLEALK-TPGTWNHITDQIGMFSFTGL

AAH45269_Xenopus_laevis DEWRDNVKTMAERVLLMRAELKSRLEALK-TPGTWNHIVNQIGMFSYTGL

ACO11582_ProbcytAAT_Caligus_ro ESWTQELAQVRERLRSIRSQMKSILEDLTPTR-DWSGITKQSGMFYLSGL

ACO12265_12221_comb_prob_cytAA TSWVQELKEVRERLRLIRKQMKNNLDKLAPQL-NWSGITQQTGMFYFSNL

NP_002071_mtAAT_Homo_sapiens KQWLQEVKGMADRIIGMRTQLVSNLKKEG-STHNWQHITDQIGMFCFTGL

AAH56110_Got2_Xenopus_laevis KEWLQEVKGMANRIISMREQLVSNLKKEG-SIHNWQHISDQIGMFCFTGL

TcalGot2full KQWLEDVKGMADRIITMRQELKDGLANEG-SSKNWEHIVDQIGMFCFTGM

ACO10233_mtATT_Caligus_rogercr EEWLKDVKGMADRIIKMRHQLKEGLEREG-SSHNWNHITEQIGMFCFTGM

NP_722744_GOT2A_D_melanogaster AQWLKDVKLMADRIIDVRTKLKDNLIKLG-SSQNWDHIVNQIGMFCFTGL

XP_318743_Anopheles_gambiae QEWLGDVKLMADRIISVRSSLRNNLKELG-SSRNWSHITDQIGMFCFTGM

XP_967224_mtAAT_Tribolium_cast ADWLKEVKGMADRIISVRTKLRDNLKKEG-STKNWQHITDQIGMFCYTGM

NCBI_GNO_436713_Dpulex_mtAAT_D SQWLKDVKGMADRIISMRQLLRSNLAKEG-SSRDWAHITDQIGMFCFTGM

XP_002409595_mtAAT_Ixodes_scap QQWLKDVKGMADRIIGMRTRLRDGLTREG-SSRNWQHITDQIGMFCFTGM

XP_396131_mtAAT_Apis_mellifera KQWLIDVKTMADRIISMRQTLTDNLRKCG-STRDWSHITNQIGMFCFTGL

NP_001040337_mtAAT_Bombyx_mori KQWLGDVKQMADRIITMRSQLRAGIEGAG-NPHPWQHITDQIGMFCFTGL

NP_180654_mtAAT_Arabidopsis_th SLWLKEVKVMADRIIGMRTTLRESLEKLG-SPLSWEHVTKQIGMFCYSGL

P46645_cytAAT_Arabidopsis_thal NNWTIELKEMADRIKSMRQQLFEAIQARG-TPGDWSHIIKQIGMFTFTGL

P46644_cpAAT_Arabidopsis_thal NEWTLELKAMADRIISMRKQLFEALRTRG-TPGDWSHIIKQIGMFTFTGL

NP_849838_cytAAT_iso2_Arabidop NDWTIELKGMADRIISMRQQLYAALEARG-TPGDWSHIIKHIGMFTFTGL

P00509_ECOLI_ASPAT AIWEQELTDMRQRIQRMRQLFVNTLQEKG-ANRDFSFIIKQNGMFSFSGL

[451 500]

NP_725534_GOT1B_D_melanogaster NESHVRVLIDQYHIYLLKT-GRISMSGLNKGNVEYVAKAIHAAVTGSGES

XP_313023_Anopheles_gambiae NEKQVQILMKEFSIYLLKT-GRISMCGLNESNVAYVAKAIHAAVTRE---

ACO15246_cytAAT_Caligus_clemen TPDMVAFLVKEKHIYLLSN-GRISVAGLTPSNIDYVAESMNEAVNKFK--

ACO11819_cytAAT_Lepeophtheirus NPDMVSYLVKEKHIYLLSN-GRISVAGLNPSNIDYVAESMNEAVNKFQ--

ACN51888_cytAAT_Daphnia_magna GPLAVDKLIADHHIYLLKG-GRINMCGLNTGNIDYVAKCIHEVVTTTQEA

NCBI_GNO_342583_Dpulex_cytAAT_ GPLAVDKLIAEHHIYLLKG-GRINMCGLNTGNIDYVAKCIHEVVTTTQEA

XP_969549_cytAAT_Tribolium_cas TEKQSLHMVEKHHIYMLKS-GRISMCGVTPGNVDYVAKAIYETVTNVK--

NP_002070_cytAAT_Homo_sapiens NPKQVEYLVNEKHIYLLPS-GRINVSGLTTKNLDYVATSIHEAVTKIQ--

AAH45269_Xenopus_laevis NPKQVEYLIKEKHIYLMAS-GRINMCGLTTKNIDYVAQSIYEASTKIQ--

ACO11582_ProbcytAAT_Caligus_ro DMKQGLRLKEEFHIYMLPSSGRINLGAVNSNNIEYICQSLACVVR-----

ACO12265_12221_comb_prob_cytAA EKKQGLQLKEDFHVYILPSSGRINLGGINSNNIDYVCQSLAKIVSIKS--

NP_002071_mtAAT_Homo_sapiens KPEQVERLIKEFSIYMTKD-GRISVAGVTSSNVGYLAHAIHQVTK-----

AAH56110_Got2_Xenopus_laevis RPEQVERLIKEFSIYMTKD-GRISVAGVTSANNGYLAHAIHQVTK-----

TcalGot2full TPEQVEKITTEFSVYMTKD-GRISVAGISSSNVGYLAKAMHAVTK-----

ACO10233_mtATT_Caligus_rogercr TPEQVAKIMGDHSVYLTKD-GRISVAGISSGNVEYLAHAMHAVTK-----

NP_722744_GOT2A_D_melanogaster KPEQVQKLIKDHSVYLTND-GRVSMAGVTSKNVEYLAESIHKVTK-----

XP_318743_Anopheles_gambiae NQQQCERLSKEFSVYLTKD-GRISMAGVTSKNVGYLAEAIHAVTK-----

XP_967224_mtAAT_Tribolium_cast TPDQVEKITKEHSVFLTKD-GRISMAGVTSKNVEYLAHAMHTVTK-----

NCBI_GNO_436713_Dpulex_mtAAT_D APAQVEKLTKDFSVYLTKD-GRISVAGITSKNVEYLAHAMHQVTK-----

XP_002409595_mtAAT_Ixodes_scap TQEQVAKLTKDFSVYLTKD-GRISVAGISSGNVTTLRMPCIKSPSEAAAK

XP_396131_mtAAT_Apis_mellifera KSSEAEKLIRDYSIYLTKD-GRISVAGVTTKNVEYVAEAMHNVTK-----

NP_001040337_mtAAT_Bombyx_mori KPEQVERLTKEFHVYLTKD-GRISVAGISSQNVNYIAEAIHKVTS-----

NP_180654_mtAAT_Arabidopsis_th TPEQVDRLTSEYHIYMTRN-GRISMAGVTTGNVGYLANAIHEVTKSS---

P46645_cytAAT_Arabidopsis_thal NKEQVEFMTKEFHIYMTSD-GRISMAGLSSKTVPHLADAMHAAVTRLG--

P46644_cpAAT_Arabidopsis_thal NPAQVSFMTKEYHIYMTSD-GRISMAGLSSKTVPHLADAIHAVVTKAV--

NP_849838_cytAAT_iso2_Arabidop SEEQVRLMAKEYHIYMTYD-GRISMASLSSKTVPQLADAIHAVVTRIA--

P00509_ECOLI_ASPAT TKEQVLRLREEFGVYAVAS-GRVNVAGMTPDNMAPLCEAIVAVL------

[501 509]

NP_725534_GOT1B_D_melanogaster ASCPCENKL

XP_313023_Anopheles_gambiae ---------

ACO15246_cytAAT_Caligus_clemen ---------

ACO11819_cytAAT_Lepeophtheirus ---------

ACN51888_cytAAT_Daphnia_magna SL-------

NCBI_GNO_342583_Dpulex_cytAAT_ SL-------

XP_969549_cytAAT_Tribolium_cas ---------

NP_002070_cytAAT_Homo_sapiens ---------

AAH45269_Xenopus_laevis ---------

ACO11582_ProbcytAAT_Caligus_ro ---------

ACO12265_12221_comb_prob_cytAA ---------

NP_002071_mtAAT_Homo_sapiens ---------

AAH56110_Got2_Xenopus_laevis ---------

TcalGot2full ---------

ACO10233_mtATT_Caligus_rogercr ---------

NP_722744_GOT2A_D_melanogaster ---------

XP_318743_Anopheles_gambiae ---------

XP_967224_mtAAT_Tribolium_cast ---------

NCBI_GNO_436713_Dpulex_mtAAT_D ---------

XP_002409595_mtAAT_Ixodes_scap RAGCG----

XP_396131_mtAAT_Apis_mellifera ---------

NP_001040337_mtAAT_Bombyx_mori ---------

NP_180654_mtAAT_Arabidopsis_th ---------

P46645_cytAAT_Arabidopsis_thal ---------

P46644_cpAAT_Arabidopsis_thal ---------

NP_849838_cytAAT_iso2_Arabidop ---------

P00509_ECOLI_ASPAT ---------

;

END;
